# Optineurin downregulation induces endoplasmic reticulum stress, chaperone-mediated autophagy, and apoptosis in pancreatic cancer cells

**DOI:** 10.1038/s41420-019-0206-2

**Published:** 2019-08-09

**Authors:** Doaa M. Ali, Shariq S. Ansari, Michael Zepp, Michaela Knapp-Mohammady, Martin R. Berger

**Affiliations:** 1 Toxicology and Chemotherapy Unit, German Cancer Research Center, Heidelberg, Germany; 2 Department of Pharmacology and Experimental Therapeutics, Medical Research Institute, Alexandria University, Alexandria, Egypt; 3 Cell and Molecular Biology, St. Jude Children’s Research Hospital, Memphis, TN USA; 4 Division of Functional Genome Analysis, German Cancer Research Center, Heidelberg, Germany

**Keywords:** Gastrointestinal cancer, Preclinical research

## Abstract

Pancreatic ductal adenocarcinoma (PDAC) shows a high level of basal autophagy. Here we investigated the role of optineurin (OPTN) in PDAC cell lines, which is a prominent member of the autophagy system. To that purpose, mining of publically available databases showed that OPTN is highly expressed in PDAC and that high levels of expression are related to reduced survival. Therefore, the role of OPTN on proliferation, migration, and colony formation was investigated by transient knockdown in Miapaca, BXPC3, and Suit2-007 human PDAC cells. Furthermore, gene expression modulation in response to OPTN knockdown was assessed by microarray. The influence on cell cycle distribution and cell death signaling cascades was followed by FACS, assays for apoptosis, RT-PCR, and western blot. Finally, autophagy and ROS induction were screened by acridine orange and DCFH-DA fluorescent staining respectively. OPTN knockdown caused significant inhibition of colony formation, increased migration and no significant effect on proliferation in Miapaca, BXPC3 and Suit2-007 cells. The microarray showed modulation of 293 genes in Miapaca versus 302 in Suit2-007 cells, of which 52 genes overlapped. Activated common pathways included the ER stress response and chaperone-mediated autophagy, which was confirmed at mRNA and protein levels. Apoptosis was activated as shown by increased levels of cleaved PARP, Annexin V binding and nuclear fragmentation. OPTN knockdown caused no increased vacuole formation as assessed by acridine orange. Also, there was only marginally increased ROS production. Combination of OPTN knockdown with the autophagy inducer erufosine or LY294002, an inhibitor of autophagy, showed additive effects, which led us to hypothesize that they address different pathways. In conclusion, OPTN knockdown was related to activation of ER stress response and chaperone-mediated autophagy, which tend to confine the damage caused by OPTN knockdown and thus question its value for PDAC therapy.

## Introduction

**Fig. 1:**
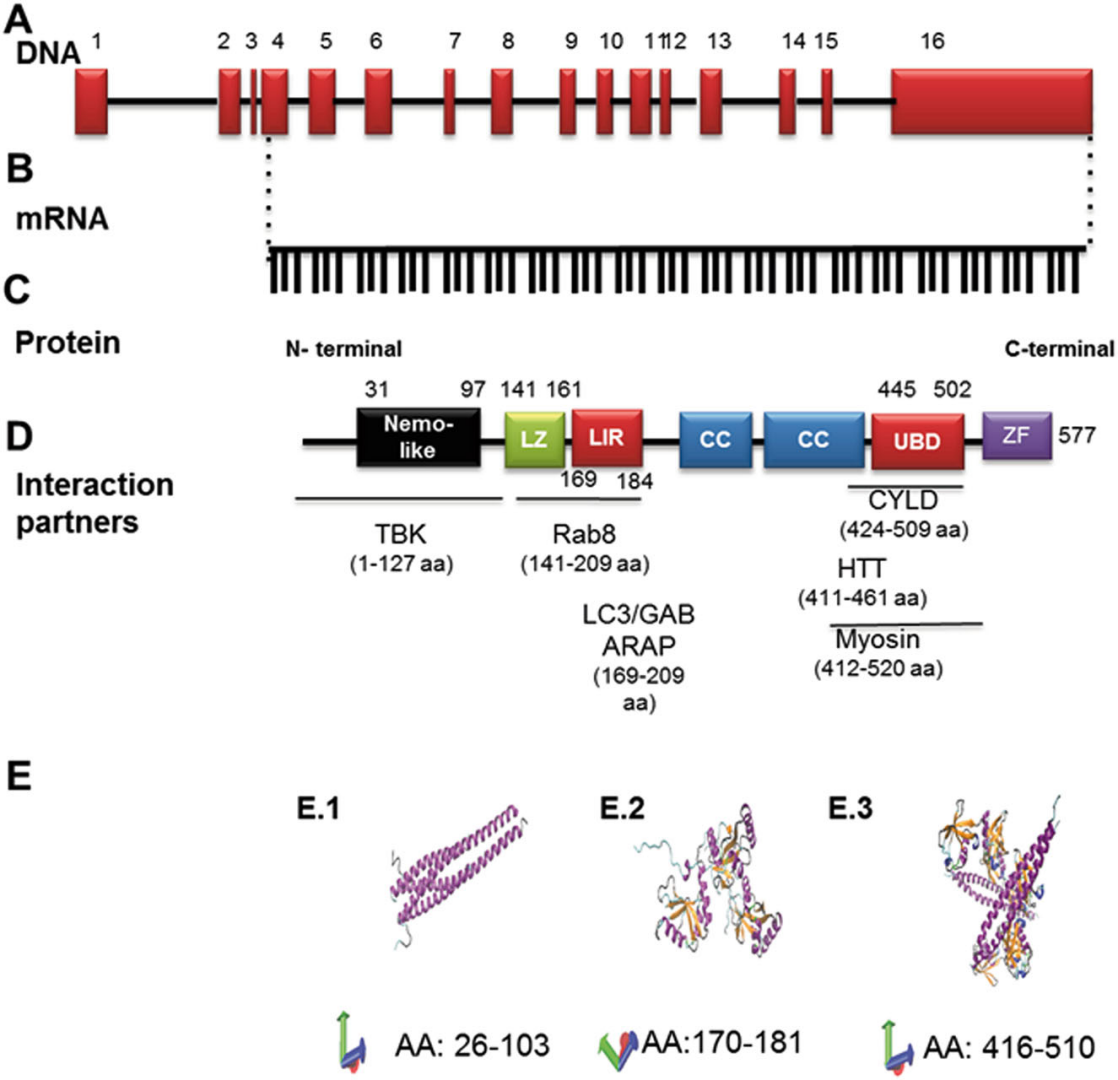
Structure of optineurin. Graphic illustration of optineurin at genomic (**a**), mRNA (**b**), and protein levels (**c**, **e**). **a** The OPTN structure at genomic level was generated using the NCBI sequence (reference NG_012876.1 (https://www.ncbi.nlm.nih.gov/nuccore/257743476)), and the scaling factors used were 1:400 bp (for exons) and 1:4000 bp (for introns). Together, 16 exons form the OPTN gene. **b** The mRNA was generated from the same source and consists of 1734 bp. **c** The protein contains various structural domains, and the localization of these domains is indicated relative to the amino acid sequence as well as to some interacting proteins (**d**). LZ Leucine-zipper domain, LIR LC3-interacting region, CC coiled coil domain, UBD ubiquitin-binding domain, ZF zinc finger domain, TBK tank binding kinase, Rab8 RAB8A, Member RAS Oncogene Family, LC3/GABARAP light chain 3/beta, GABA type A receptor associated protein. CYLD CYLD Lysine 63 Deubiquitinase, HTT huntingtin. **e** Crystal structure of different OPTN domains as determined by *X*-ray analysis. These images were made with VMD, which is owned by the theoretical and computational biophysics group, NIH Center for Macromolecular Modeling and Bioinformatics, at the Beckman Institute, University of Illinois at Urbana-Champaign. E.1^14^ represents the structure of the amino acids 26–103 (HLAHP NLDTFTPEEL LQQMKELLTE NHQLKEAMKL NNQAMKGRFE ELSAWTEKQK EERQFFEIQS KEAKERLMAL SHE). E.2^15^ represents OPTN amino acids from 170 to 181 or LC3B interacting region (the sequence consists of SGSSEDSFVE I). E.3^16^ shows the crystal structure of UBD spanning the amino acids 416–510 (EKVDR AVLKELSEKL ELAEKALASK QLQMDEMKQ TIAKQEEDLE TMTILRAQME VYCSDFHAER AAREKIHEEK EQLALQL AVL LKENDAFEDG)

Optineurin (OPTN) is encoded by a gene on chromosome 10p13, which has 16 exons, 3 of which are noncoding. After translation the protein consists of 577 amino acids (Fig. 1a, b)^1,2^, has an apparent size of a 67 kDa, a half-life of ~8 h, and its turnover involves the ubiquitin proteasomal system^3^. OPTN consists of several domains: a Nemo NF-κB-essential molecule -like domain, leucine zipper motif, an LC3-interacting region, coiled-coil motifs, an ubiquitin-binding domain, and a C-terminal C2H2 type zinc finger motif^4,5^ (Fig. 1c). OPTN interacts with itself to form homo-oligomers^6^ as well as with other molecules including Ras-related protein 8 (Rab8)^7^, huntingtin^8^, myosin VI^9^, transferrin receptor^10^, LC3/GABARAP^2^, polo-like kinase 1^11,12^, TANK binding kinase 1^13^ as well as many others^4^ (Fig. 1d). The conformation of certain parts of OPTN has been determined by X-ray analysis (Fig. 1e)^14–16^.

OPTN has several physiological roles including membrane trafficking, maintenance of the Golgi apparatus, exocytosis and protein secretion, cell division control, suppression of the NF-κB pathway, and host defense against pathogens^1,2,4,17^. Overexpression of OPTN was protective against H_2_O_2_ mediated cell death^7^.

OPTN was identified as a selective autophagy receptor because it binds to polyubiquitinated cargoes and brings them to autophagosomes via its LC3-interacting region^4,18–20^. Thus, it can get rid of pathogens as salmonella^18^, defective mitochondria^20^, misfolded protein aggregates^21^ or can have a role in tumor suppression^19^. The role of OPTN in autophagy can be also independent from ubiquitination^4^.

Beside all the physiological roles, mutations of OPTN or its altered expression are associated with multiple diseases, including normal tension glaucoma and primary open-angle glaucoma^22^, as well as plenty of neurodegenerative diseases^23^. Diminished OPTN expression might increase the risk of developing Crohn’s disease^24^ and predisposes to the occurrence of Paget’s disease by enhancing osteoclast differentiation, as OPTN was identified as a regulator of bone resorption^25^. Increased expression has been found in several cancers, including pancreatic cancer (for details see “Results” section).

Pancreatic cancers are suspected to become the second leading cause of cancer-related deaths in the United States by the year of 2030^26^. Pancreatic ductal adenocarcinoma (PDAC) accounts for 90% of all diagnosed pancreatic cancers^27^. PCAC has a poor prognosis with a mortality rate that is almost equal to the incidence rate and a 5-year survival rate of only 7% for all stages^28^.

Surgery remains the only potentially curative treatment for nonmetastatic PDAC^29,30^. Combination chemotherapy or targeted therapy cause only modest improvement in survival^29,31,32^. This background is reason for modern strategies pending on new biomarker identification and patient selection^31^.

The role of autophagy in PDAC is quite complex, with several studies indicating that autophagy has a tumor suppressive role and others reporting an onco-stimulatory effect^33^. An exciting point about PDAC is that primary tumors and cell lines show elevated basal autophagy^34^, yet the role of autophagy in PDAC is still to be clarified^35^.

Here, we investigated the role of OPTN in PDAC cells. After transient knockdown, alterations in cellular properties were observed including proliferation, migration, and colony formation as well as induction of autophagy and apoptosis. Furthermore, OPTN expression of PDAC patients was used from the Human Protein Atlas database. Our results hint to OPTN as a versatile factor in the progression of cancer in general and of PDAC in particular.

## Results

### Optineurin is overexpressed in pancreatic cancer

**Fig. 2:**
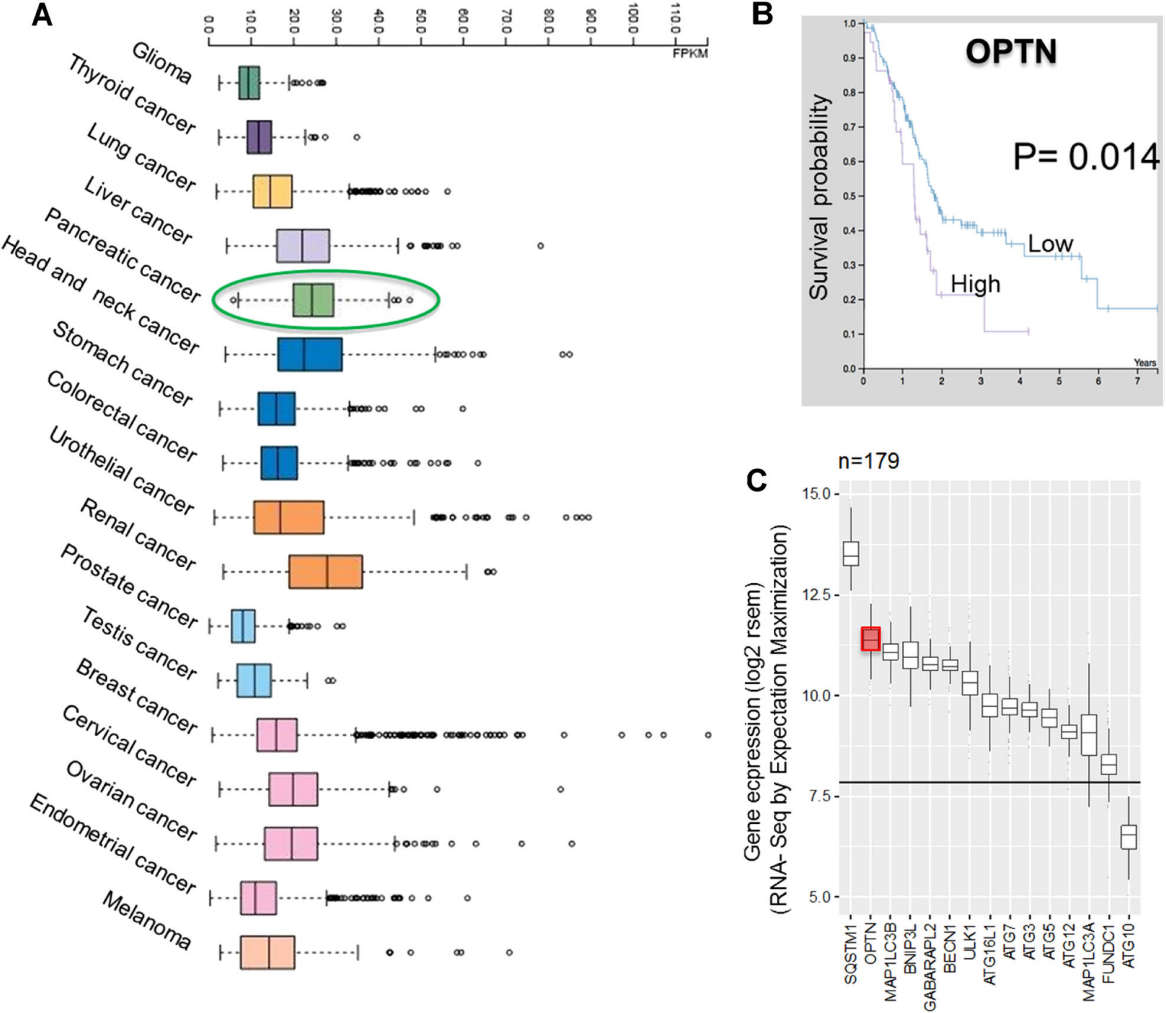
OPTN expression from publically available human cancer data. **a** Overview of the RNA-seq data in TCGA of 17 cancer cohorts showing the highest OPTN expression in renal cell carcinoma followed by PDAC (expressed in terms of median FPKM). **b** Kaplan–Meier curves of high and low OPTN expression in PDAC versus the survival of patients (log rank *p*-value (1.4e-2). Both **a** and **b** data were retrieved from human protein atlas (https://www.proteinatlas.org/ENSG00000123240-OPTN/pathology/tissue/pancreatic+cancer). **c** Expression of autophagy related genes and receptors in the PDAC cell cohort with a sample size of 179 patients. The mean control expression is indicated by the straight line across all groups. All expression levels shown are >7.5 log2 rsem (Rna-Seq by Expectation Maximization)

For assessing the expression of OPTN in human cancers, publically available databases were explored. An overview of the RNA-seq data provided by The Cancer Genome Atlas (TCGA) revealed high expression of the OPTN gene across several tumor types (Fig. 2a)^36^.

Remarkably, pancreatic cancer was associated with the second highest OPTN expression of all tumor tissues, superseded only by renal cancer, as shown in Fig. 2a^36^. The median number of fragments per kilobase of exon per million reads (FPKM) was 25 for pancreatic cancer. Increased OPTN expression was related to a significantly reduced survival probability of PDAC patients (*P* = 0.014; Fig. 2b)^37–39^.

As the reported high expression could render OPTN an attractive target for therapy, we became interested in its function. OPTN is an autophagy receptor, the importance of which is substantiated by the observation that almost all members of the autophagy process show increased expression in PDAC (Fig. 2c). As found from a TCGA cohort with a sample size of 179 PDAC patients, most of the autophagy genes show genomic expression above average, as indicated by >7.5 log2 rsem (RNA-Seq by expectation maximization). Among these genes, OPTN showed the second highest expression superseded only by sequestosome 1(SQSTM1) (Fig. 2c). Prompted by this information, we set out to discover the role of OPTN in PDAC using a panel of PDAC cell lines.

### Effect of OPTN knockdown on cellular functions

**Fig. 3:**
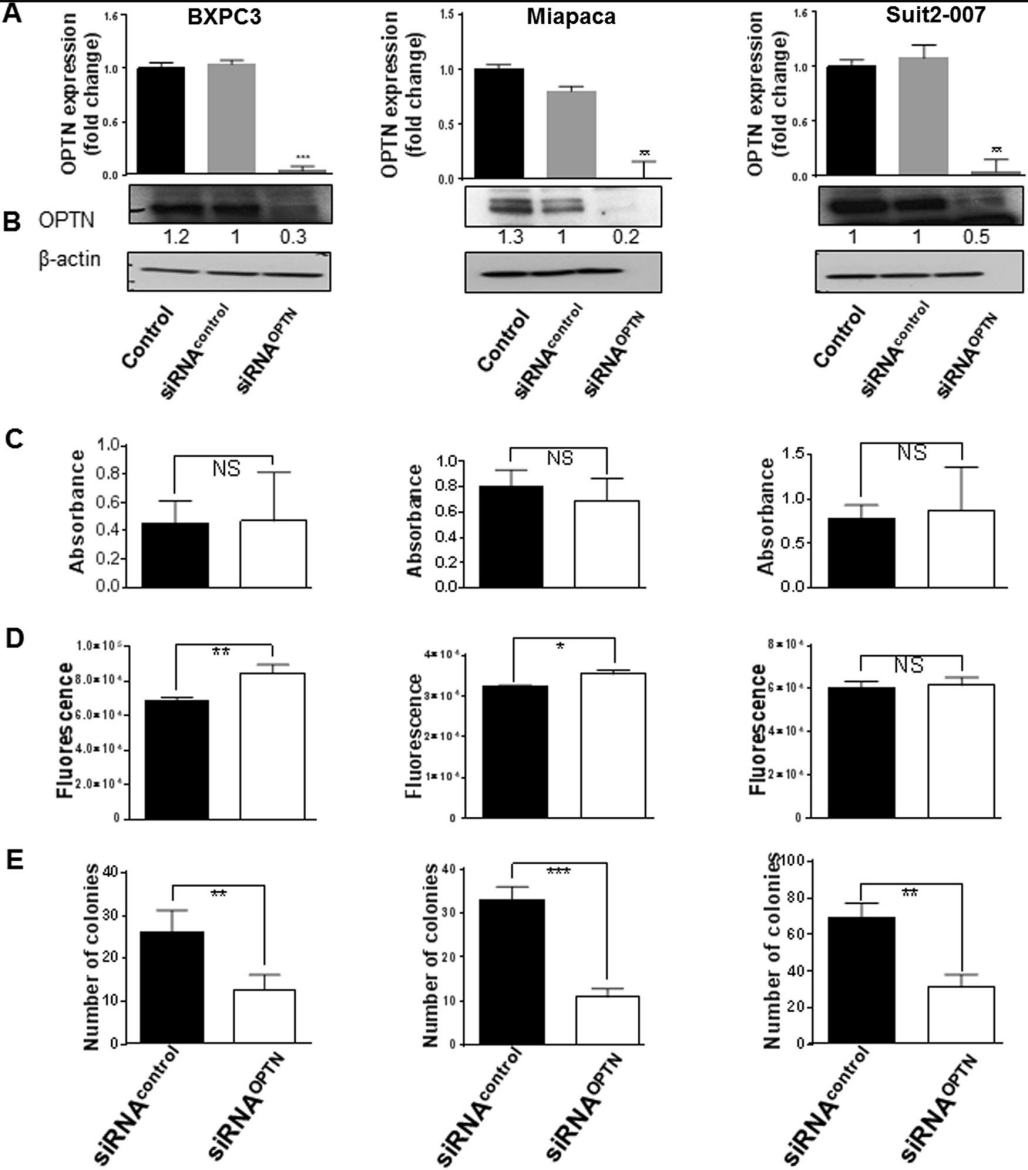
Effect of OPTN knockdown on PDAC cellular functions. The three columns represent results from BXPC3 (left), Miapaca (middle) and Suit2-007 (right). **a**, **b** mRNA and protein expression of OPTN in three pancreatic cell lines determined by qRT-PCR and WB at 48 h post siRNA transfection. All specific knockdown samples were compared with untreated and treated controls. Samples were normalized to GAPDH for qRT-PCR and to β-actin for WB. Calculation for qRT-PCR was done using the ΔΔCT method and imageJ was used to measure band densities for western blot. **c** Cytotoxic effect of OPTN silencing on three pancreatic cancer cell lines post transfection with the MTT assay. Absorbance was measured at 540 nm wavelength with a reference of 690 nm. **d** Migration assay showing the number of migrating cells following transfection using a transwell 2 compartment model that was determined by cell titer blue (Excitation 560/15 and Emission 590/ 20). The assays were done in triplicate and the results represent their average. **e** Colony formation assay was started at 48 h post transfection and shows the number of colonies as well as that of the nonspecific control after 7–10 days. The assay was done in quadruplicate and the data represent the respective average with standard deviation. Statistical analysis for all tests was done with Student’s *t*-test and one way Anova with *p* values ≤ 0.05 considered significant. *p < 0.01, **p < 0.001, ***p < 0.0001

Target specific siRNA (12.5 nM) knocked down OPTN expression successfully in three pancreatic cancer cell lines (Miapaca, Suit2-007 and BXPC3). The knockdown efficacy at mRNA level as tested by qRT-PCR (Fig. 3a), was comparably high in all three cell lines ranging from 95% (BXPC3) to >96% (Miapaca and Suit2-007).

At protein level, as determined by western blot (Fig. 3b), the knockdown efficiency ranged from 80% (Miapaca) to 70% (BXPC3) and 50% (Suit2-007). The subsequent experiments focused on the effect of OPTN knockdown on cellular functions.

As shown in Fig. 3c, proliferation was not significantly altered by OPTN knockdown in the three cell lines. In partial contrast, migration was significantly increased in BXPC3 and Miapaca cells, but not significantly in Suit2-007 cells (Fig. 3d). The most striking effect was observed for colony formation (Fig. 3e), which was reduced to 32.5, 48, and 45% in Miapaca, BXPC3 and Suit2-007 cells, respectively, in comparison with siRNA^control^ (100%).

### Modulation of genes in response to OPTN knockdown

**Fig. 4:**
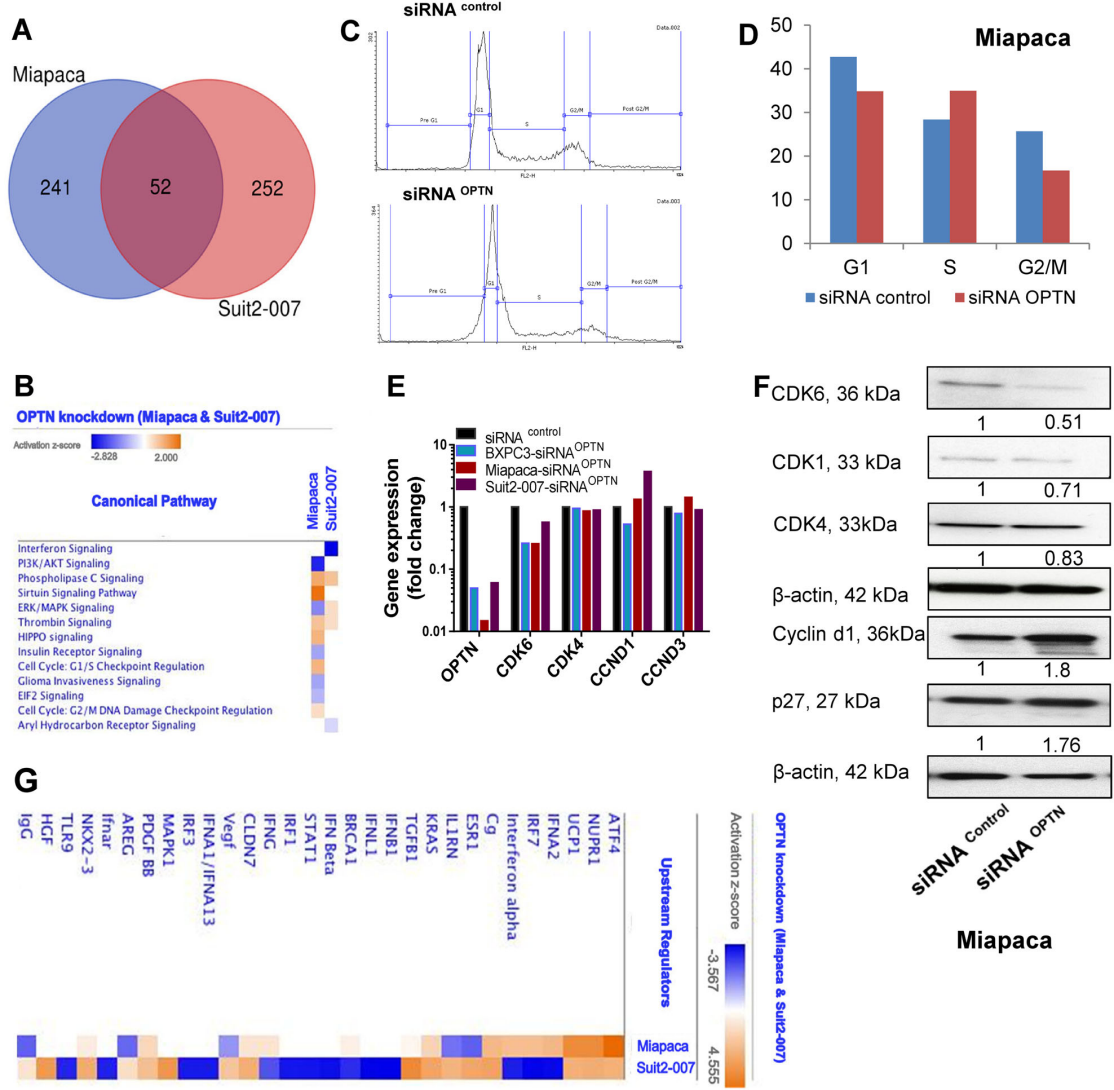
Microarray data with modulation of genes in response to OPTN-KD. **a** The Venn diagram shows 52 genes modulated in both Miapaca and Suit2-007 cells, with 1.5-fold change taken as cutoff for analysis (detailed gene lists are shown in Supplementary tables 1, 2, 3). **b** Heat map generated by IPA software showing 13 modulated canonical pathways, which are affected by the knockdown in either of the cell lines, with activation Z scores ranging from −2.8 to 2. **c** Cell cycle distribution of propidium iodide staining by flow cytometry in Miapaca cells, as calculated by flowing software. **d** The bar graph shows the distribution of Miapaca cells according to the cell cycle stages as calculated by flowing software and presented as % of cells. **e** qRT-PCR data showing the expression of cell cycle genes (CDK6, CDK4, CCND1, CCND3) following OPTN-KD in the three PDAC cell lines BXPC3, Miapaca and Suit2-007 in comparison with siRNA control as calculated by the ∆∆ CT method. **f** WB results of various CDKs in response to OPTN-KD in Miapaca cells. Analysis of the band density was done by ImageJ software and corrected to β-actin and to negative siRNA as control. **g** Heat map of the upper 30 common upstream regulators following OPTN-KD in Miapaca and Suit2-007 cells according to the activation *Z* score generated by IPA software

To possibly explain the changes in cellular functions following OPTN knockdown, a microarray was performed in two cell lines (Miapaca and Suit2-007). A cutoff (1.5-fold) was used for differentiating between significant and background changes in expression. The results were analyzed by Ingenuity Pathway Analysis (IPA) software and revealed 293 genes being significantly modulated in Miapaca compared with 304 genes in Suit2-007 cells. From these, 52 genes were significantly deregulated in both cell lines as shown by the Venn diagram (Fig. 4a). Overall 38 of these 52 genes were downregulated (e.g., CDK6: −2.38-fold in Miapaca and −1.76-fold in Suit2-007; CCNE1: −1.79-fold in Miapaca and −1.60-fold in Suit2-007; and protein arginine methyltransferase 6 (PRMT6): −2.47-fold in Miapaca and −1.76-fold in Suit2-007). In addition, 11 of 52 genes were upregulated (e.g., BCAT1: 1.7-fold in Miapaca and 1.5-fold in Suit2-007, claudin 1 (CLDN1): 1.95-fold in Miapaca and 1.85-fold in Suit2-007; and Lysosomal associated membrane protein 2 (LAMP2): 1.6-fold in both cell lines). Finally, three genes were differently modulated (e.g., interferon- induced transmembrane protein 3 (IFITM3): 1.5-fold in Miapaca and −1.713-fold in Suit2-007). All modulated genes are given in Supplementary Tables 1–3.

Canonical pathway analysis revealed the activation of phospholipase C and thrombin signaling in both cell lines. From the other 11 canonical pathways identified by IPA, the majority was altered in Miapaca cells, only (Fig. 4b).

For validating the effect on cell cycle in Miapaca cells, the DNA distribution was analyzed by flow cytometry. As shown in Fig. 4c, there were moderate reductions in cells undergoing G1 and G2/M phases, and a mild increase in S phase cells (Fig. 4c, d). The pre-G1 (subG1) fraction, as an indicator of cell death, was increased in OPTN knockdown samples with a percentage of 12.7% compared with 2.7% in the siRNA^control^ when analyzed by flowing software. These observations correlate with reduced expression of CDK6 mRNA in all three cell lines (Fig. 4e), and of CDK6 protein in Miapaca cells (Fig. 4f). Concomitantly, a less prominent reduction of CDK4 at mRNA and protein levels was observed. For cyclins, a less uniform modulation was observed, as cyclin D1 was increased in Miapaca (mRNA and protein) and Suit2-007 cells (mRNA), but decreased in BXPC3 cells (mRNA). Similarly, cyclin D3 was increased in Miapaca, but slightly decreased in Suit2-007 and BXPC3 cells (mRNA). Furthermore, p27 was increased in Miapaca cells at protein level in response to OPTN knockdown (Fig. 4f).

Analysis of upstream regulators showed matching upregulation of activating transcription factor 4 (ATF4), nuclear protein 1, uncoupling protein 1, Combgap, KRAS Proto-Oncogene-GTPase (KRAS), claudin 7, platelet derived growth factor B, and NK2 Homeobox 3. All other upstream regulators showed divergent results between the two cell lines (Fig. 4g).

### OPTN knockdown activates ER stress and chaperone-mediated autophagy

**Fig. 5:**
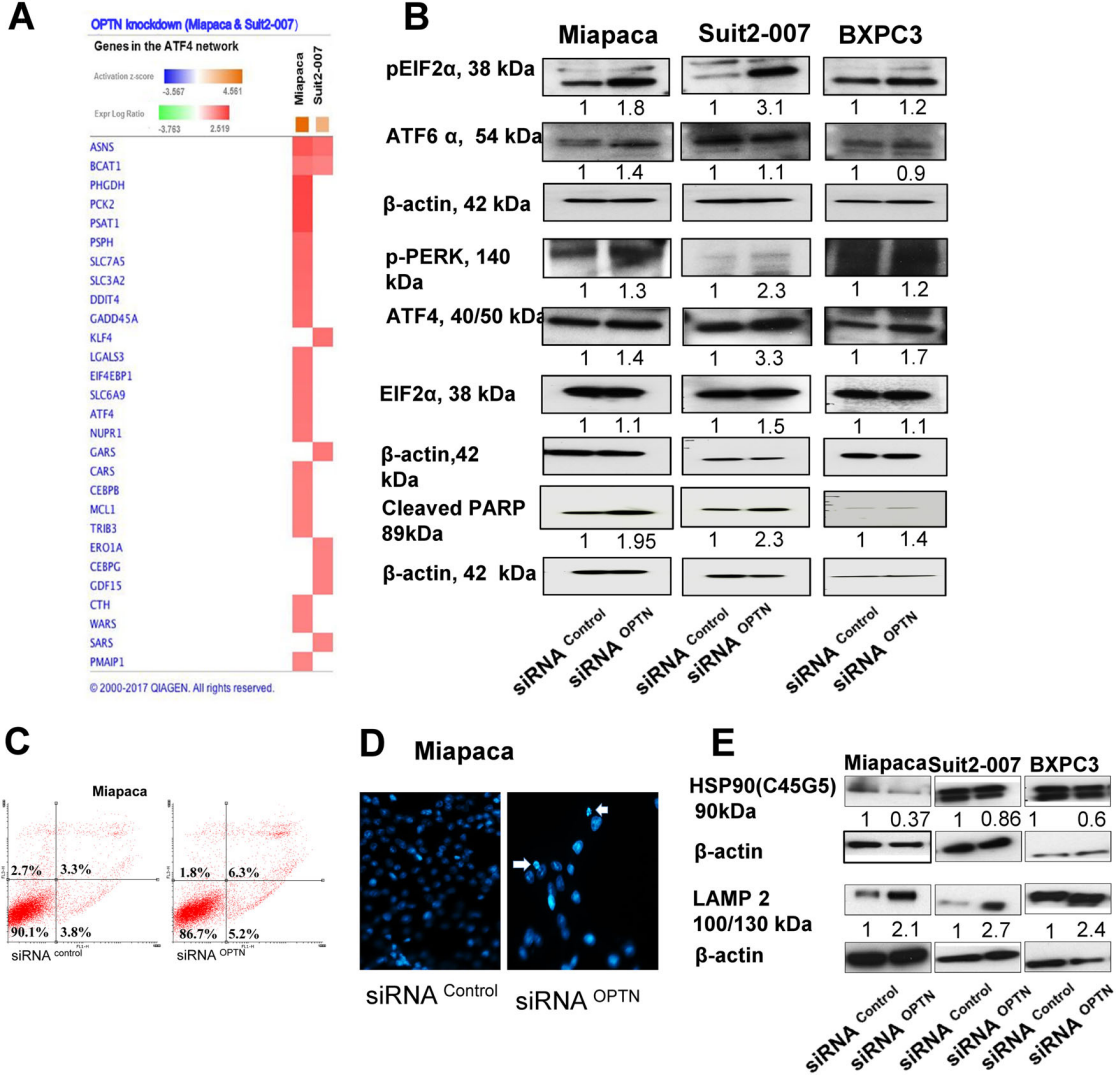
OPTN knockdown causes activation of ATF4 and other members of the ER stress-signaling pathway, chaperone-mediated autophagy and apoptosis. **a** Heat map of genes in the ATF4 network (IPA software) for Miapaca and Suit2-007 cells with the respective fold changes in expression. **b** WB analysis of the different antibodies in the ER stress-signaling pathway including pEIF2α, ATF6, pPERK and ATF4 as well as cleaved PARP. **c** Miapaca cells stained with Annexin V-FITC show increased numbers of apoptotic cells in the OPTN KD sample as compared with nonspecific control and as detected from the percentage of gated cells in the lower right quadrant. **d** Staining of Miapaca cells with Hoechst 33248 stain 48 h following transfection with siRNA. OPTN samples show nuclear abnormalities in terms of shrinkage and fragmentation. **e** Analysis of the expression of HSP90 and LAMP2 as regulators of chaperone-mediated autophagy (CMA) by immunoblotting 48 h post transfection with siRNA. Analysis of the band density was done by ImageJ software and corrected to β-actin as loading control and to negative siRNA as the main control

As ATF4 was found among the highly modulated upstream regulators of OPTN, the related network was studied more closely. This analysis showed activation of several ATF4 network genes, which, however, differed in modulation between the two cell lines, except for ASNS and branched chain amino acid transaminase 1 (BCAT1) (Fig. 5a). Interestingly, at mRNA level, ATF4 was upregulated only in Miapaca cells; however, at protein level, it was distinctly higher expressed in Suit2-007 than in Miapaca cells (3.3 vs. 1.4-fold; Fig. 5b). Other proteins of the ER stress response, as phosphorylated and unphosphorylated eIF2α and pPERK echoed this relationship, except for ATF6α, which was higher expressed in Miapaca than in Suit2-007 cells. The differential activation of the ER pathway at mRNA and protein levels was associated, however, with a highly similar activation of apoptosis, as indicated by the cleaved PARP levels (Fig. 5b). In line with PARP cleavage, staining of the Miapaca cells with Annexin V-FITC showed a higher population of early and late dead cells (Fig. 5c), while staining with Hoechst dye revealed nuclear fragmentation and shrinkage following OPTN knockdown (Fig. 5d). Staining of Suit2-007 and BXPC3 nuclei with Hoechst dye showed similar changes regarding fragmentation and shrinkage (Supplementary Fig. 1A). Annexin V-FITC staining of Suit2-007 and BXPC3 cells showed a mild increase in the number of apoptotic and necrotic cells in knockdown samples when compared with siRNA control (Supplementary Fig. 1B).

In addition, OPTN knockdown caused modulation of autophagy controlling genes. As revealed by microarray, several heat shock proteins (HSPE1, HSPA1A/HSPA1B and HSP90AB1) showed downregulation, and LAMP2, a main player in chaperone-mediated autophagy, was upregulated (Supplementary Table 2). These data were confirmed at protein level for total HSP90 and LAMP2 in all three cell lines (Fig. 5e).

For detecting autophagy in a cellular assay, acridine orange was used to visualize changes in the frequency of acidic vacuoles. However, no clear differences were observed between control and OPTN knockdown PDAC cells (Supplementary Fig. 2A, B). Furthermore, a DCFH-DA-based flow cytometric analysis was used to check for reactive oxygen species (ROS) production. In BXPC3 and Miapaca cells, this method revealed a minute increase in ROS production when comparing siRNA^OPTN^ versus siRNA^control^ exposure. However, no effect was detected in Suit2-007 cells (Supplementary Fig. 3).

### Drugs alter the expression of OPTN

**Fig. 6:**
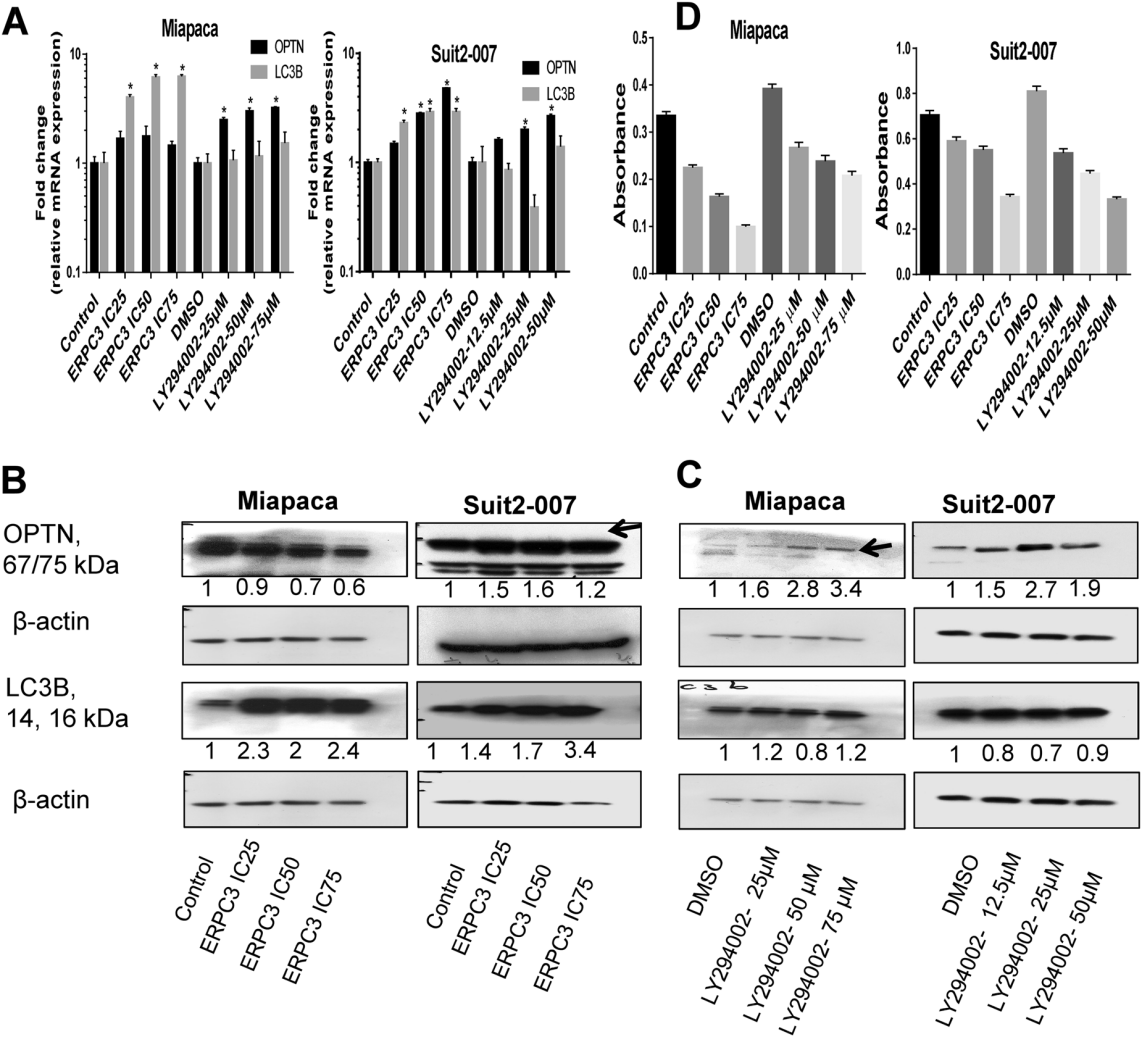
Effect of drugs on the expression of OPTN. **a** mRNA expression of OPTN and LC3B in the two pancreatic cell lines Miapaca and Suit2-007, as determined by qRT-PCR at 48 h post siRNA transfection. All specific knockdown samples were compared with corresponding controls (untreated cells as a control of erufosine and the DMSO (vehicle) treated cells in case of LY294002). Samples were normalized to GAPDH for qRT-PCR. Calculation for qRT-PCR was done using the ΔΔCT method. Statistical analysis for all tests was done by two-way Anova with significance considered for *p* values ≤ 0.05; significance is shown by an asterisk over the corresponding bar. **b** WB of Miapaca and Suit2-007 cells treated with different concentrations of the autophagy inducer erufosine 1 day following transfection with specific or nonspecific siRNA for OPTN. A total of 10–30 μg of protein were loaded per sample and the OPTN and LC3b expression levels were assessed. **c** WB of Miapaca and Suit2-007 cells treated with the autophagy inhibitor LY294002; shown are the expression of OPTN and the autophagy marker LC3B. **d** MTT assay of erufosine and LY294002 on cell survival (absorbance 540/690)

As OPTN knockdown influences genes involved in autophagy and apoptosis, two drugs were selected, which affect these cellular processes. Exposure of Miapaca and Suit2-007 cells to erufosine and LY294002, which differently influence autophagy, was followed at mRNA and protein levels as well as for their effect on proliferation. Erufosine caused concentration dependently increased mRNA transcription of OPTN, which was less prominent in Miapaca than in Suit2-007 cells. A similar increase was seen for mRNA levels of LC3B, which, however, were lower in Suit2-007 than in Miapaca cells (Fig. 6a). LY294002-induced concentration dependently increased mRNA transcription of OPTN, which was more distinct in Miapaca than in Suit2-007 cells, but LC3B mRNA levels were not significantly increased in Miapaca and even decreased in Suit2-007 cells (Fig. 6a). At protein level, erufosine caused a 40% reduction of OPTN levels in Miapaca but a 1.6-fold increase in Suit2-007 cells. Concomitantly, the concentrations of LC3B were increased in both cell lines (Fig. 6b). LY294002 caused a uniform increase of OPTN in both cell lines, but no effect (Miapaca) and a slight reduction (Suit2-007) in LC3B levels (Fig. 6c). Both drugs caused an antiproliferative effect in the PDAC cell lines. The effect of erufosine was more pronounced in Miapaca than in Suit2-007 cells; whereas that of LY294002 was higher in Suit2-007 than in Miapaca cells (Fig. 6d). In combination with OPTN knockdown, however, erufosine exposure of Miapaca and Suit2-007 cells caused no additional decrease in OPTN mRNA or protein levels compared with OPTN knockdown alone (Supplementary Fig. 4A, B). Equivalent results were observed for the combination of OPTN knockdown and exposure to LY294002 (Supplementary Fig. 4A, C). For LC3B, there was no significantly increased concentration at mRNA and protein levels when OPTN knockdown was combined with either erufosine or LY294002 (Supplementary Fig. 4A, D).

The observed additive interaction effects were confirmed by respective studies on the proliferation of Miapaca and Suit2-007 cells in response to erufosine, LY294002, or knockdown of OPTN alone, or combination of OPTN knockdown with either erufosine or LY294002 exposure (Supplementary Fig. 4E).

## Discussion

Physiologically, OPTN has a function as autophagy receptor and its presence stimulates autophagy^4^. In addition, OPTN is known for its role in neurodegenerative diseases as amyotrophic lateral sclerosis and open angle glaucoma^23^. Interestingly, autophagy plays a role in neurodegenerative diseases as well as in cancer^40^. Based on publically available data we found that OPTN is highly expressed in cohort studies of 17 cancer types, with the highest expression in renal followed by pancreatic cancer, and the lowest expression detected in prostate cancer^36^. In addition, when mining TCGA, OPTN was the second most expressed gene in PDAC from all autophagy related genes. Thus, we investigated the role of OPTN in PDAC, a cancer with almost identical incidence and mortality rates.

In this first study on the role of OPTN in experimental pancreatic cancer, it was tempting to reduce OPTN levels, which are generally increased in PDAC, by a knockdown strategy in order to investigate its value as therapeutic target. Following successful knockdown in three PDAC cell lines, changes in cancer growth related cellular properties were recorded. They were used as surrogate for a response of PDAC in vivo. In addition, changes in gene expression caused by OPTN knockdown were recorded by a microarray.

The knockout of other autophagy genes as ATG5 was associated with increased proliferation, migration and colony formation in mouse embryonic fibroblast and NIH 3T3 cells^41,42^. In contrast, knockdown of OPTN had a minor and nonsignificant effect on the proliferation of pancreatic cancer cells, caused slightly but significantly increased migration in two of three PDAC cell lines and decreased colony formation distinctly in all three pancreatic cancer cell lines when compared with nonspecific siRNA control.

In order to understand the mechanisms that govern these alterations, a microarray was performed in two PDAC cell lines after transient OPTN knockdown to obtain a comprehensive analysis of related mRNA expression changes. With regard to genes, which were modulated significantly in both cell lines (*n* = 52), 11 genes were found to be related to cell movement and motility. In addition, 2 of these 11 genes were related also to colony formation. From these 11 genes, 4 are known for their relationship to cancer progression: they include BCAT1, which was more than 1.5-fold upregulated in both cell lines. Interestingly, knockdown of BCAT1 was associated with reduced migration of nasopharyngeal and ovarian cancer cells^43,44^. Another gene was CLDN1, which was upregulated more than 1.8-fold in both cell lines. CLDN1 is a member of the tight junction family of genes and has attracted attention because of its altered expression in many cancers as well as its association with cellular properties (invasion, migration) indicating cancer progression, which are normalized upon its knockdown in breast and lung cancer cells^45,46^. Further, PRMT6 was downregulated more than 1.7-fold in both cell lines. PRMT6 knockdown reduced the number of colonies in U2OS osteosarcoma cells^47^ as well as in ZR75 and MCF7 breast cancer cells overexpressing proline-, glutamic acid-, and leucine-rich protein 1^48^. Moreover, PRMT6 was found to regulate embryonic stem cell identity by modulating pluripotency genes and inducing expression of differentiation markers^49^. In contrast, overexpression of PRMT6 was associated with reduced colony formation in breast and prostate cancer cells^50^. Finally, IFITM3 was 1.5-fold upregulated in Miapaca and 1.7-fold downregulated in Suit2-007 cells. IFITM3 is known as part of the cellular defense against influenza infection, but also plays a role in cancer progression as its knockdown suppressed migration and invasion in gastric cancer cells^51^.

**Fig. 7:**
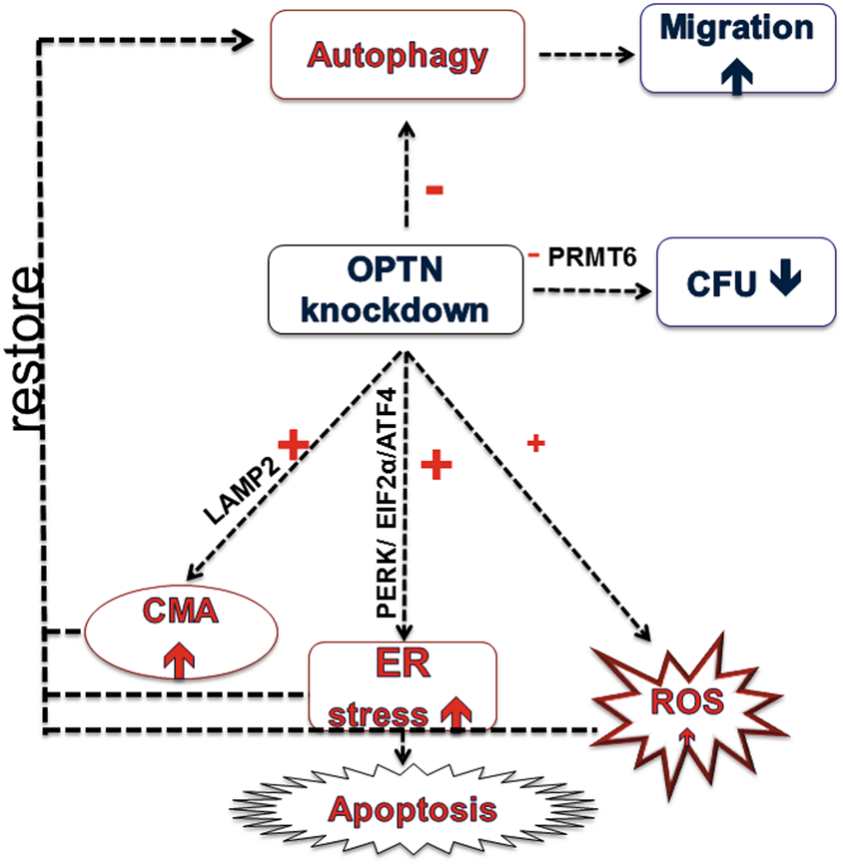
Scheme of the response of PDAC cells following OPTN KD. OPTN knockdown triggered inhibition of autophagy and caused increased migration and reduced colony formation. PDAC cells tried to reverse autophagy inhibition by activating different pathways; i.e., by ER stress as indicated by activation of the PERK/EIF2-α /ATF4 pathway, by stimulating CMA as manifested by upregulation of LAMP2, and by a low increase in ROS production. Cells which could not reverse the consequences of OPTN knockdown underwent apoptosis

In addition to these functions influencing cell behavior, the microarray on PDAC cells with OPTN knockdown revealed induction of ER stress and chaperone-mediated autophagy. The activation of these cellular systems could be interpreted as adaptive mechanisms elicited in response to OPTN knockdown. In this perception (see Fig. 7), OPTN knockdown causes inhibition of autophagy. The PDAC cells, however, try to overcome this situation by releasing ER stress, as indicated by increased ATF4 levels and related signaling molecules including pPERK, (p) eIF2α and ATF6. The central role of ATF4 in ER stress and in inducing autophagy in response to hypoxic conditions has been well documented^52–54^. ROS contribute to autophagy induction^55^.

Further, heat shock proteins HSP90AB1, HSPA1A/HSPA1B, HSPE1 were downregulated following knockdown of OPTN. HSP90 has a regulatory role in the chaperone complex transferring damaged and misfolded proteins to the lysosome for chaperone-mediated autophagic (CMA) degradation. CMA is another form of autophagy, where proteins are transported directly from cytosol to the lysosomal lumen via interactions with chaperones located in their membrane and via a protein translocation complex^56^. The concomitant reduction of HSP70 levels presumably further weakens the chaperone complex, which plays a central role through its capability to recognize the amino acid consensus sequence of misfolded and damaged proteins^56^. LAMP2 was twofold upregulated following OPTN knockdown. This protein is the docking station for HSP70 and imports proteins tagged for degradation into the lysosomes. It is conceivable that the twofold increased expression at mRNA and protein levels was triggered by the diminished chaperone complex. In addition, ER stress may be responsible for LAMP2 induction^57^. Apart from the role of HSP90 in CMA, specific inhibitors of HSP90, as geldanamycin, may induce autophagy^58^.

Apoptosis was found to be upregulated in all cell lines in response to OPTN knockdown as proven by increased cleaved PARP expression, fragmentation of the nuclei by DAPI staining and increased Annexin V binding in respective pancreatic cancer cells. In this context, autophagy inhibition was found to induce apoptosis through P53 and ER stress^59^. Li et al. demonstrated that withaferin A, a biologically active extract, induced ER stress mediated apoptosis through inhibition of lysosomal autophagic and proteosomal degradation^60^. Another study revealed a switch that decreases apoptosis in favor of protective autophagy in methotrexate-resistant choriocarcinoma cells. This change was indicated by ER stress through the PERK/ATF4 pathway, or by ROS production through JNK/p62 signals^61^, which are both capable to induce autophagy.

Owing to these rescue mechanisms that counteract autophagic inhibition and restore autophagy back to basal levels, we did not find many cellular effects that could be related to autophagy. Any of these mild effects found in response to OPTN knockdown were more pronounced in Miapaca than in Suit2-007 cells. The sub lying reason could be that Suit2-007 cells have undergone more mutations than Miapaca cells and therefore show less dependency on internal signaling. The effect on different pancreatic cellular functions following OPTN-KD was mild. The most important changes following knockdown are summarized in Fig. 7.

Contrasting with the mild changes related to autophagy, the cell cycle was clearly changed in Miapaca cells in response to OPTN knockdown. In line with the altered G1/S and G2/M checkpoint regulation described by our IPA analysis, there were increased levels of p27, which in conjunction with reduced CDK6 levels caused an increase in non-phosphorylated cyclin D1. Concomitantly, there were reduced percentages of G2/M and post G2/M phases, as the cell cycle did not move further after reaching the S phase. In this scenario, the reduction observed for the G1 phase corresponds to the increase seen for the Pre-G1 phase as these cells probably underwent apoptosis.

Although OPTN is part of the autophagy system, autophagy was only mildly affected in response to OPTN knockdown. This was surprising because a decreased OPTN level was expected to be in imbalance with cellular LC3 levels, for which OPTN has a binding site (Fig. 1c, d). We thus speculate that other autophagy receptors as e.g., SQSTM1/p62 took over and replaced the function of OPTN. Therefore, two drugs were used, which modulate autophagy in a reverse manner.

LY294002 is a well-known inhibitor of phosphatidylinositol 3-kinase (PI3K) that acts on the ATP binding site of the enzyme^62^. It was chosen as PI3K is required for autophagy and inhibition of PI3K with LY294002 can inhibit autophagic sequestration^63^. Erufosine, on the other hand, is a potent inhibitor of mTOR and inducer of LC3B^64,65^. As expected, erufosine increased the expression of LC3B in both cell lines, whereas LY294002 caused no or a depressive effect on this indicator of autophagy^66^. However, both drugs were largely ineffective in reversing the knockdown caused by siRNA against OPTN. Further, we had anticipated that the upregulation of LC3B by erufosine would enlarge the imbalance between OPTN and LC3B. However, the erufosine effect on LC3B levels was not antagonized by OPTN knockdown, indicating that the two autophagy proteins are regulated differently. Taken together, the influence of the two drugs on LC3B was not paralleled by their influence on OPTN and in combination with OPTN knockdown, the two drugs caused additive interactions; hence it is plausible that the two drugs and OPTN knockdown serve different pathways.

The autophagy receptor OPTN is highly expressed in many human cancers including pancreatic cancer. Its knockdown in a panel of PDAC cells was related to mildly increased migration and distinctly reduced colony formation. In response to its knockdown, different rescue mechanisms became active; namely ER stress response and chaperone-mediated autophagy. These mechanisms reduce the potential damage caused by the gene’s knockdown. Finally, OPTN knockdown probably influences pathways different from those of erufosine and LY294002 in modulating autophagy.

## Materials and methods

### Cell lines and cell culture condition

Three human pancreatic adenocarcinoma cell lines were cultured in RPMI-1640 (BXPC3, Suit2-007) and DMEM (Miapaca) medium (Invitrogen and Sigma Aldrich, Germany respectively). All media were supplemented with 10% fetal bovine serum and L-glutamine (2 mM), penicillin (100 IU/ml) and streptomycin (100 μg/ml) (Invitrogen, Karlsruhe, Germany). The cells were passaged routinely using 0.25% Trypsin/EDTA solution and maintained under standard incubation conditions (5% CO_2_, 37 °C) in a humidified atmosphere. Cell lines were authenticated using Multiplex Cell Authentication by Multiplexion (Heidelberg, Germany) as described recently^67^. The SNP profiles matched known profiles or were unique.

### Small interfering RNA (siRNA) experiments

Human OPTN siRNA (siRNA^OPTN^) was purchased (Invitrogen, Germany). siRNA seq (5′- 3′) sense: GGA AGU UUA CUG UUC UGA U (dT) (dT); antisense: AUC AGA ACA GUA AAC UUC C (dT) (dT)). AllStars siRNA serving as a non-targeting siRNA control was purchased from Qiagen, Germany. Cells were seeded at pre-optimized cell density in a six-well plate (8–10 × 10^4^/2000 μl), 24 h later siRNA was added in OptiMEM media to a final concentration of 12.5 nM using lipofectamine RNAimax (Invitrogen, Germany) as a transfection agent. Cells were incubated for 48 h under standard conditions until start of subsequent experiments.

### Cell viability assay

Viability of selected cell lines, following knockdown by siRNA^OPTN^ and siRNA^control^, or after treatment with erufosine (gift from Prof. H. Eibl, Max Planck-Institute of Biophysical Chemistry, Goettingen, Germany) or LY294002 (Cayman Chemical, USA) was tested by the MTT (3-[4,5-dimethylthiazol-2-yl]-2,5 diphenyltetrazolium bromide) assay as described before^68^.

### Migration assay

The migratory behavior of pretreated pancreatic cancer cells by siRNA was studied by seeding cells in a 2 compartment model separated by 8 μm pore size polycarbonate membranes (Millicell, Millipore, Schwalbach, Germany) in a 24-well plate.

The bottom layer of the plate consisted of 700-μl media with 20% FCS. Then, tumor cells (0.5–1 × 10^5^ cells/300 μl optiMEM media) were seeded in the upper compartment. After 48 h, cell titer blue (Promega Gmbh, Mannheim, Germany) was added into the lower compartment (140 µl) at a ratio of 1/5 of the total amount of media contained. Fluorescence emitted by migrating viable cells due to their ability to reduce the dye Resazurin into the fluorescent pink dye Resorufin was estimated using a fluorometer at wavelengths (560/20) for excitation and 590/10) for emission.

### Colony formation assay (CFA)

The cells were treated for 48 h, harvested and counted. Cells were then transferred into semi-solid medium containing 0.8% methylcellulose (MC) and 40% FCS, and plated onto 24-well plates (0.4 ml/well, with a final number of 100, 250, and 500 cells for Suit2-007, Miapaca, and BXPC3, respectively). These were incubated under usual cell culture conditions (37 °C, 5% CO2 in humidified air) and colonies were counted under the microscope after 7–10 days. Clusters of ≥10 cells were counted as colony. Data were represented as a percentage of the untreated control colony forming units (CFU).

### Cell cycle assay

The distribution of DNA following knockdown was assessed using Propidium iodide staining and flow cytometry. Shortly, treated cells were harvested and resuspended in 0.1 ml of PBS (≈2 × 10^5^ cells) after 48 h followed by the addition of 1.4 ml ice-cold ethanol (70%)/sample for fixation. The cells were incubated for 2 h at 4 °C, then washed, resuspended in PBS containing RNase A (1 mg/ml) for digesting any contaminating RNA and incubated for 30 min at 37 °C. Subsequently, PI (50 µg/ml) was added to the cells, and analysis was done immediately using a FACS Calibur (BD Biosciences, San Jose, CA, USA). Ten thousand cells (events) were analyzed from each sample, and the cell’s distributions in G0/G1, S and G2/M phases of cell cycle were calculated by flowing software.

### RNA isolation and cDNA synthesis

After 48 h of exposure, the treated cells were harvested and washed; total RNA was extracted from the cell pellets with RNeasy Mini Kit (Qiagen, Hilden, Germany) following the manufacturer’s protocol. Concentrations of the extracted RNA were measured using a Nanodrop spectrophotometer (Nanodrop Technologies, Germany), and 1000 ng RNA was used to synthesize complementary DNA (cDNA) by using reverse transcriptase (Revertaid enzyme, Thermo Scientific, Schwerte, Germany). The cDNA samples were used as a template for further real-time PCR amplification and analysis.

### Quantitative real-time RT-PCR

Modulation of OPTN expression (for primers and probes used, see Supplemantary Table 4) following gene knockdown with siRNA was investigated by qRT-PCR. Briefly, 5 μl of LC480 2X Master Mix (Roche, Mannheim, Germany) was used in the amplification procedure of the cDNA sample, with 0.2 μl of each of the primers, 0.1 μl of the appropriate probe from the Human Universal Probe library (Roche, Mannheim, Germany) and 2.5 μl of water per reaction and 2 μl of the cDNA. The mixture was added into 384-well plates with a total volume of 10 μl/well. Each sample was represented in triplicate. GAPDH was used as reference gene in the normalization process of the corresponding sample. Corresponding changes in the expression levels of selected genes were calculated by 2^ΔΔCT^ method.

### Western blot analysis

At 48 h following treatment of pancreatic cancer cells by siRNA, erufosine or LY294002, the cells were harvested from six-well plates and washed with PBS. The cell pellets were thoroughly suspended in an adequate volume of lysis buffer consisting of RIPA buffer, complete protease inhibitor cocktail tablets (Roche, Mannheim, Germany), phosphostop tablets, and sodium vanadate. The samples were kept under constant agitation for 30 min at 4 °C, then centrifuged for 20 min (14000 rpm, 4 °C). Afterward, the supernatants were collected and the protein concentration was estimated using the Pierce Protein Assay. The calculated total protein lysates (20–30 μg) were added together with a DTT detergent and 4X dye to be denatured for 5 min (99 °C, 350 rpm) and thereafter were subjected to electrophoresis process using a 4–12% gradient polyacrylamide SDS gels in a 1X Laemmli running buffer. Transfer of proteins to PVDF membranes was done using a wet blotting system. The membranes were subject of test for several proteins using specific primary antibodies: OPTN (c2) (sc-166576, G2315), OPTN (D2L8S) (cs- 58981 S), ATF4 (sc-2280), ATF6 (sc-22799), cdk6 (cs-3136), cdk4 (cs-12790), cdk1 (Ab-133327), EIF2-α (cs-9722), pEIF2-α (cs-9721), pPERK(sc-32577), LAMP2 (cs-49067S), LC3B (cs-2775S), HSP90 (C45G5) (cs-4877), P27 kip (cs-2552), Cyclin D1 (cs-2978), cleaved PARP (cs-9541s), β-actin; mouse monoclonal antibody (sc-47778), and β-actin; goat polyclonal antibody (sc-1615). A corresponding secondary antibody was added and then the immunoblots were developed using an ECL-system (Amersham Pharmacia Biotech, Munich, Germany). Relative concentrations to β-actin, which was used as the loading control for whole cell lysates, were evaluated by densitometric analysis of images using the ImageJ software.

### Microarray analysis

Total RNA was eluted in Rnase free water. The microarray was performed by the genomics and proteomics core facility in the DKFZ using Illumina Human HT-12 v4 Expression BeadChip Kits. Analysis of omic-data was done using the IPA software after the application of a cutoff of 1.5-fold change with comparison of knockdown samples with siRNA negative control.

### Acridine orange (AO) staining

Acidic and autophagic vacuoles emit red fluorescence when stained with AO stain. Briefly, 0.8–1 × 105 cells were seeded in a six-well plate and treated with siRNA for 48 h. Cells were then allowed to stay for 15 min in serum-free medium containing 1 μg/ml 3,6-bis(dimethylamine) acridine orange and then observed with a Zeiss cell observer microscope. The excitation filter used was 488 nm and the emission filter was 505–530 nm and >650 nm.

### Hoechst 33342 staining

Following transfection, study the nuclear integrity was determined by using fluorescent Hoechst stains. Cells in a density of 0.8–1 × 10^5^ cells were seeded on sterilized cover slips in six-well plates and allowed to attach and grow under standard incubation conditions. This was followed by siRNA treatment 24 h later. After 48 h, the cells were washed once with PBS and fixed by 4% paraformaldehyde for 10 min. Later, the cells were permeabilized with 0.3% Triton X-100 in 1 ml PBS for 10 min, stained with Hoechst 33342 solution, and kept in a dark environment for 10 min. The cells, grown on cover slips, were placed top-down on glass slides and photographed with a fluorescence microscope (350 nm excitation wavelength).

### Reactive oxygen species measurement

2′,7′–Dichlorofluorescin diacetate (DCFH2-DA) is a cell permeable fluorogenic dye that measures ROS activity within the cell. In short, 0.8–1 × 10^5^ cells were seeded in a six-well plate and treated with siRNA for 48 h. A working solution of 10 μM DCFH2-DA was prepared in serum-free media. Cells are washed twice in PBS and incubated for 30 min in the dark with DCFH2-DA. The cells were then washed, harvested and cell pellets suspended in 1 ml PBS. All samples as well as negative and positive controls were subjected to ROS detection using the 488 nm laser for excitation and 535 nm for detection with a BD Accuri C6 flow cytometer.

### Annexin V assay

The Annexin V-FITC Kit (Biolegend, 640914) and rh Annexin V/APC (Invitrogen, BMS306APC-100) allow detection of apoptotic cells through their affinity to bind to phosphatidylserine residues, which become exposed upon apoptosis and then allow their detection and quantitative determination by fluorescent flow cytometry. The included PI permits labeling of cellular DNA, which is a feature of necrosis when the cellular membrane has been totally compromised. The combined use of both reagents allows the differentiation between three cell populations; i.e., early apoptotic, necrotic, and viable cell populations in response to treatment. Briefly, cells were seeded in a six-well plate, treated by siRNA and harvested following 48 h with EDTA free trypsin (0.25%), then washed once with PBS as well as with 1X binding buffer. The cells were counted and 2*10^5^ cells were re-suspended in 100 μl of the 1X binding buffer containing 5 μl Annexin V-FITC per sample. The cells were incubated for 15 min at room temperature in the dark, followed by washing with 1X binding buffer to remove the unbound Annexin V-FITC, and centrifuged. The cell pellets were resuspended in 200 μl of the 1X binding buffer and 5 µl of propidium iodide/sample (provided with the kit) per sample. Analyses were done using flowing software.

### Statistical analysis

Student’s *t*-test (2 groups) was used to estimate the significance of the group of interest in comparison with a control group for either viability of the cells, colony formation inhibition or increased migration in the siRNA- versus nonsense-treated cells. Graphs and calculations were created using the Graphpad prism version-6 software. *P*-values below 0.05 were considered statistically significant.

## Supplementary information

Supp. Figure 1

Supp. Figure 2

Supp. Figure 3

Supp. Figure 4

Supp. Table 1

Supp. Table 2

Supp. Table 3

Supp. Table 4

WB1

WB56

WB57

WB58

WB59

WB60

WB61

WB62

WB63

WB2

WB3

WB4

WB5

WB6

WB7

WB8

WB9

WB10

WB11

WB13

WB14

WB15

WB16

WB18

WB19

WB20

WB21

WB22

WB23

WB24

WB25

WB26

WB27

WB28

WB29

WB30

WB31

WB32

WB35

WB36

WB37

WB38

WB39

WB40

WB41

WB42

WB43

WB44

WB45

WB46

WB48

WB49

WB51

WB52

WB53

WB54

WB55
