# Supplementary material for: Optineurin downregulation induces endoplasmic reticulum stress, chaperone-mediated autophagy, and apoptosis in pancreatic cancer cells
Source: Cell Death Discov. 2019 Aug 9;5:128. doi: 10.1038/s41420-019-0206-2 (PMC6689035; doi:10.1038/s41420-019-0206-2)
Supplement: Supplementary file 1 — Supp. Figure 1 [file 41420_2019_206_MOESM1_ESM.pdf]

Supplementary Figure 1

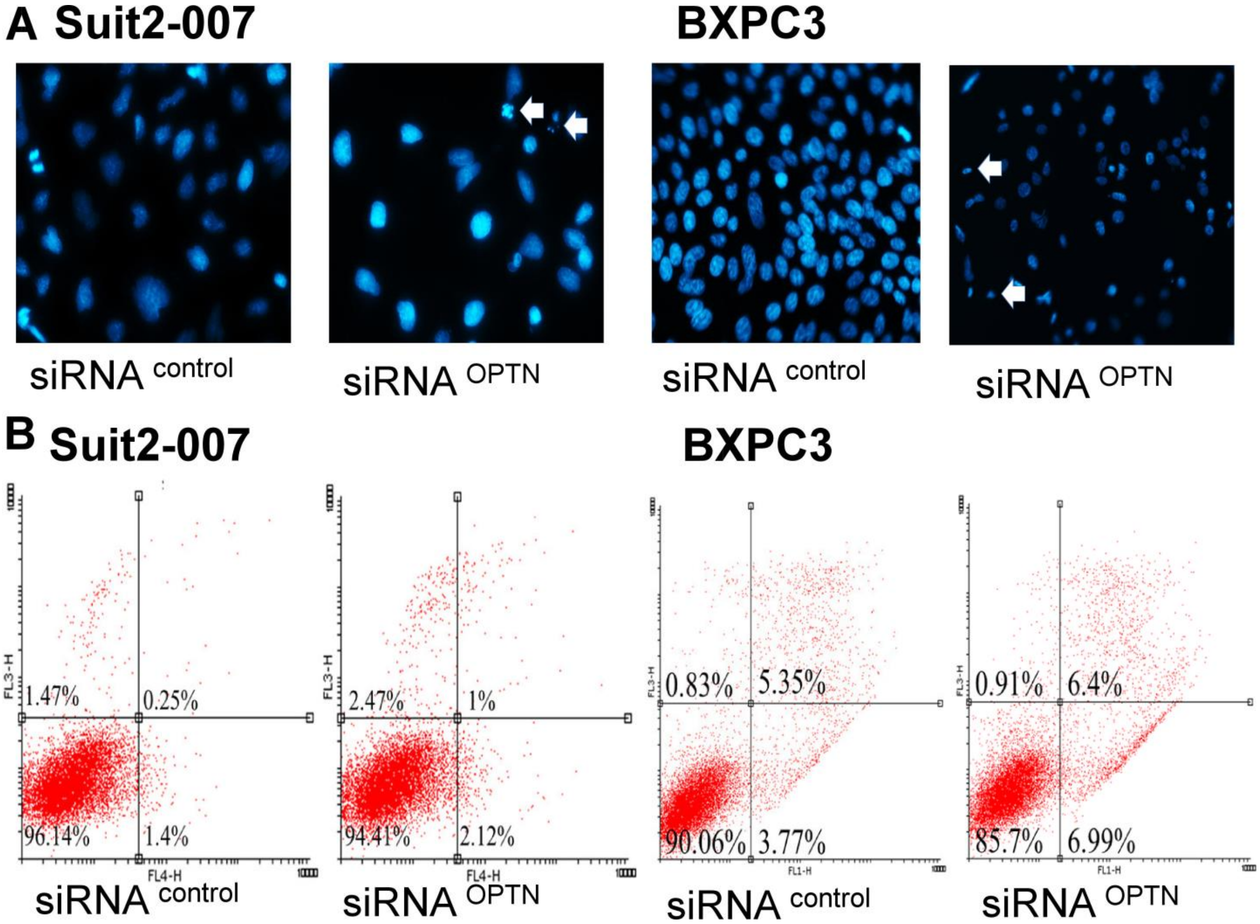

Supplementary Figure 1: Apoptotic changes of PDAC cells following OPTN knockdown

A.: Suit2-007 and BXPC3 pancreatic cancer cells were stained with Hoechst 33248 at 48h after transfection with siRNA. They were analyzed by a motorized inverted Zeiss cell observer microscope using an excitation wavelength of 350 nm. OPTN samples show nuclear abnormalities in terms of shrinkage and fragmentation. **B.:** Annexin V-FITC stain of PDAC cells at 48h post siRNA transfection. Suit2-007 and BXPC3 cells were stained with Annexin –APC and analyzed by flow cytometry (channel 4). They showed only a mild increase in the number of apoptotic and necrotic cells following OPTN KD when compared to siRNA control. The channels were chosen to define early apoptotic events (lower right), late apoptotic and necrotic events (upper right and left). Numbers in the quadrants correspond to the percentage of gated cells detected. OPTN KD showed increased percentage of apoptotic and necrotic cells when compared to the corresponding non-specific siRNA control.
