# Supplementary material for: Optineurin downregulation induces endoplasmic reticulum stress, chaperone-mediated autophagy, and apoptosis in pancreatic cancer cells
Source: Cell Death Discov. 2019 Aug 9;5:128. doi: 10.1038/s41420-019-0206-2 (PMC6689035; doi:10.1038/s41420-019-0206-2)
Supplement: Supplementary file 2 — Supp. Figure 2 [file 41420_2019_206_MOESM2_ESM.pdf]

Supplementary Figure 2

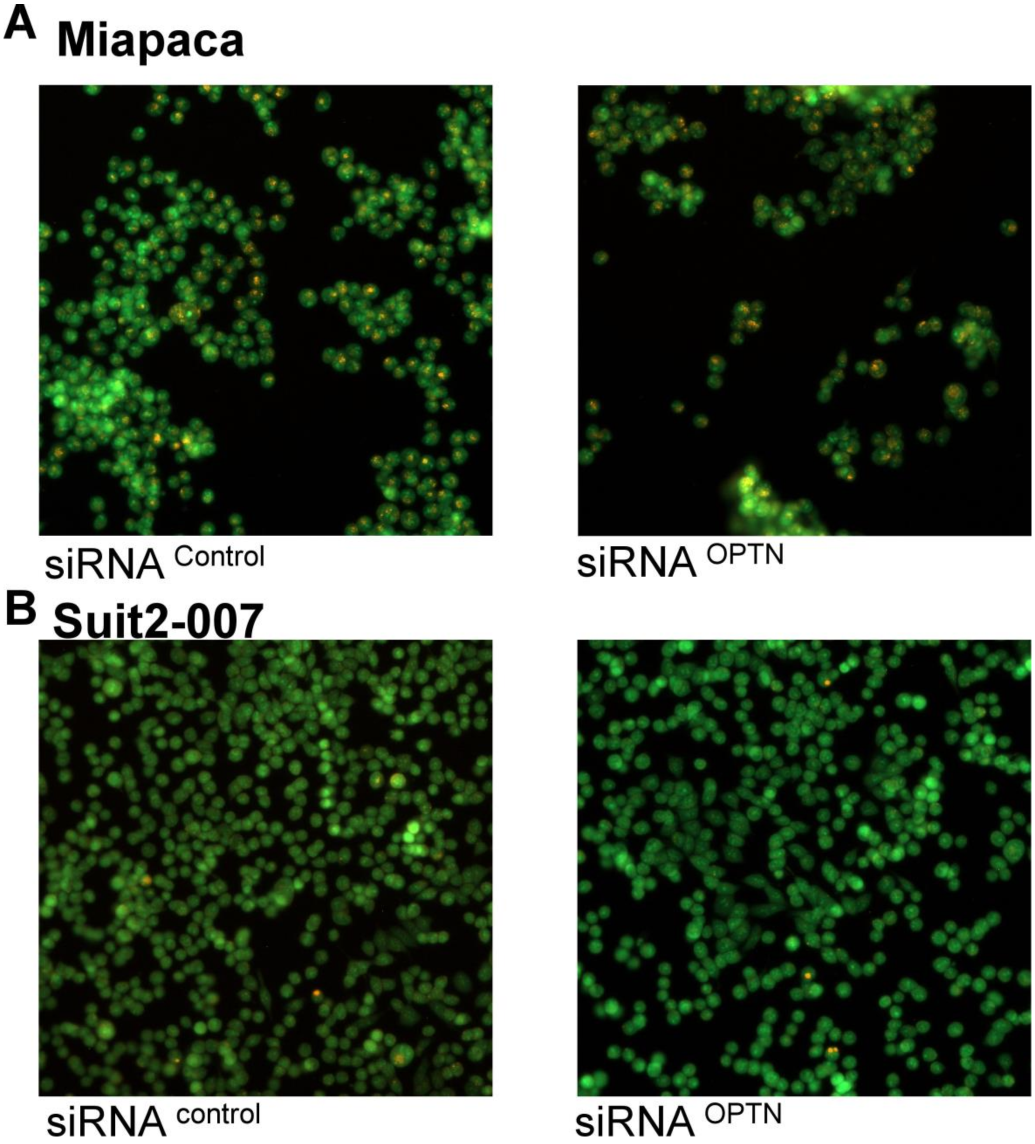

Supplementary Figure 2: Acridine orange staining of acidic vacuoles indicates the presence of autophagosomes for both, nonspecific control and OPTN-KD samples from Miapaca and Suit2-007 PDAC cells. The acidic vacuoles stained orange following exposure to acridine orange stain (excitation 488/ emission 505-530).
