# Supplementary material for: Optineurin downregulation induces endoplasmic reticulum stress, chaperone-mediated autophagy, and apoptosis in pancreatic cancer cells
Source: Cell Death Discov. 2019 Aug 9;5:128. doi: 10.1038/s41420-019-0206-2 (PMC6689035; doi:10.1038/s41420-019-0206-2)
Supplement: Supplementary file 3 — Supp. Figure 3 [file 41420_2019_206_MOESM3_ESM.pdf]

Supplementary Figure 3

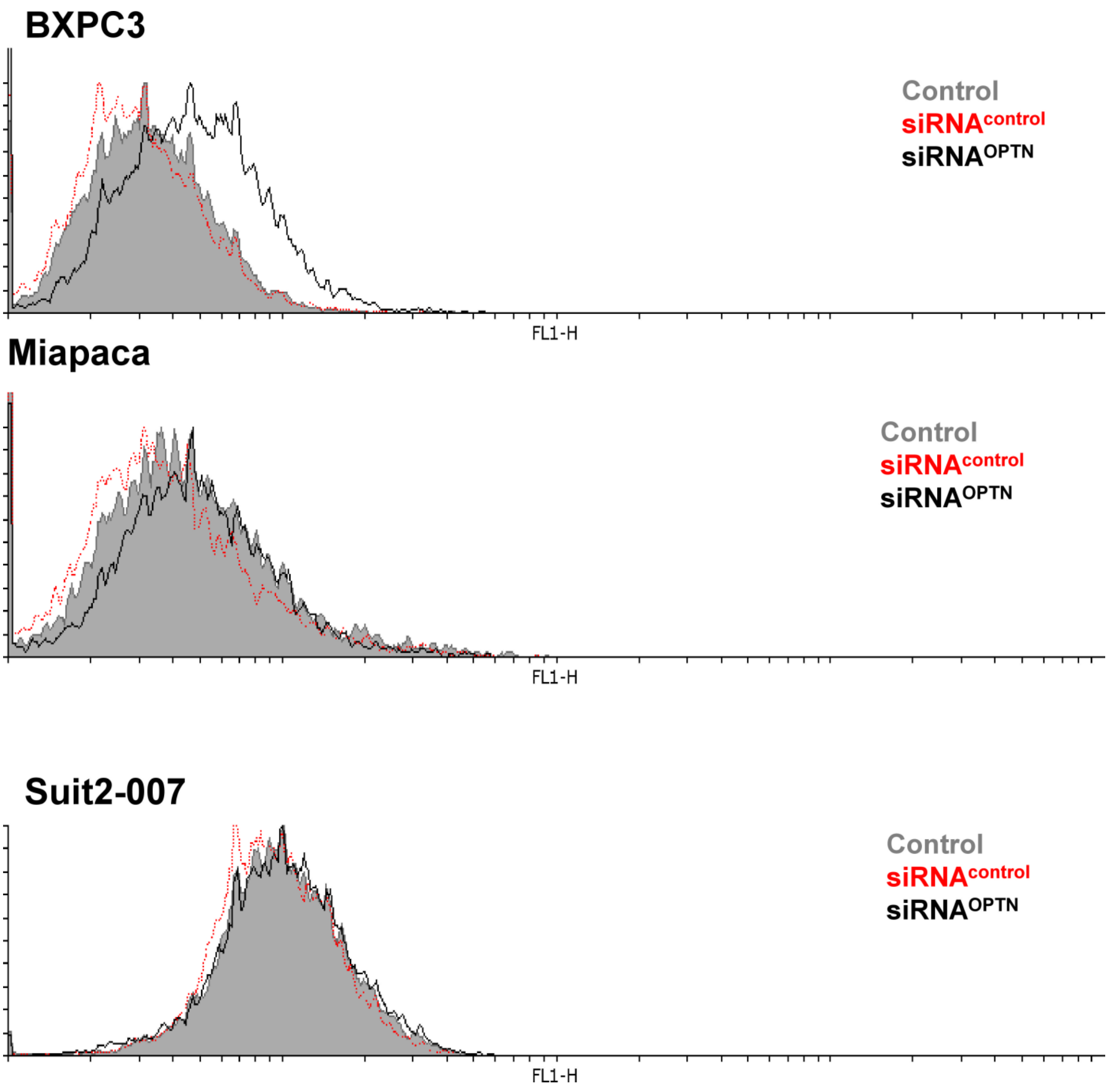

Supplementary Figure 3: ROS measurement by flow cytometry using DCFH-DA post OPTN knockdown. ROS production is indicated by a shift towards right in the fluorescence signal. OPTN knockdown in BXPC3 and Miapaca cells showed an increase in the production of ROS when compared to the siRNA control, which was not clearly detectable in Suit2-007 cells.
