# Supplementary material for: Optineurin downregulation induces endoplasmic reticulum stress, chaperone-mediated autophagy, and apoptosis in pancreatic cancer cells
Source: Cell Death Discov. 2019 Aug 9;5:128. doi: 10.1038/s41420-019-0206-2 (PMC6689035; doi:10.1038/s41420-019-0206-2)
Supplement: Supplementary file 4 — Supp. Figure 4 [file 41420_2019_206_MOESM4_ESM.pdf]

Supplementary Figure 4

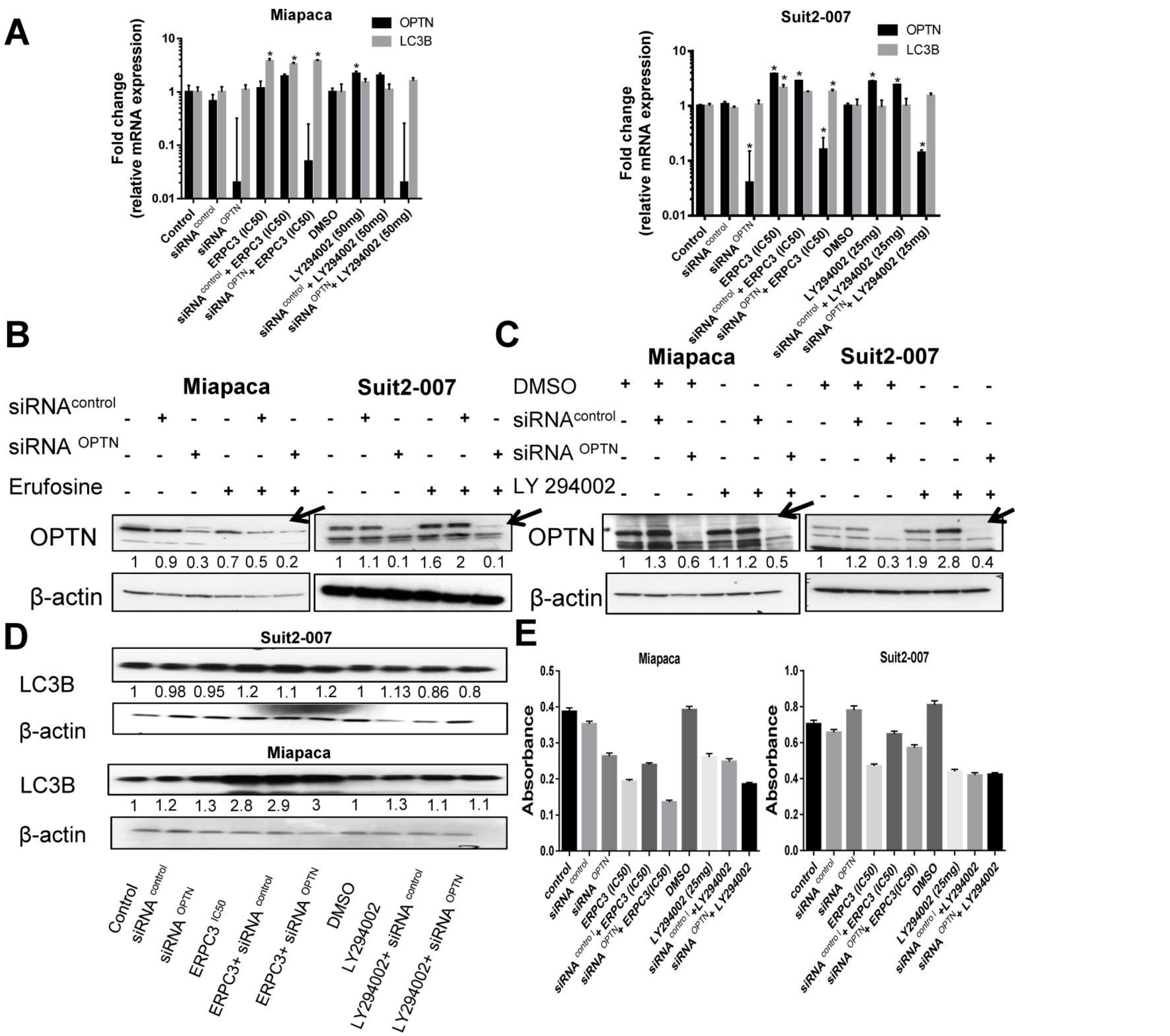

Supplementary Figure 4: Effects of combining OPTN knockdown with drugs modulating autophagy. **A:** mRNA expression of OPTN and LC3b in OPTN knockdown samples alone or combined with erufosine or LY294002. **B.:** WB of cells treated with the autophagy inducer erufosine (IC50), one day post- transfection with specific or non-specific siRNA to OPTN. 10-30µg protein was loaded per sample. Band densities were calculated via ImageJ software and adjusted to β-actin. **C:** WB of cells treated with the autophagy inhibitor LY294002 (25 µM for Suit2-007 and 50µM for Miapaca cells) one day post- transfection with specific or non-specific siRNA to OPTN. Band densities were calculated via ImageJ software and adjusted to β-actin. **D:** WB expression of LC3B in response to OPTN knockdown alone or in combination with erufosine and LY294002. Analysis of the band density was done by ImageJ software and corrected to β -actin as loading control. Further controls were the untreated control (erufosine) and the solvent DMSO (LY294002). **E:** MTT assay of the combination effect of OPTN KD with or without erufosine or LY 294002. The proliferation of Miapaca and Suit2-007 cells was measured at 540 nm (reference of 690 nm). Figures were generated by GraphPad Prism software.
