# Supplementary material for: Optineurin downregulation induces endoplasmic reticulum stress, chaperone-mediated autophagy, and apoptosis in pancreatic cancer cells
Source: Cell Death Discov. 2019 Aug 9;5:128. doi: 10.1038/s41420-019-0206-2 (PMC6689035; doi:10.1038/s41420-019-0206-2)
Supplement: Supplementary file 5 — Supp. Table 1 [file 41420_2019_206_MOESM5_ESM.pdf]

Supplementary Table 1: Modulated genes in Miapaca cells in response to OPTN knockdown

Gene no.	Gene symbol	Entrez Gene Name	Expression fold change	Cellular location of the respective protein	Function / nature of the protein
		**Down-regulated genes**			
1	HMMR	hyaluronan mediated motility receptor	-2,586	Plasma Membrane	transmembrane receptor
2	P4HA2	prolyl 4-hydroxylase subunit alpha 2	-2,251	Cytoplasm	transporter
3	FAR1	fatty acyl-CoA reductase 1	-2,137	Cytoplasm	enzyme
4	NCKAP1	NCK associated protein 1	-2,133	Plasma Membrane	other
5	VDAC3P1	voltage dependent anion channel 3 pseudogene 1	-2,103	Other	other
6	CCT6P1	chaperonin containing TCP1 subunit 6 pseudogene 1	-2,081	Other	other
7	PSMA5	proteasome subunit alpha 5	-2,019	Cytoplasm	peptidase
8	CENPQ	centromere protein Q	-2,012	Nucleus	other
9	CCDC25	coiled-coil domain containing 25	-2,004	Cytoplasm	other
10	AAGAB	alpha and gamma adaptin binding protein	-1,987	Cytoplasm	other
11	SLMAP	sarcolemma associated protein	-1,985	Plasma Membrane	other
12	USP38	ubiquitin specific peptidase 38	-1,956	Other	peptidase
13	CCT6P3	chaperonin containing TCP1 subunit 6 pseudogene 3	-1,942	Other	other
14	C17orf53	chromosome 17 open reading frame 53	-1,92	Other	other
15	NUS1P1	NUS1 dehydrodolichyl diphosphate synthase subunit pseudogene 1	-1,878	Other	other
16	FAM35A	family with sequence similarity 35 member A	-1,867	Extracellular Space	other
17	NSRP1	nuclear speckle splicing regulatory protein 1	-1,864	Nucleus	other
18	BRWD1	bromodomain and WD repeat domain containing 1	-1,857	Nucleus	transcription regulator
19	G3BP2	G3BP stress granule assembly factor 2	-1,853	Cytoplasm	enzyme
20	COPZ1	coatomer protein complex subunit zeta 1	-1,837	Cytoplasm	transporter
21	SLC39A10	solute carrier family 39 member 10	-1,825	Extracellular Space	transporter
22	MFAP1	microfibril associated protein 1	-1,796	Extracellular Space	other
23	POLQ	DNA polymerase theta	-1,785	Nucleus	enzyme
24	CAV2	caveolin 2	-1,782	Plasma Membrane	other

25	DEPDC1	DEP domain containing 1	-1,775	Nucleus	transcription regulator
26	CCT6A	chaperonin containing TCP1 subunit 6A	-1,767	Cytoplasm	other
27	VDAC3	voltage dependent anion channel 3	-1,76	Cytoplasm	ion channel
28	SLC25A44	solute carrier family 25 member 44	-1,752	Cytoplasm	transporter
29	ARMCX5-GPRASP2	G protein-coupled receptor associated sorting protein 2	-1,752	Plasma Membrane	other
30	PPP1R12B	protein phosphatase 1 regulatory subunit 12B	-1,747	Cytoplasm	phosphatase
31	MAPRE1	microtubule associated protein RP/EB family member 1	-1,728	Cytoplasm	other
32	YWHAH	tyrosine 3-monooxygenase/tryptophan 5-monooxygenase activation protein eta	-1,726	Cytoplasm	transcription regulator
33	LEMD3	LEM domain containing 3	-1,724	Nucleus	other
34	CSTF3	cleavage stimulation factor subunit 3	-1,724	Nucleus	other
35	SMYD2	SET and MYND domain containing 2	-1,723	Cytoplasm	enzyme
36	BNIP3L	BCL2 interacting protein 3 like	-1,72	Cytoplasm	other
37	STARD7	StAR related lipid transfer domain containing 7	-1,713	Cytoplasm	other
38	PHKB	phosphorylase kinase regulatory subunit beta	-1,709	Cytoplasm	kinase
39	CENPK	centromere protein K	-1,698	Nucleus	other
40	LBR	lamin B receptor	-1,697	Nucleus	enzyme
41	E2F2	E2F transcription factor 2	-1,689	Nucleus	transcription regulator
42	MGAT5	mannosyl (alpha-1,6-)-glycoprotein beta-1,6-N-acetyl-glucosaminyltransferase	-1,687	Cytoplasm	enzyme
43	PIGA	phosphatidylinositol glycan anchor biosynthesis class A	-1,686	Cytoplasm	enzyme
44	ATPAF1	ATP synthase mitochondrial F1 complex assembly factor 1	-1,684	Cytoplasm	other
45	ARMCX3	armadillo repeat containing, X-linked 3	-1,684	Cytoplasm	other
46	PM20D2	peptidase M20 domain containing 2	-1,669	Nucleus	peptidase
47	IGF2BP2	insulin like growth factor 2 mRNA binding protein 2	-1,668	Cytoplasm	translation regulator
48	CMAS	cytidine monophosphate N-acetylneuraminic acid synthetase	-1,663	Nucleus	enzyme
49	YWHAZ	tyrosine 3-monooxygenase/tryptophan 5-monooxygenase activation protein zeta	-1,635	Cytoplasm	enzyme
50	CENPI	centromere protein I	-1,63	Nucleus	other
51	FAM214B	family with sequence similarity 214 member B	-1,623	Nucleus	other
52	ZNF622	zinc finger protein 622	-1,622	Nucleus	other

53	MAPK6	mitogen-activated protein kinase 6	-1,621	Cytoplasm	kinase
54	AKAP11	A-kinase anchoring protein 11	-1,62	Cytoplasm	other
55	API5	apoptosis inhibitor 5	-1,62	Cytoplasm	other
56	EIF4G1	eukaryotic translation initiation factor 4 gamma 1	-1,618	Cytoplasm	translation regulator
57	CKAP2L	cytoskeleton associated protein 2 like	-1,61	Cytoplasm	other
58	KIAA0586	KIAA0586	-1,605	Cytoplasm	other
59	ACLY	ATP citrate lyase	-1,604	Cytoplasm	enzyme
60	CCDC68	coiled-coil domain containing 68	-1,603	Other	other
61	SERP1	stress associated endoplasmic reticulum protein 1	-1,6	Cytoplasm	other
62	MIPEP	mitochondrial intermediate peptidase	-1,599	Cytoplasm	peptidase
63	TAF2	TATA-box binding protein associated factor 2	-1,598	Nucleus	transcription regulator
64	ZCCHC3	zinc finger CCHC-type containing 3	-1,594	Other	other
65	BMPR2	bone morphogenetic protein receptor type 2	-1,593	Plasma Membrane	kinase
66	KIAA1671	KIAA1671	-1,584	Other	other
67	POLD1	DNA polymerase delta 1, catalytic subunit	-1,579	Nucleus	enzyme
68	RBL2	RB transcriptional corepressor like 2	-1,575	Nucleus	other
69	COPS7B	COP9 signalosome subunit 7B	-1,574	Cytoplasm	other
70	C1orf112	chromosome 1 open reading frame 112	-1,573	Other	other
71	CALM1 (includes others)	calmodulin 1	-1,572	Cytoplasm	other
72	OSBPL8	oxysterol binding protein like 8	-1,57	Plasma Membrane	transporter
73	LASP1	LIM and SH3 protein 1	-1,568	Cytoplasm	transporter
74	C1GALT1C1	C1GALT1 specific chaperone 1	-1,565	Cytoplasm	other
75	BZW2	basic leucine zipper and W2 domains 2	-1,565	Cytoplasm	translation regulator
76	ACYP1	acylphosphatase 1	-1,56	Cytoplasm	enzyme
77	RIF1	replication timing regulatory factor 1	-1,553	Nucleus	other
78	TTYH3	tweety family member 3	-1,553	Plasma Membrane	ion channel
79	CDC45	cell division cycle 45	-1,549	Nucleus	other
80	MCM2	minichromosome maintenance complex component 2	-1,548	Nucleus	enzyme
81	SAE1	SUMO1 activating enzyme subunit 1	-1,546	Cytoplasm	enzyme
82	C9orf40	chromosome 9 open reading frame 40	-1,54	Other	other
83	LRRC20	leucine rich repeat containing 20	-1,538	Other	other
84	C5orf51	chromosome 5 open reading frame 51	-1,537	Other	other

85	AMFR	autocrine motility factor receptor	-1,535	Plasma Membrane	transmembrane receptor
86	RPN1	ribophorin I	-1,535	Cytoplasm	enzyme
87	PCGF6	polycomb group ring finger 6	-1,534	Nucleus	transcription regulator
88	MYO10	myosin X	-1,534	Cytoplasm	enzyme
89	DHX29	DExH-box helicase 29	-1,533	Cytoplasm	enzyme
90	DCBLD2	discoidin, CUB and LCCL domain containing 2	-1,531	Plasma Membrane	other
91	IDI1	isopentenyl-diphosphate delta isomerase 1	-1,531	Cytoplasm	enzyme
92	CIRBP	cold inducible RNA binding protein	-1,528	Nucleus	translation regulator
93	KRR1	KRR1, small subunit processome component homolog	-1,527	Nucleus	other
94	MAPK1	mitogen-activated protein kinase 1	-1,525	Cytoplasm	kinase
95	PARG	poly(ADP-ribose) glycohydrolase	-1,524	Cytoplasm	enzyme
96	TBL1XR1	transducin beta like 1 X-linked receptor 1	-1,519	Nucleus	transcription regulator
97	STK38	serine/threonine kinase 38	-1,519	Nucleus	kinase
98	TIGAR	TP53 induced glycolysis regulatory phosphatase	-1,519	Cytoplasm	enzyme
99	TTC33	tetratricopeptide repeat domain 33	-1,519	Other	other
100	ELP5	elongator acetyltransferase complex subunit 5	-1,518	Cytoplasm	other
101	VKORC1L1	vitamin K epoxide reductase complex subunit 1 like 1	-1,511	Cytoplasm	enzyme
102	PKP4	plakophilin 4	-1,509	Plasma Membrane	other
103	DLAT	dihydrolipoamide S-acetyltransferase	-1,508	Cytoplasm	enzyme
104	BARX2	BARX homeobox 2	-1,506	Nucleus	transcription regulator
105	KIF2C	kinesin family member 2C	-1,506	Nucleus	other
106	NUP88	nucleoporin 88	-1,506	Nucleus	transporter
107	MASTL	microtubule associated serine/threonine kinase like	-1,506	Cytoplasm	kinase
108	NEDD1	neural precursor cell expressed, developmentally down-regulated 1	-1,505	Cytoplasm	other
109	CENPE	centromere protein E	-1,504	Nucleus	other
110	TACC1	transforming acidic coiled-coil containing protein 1	-1,503	Nucleus	other
111	MAD2L1	mitotic arrest deficient 2 like 1	-1,503	Nucleus	other
112	PGAM1P8	phosphoglycerate mutase 1 pseudogene 8	-1,502	Other	other

113	HMGB1P10	high mobility group box 1 pseudogene 10	-1,501	Nucleus	other
		**Up-regulated genes**			
114	RPL14	ribosomal protein L14	1,502	Cytoplasm	other
115	PMAIP1	phorbol-12-myristate-13-acetate-induced protein 1	1,502	Cytoplasm	other
116	AES	amino-terminal enhancer of split	1,503	Nucleus	transcription regulator
117	HAX1	HCLS1 associated protein X-1	1,504	Cytoplasm	other
118	OCRL	OCRL, inositol polyphosphate-5-phosphatase	1,504	Cytoplasm	phosphatase
119	PIP4K2B	phosphatidylinositol-5-phosphate 4-kinase type 2 beta	1,504	Cytoplasm	kinase
120	RNF146	ring finger protein 146	1,505	Cytoplasm	enzyme
121	BTRC	beta-transducin repeat containing E3 ubiquitin protein ligase	1,506	Cytoplasm	enzyme
122	DNAJB6	DnaJ heat shock protein family (Hsp40) member B6	1,506	Nucleus	transcription regulator
123	APOLD1	apolipoprotein L domain containing 1	1,509	Other	other
124	ULK4P1	ULK4 pseudogene 1	1,51	Other	other
125	SARS2	seryl-tRNA synthetase 2, mitochondrial	1,512	Cytoplasm	enzyme
126	NQO1	NAD(P)H quinone dehydrogenase 1	1,513	Cytoplasm	enzyme
127	CFD	complement factor D	1,514	Extracellular Space	peptidase
128	IFI27L2	interferon alpha inducible protein 27 like 2	1,514	Other	other
129	ATP1B1	ATPase Na+/K+ transporting subunit beta 1	1,515	Plasma Membrane	transporter
130	PABPC1P4	poly(A) binding protein cytoplasmic 1 pseudogene 4	1,52	Other	other
131	SPNS1	sphingolipid transporter 1 (putative)	1,521	Cytoplasm	transporter
132	RPL17P36	ribosomal protein L17 pseudogene 36	1,522	Other	other
133	RPS4XP16	ribosomal protein S4X pseudogene 16	1,522	Other	other
134	TFPI	tissue factor pathway inhibitor	1,522	Extracellular Space	other
135	SLC35E1	solute carrier family 35 member E1	1,523	Cytoplasm	other
136	STX4	syntaxin 4	1,525	Plasma Membrane	transporter
137	SLC44A1	solute carrier family 44 member 1	1,525	Plasma Membrane	transporter
138	FSD1	fibronectin type III and SPRY domain containing 1	1,525	Cytoplasm	other
139	CREBRF	CREB3 regulatory factor	1,529	Nucleus	transcription regulator
140	SELENOM	selenoprotein M	1,532	Cytoplasm	other

141	HTATIP2	HIV-1 Tat interactive protein 2	1,536	Nucleus	transcription regulator
142	C6orf48	chromosome 6 open reading frame 48	1,537	Other	other
143	WARS	tryptophanyl-tRNA synthetase	1,538	Cytoplasm	enzyme
144	WWP2	WW domain containing E3 ubiquitin protein ligase 2	1,539	Cytoplasm	enzyme
145	CAMK1	calcium/calmodulin dependent protein kinase I	1,539	Cytoplasm	kinase
146	EEF1B2	eukaryotic translation elongation factor 1 beta 2	1,542	Cytoplasm	translation regulator
147	NOL3	nucleolar protein 3	1,544	Nucleus	other
148	HTRA1	HtrA serine peptidase 1	1,546	Extracellular Space	peptidase
149	C15orf40	chromosome 15 open reading frame 40	1,546	Other	other
150	KRT40	keratin 40	1,548	Cytoplasm	other
151	NUB1	negative regulator of ubiquitin like proteins 1	1,549	Nucleus	other
152	ERC1	ELKS/RAB6-interacting/CAST family member 1	1,553	Cytoplasm	other
153	HIST2H2AA3 / HIST2H2AA4	histone cluster 2 H2A family member a3	1,554	Nucleus	other
154	CTH	cystathionine gamma-lyase	1,554	Cytoplasm	enzyme
155	UPF3B	UPF3B, regulator of nonsense mediated mRNA decay	1,555	Nucleus	transporter
156	RPLP1	ribosomal protein lateral stalk subunit P1	1,555	Cytoplasm	other
157	HMGA2	high mobility group AT-hook 2	1,558	Nucleus	enzyme
158	GRN	granulin precursor	1,559	Extracellular Space	growth factor
159	CCNB1IP1	cyclin B1 interacting protein 1	1,564	Nucleus	enzyme
160	DCAF13	DDB1 and CUL4 associated factor 13	1,564	Nucleus	other
161	RAB1C	RAB1C, member RAS oncogene family pseudogene	1,565	Other	other
162	COLGALT2	collagen beta(1-O)galactosyltransferase 2	1,565	Cytoplasm	enzyme
163	RPL12P14	ribosomal protein L12 pseudogene 14	1,565	Other	other
164	HTR1D	5-hydroxytryptamine receptor 1D	1,566	Plasma Membrane	G-protein coupled receptor
165	UNC119	unc-119 lipid binding chaperone	1,566	Cytoplasm	other
166	C12orf75	chromosome 12 open reading frame 75	1,571	Other	other
167	TSTA3	tissue specific transplantation antigen P35B	1,572	Plasma Membrane	enzyme
168	PTMA	prothymosin, alpha	1,573	Nucleus	other
169	GNG5	G protein subunit gamma 5	1,574	Plasma Membrane	other
170	CALB2	calbindin 2	1,579	Cytoplasm	other

171	LOC441087	uncharacterized LOC441087	1,581	Other	other
172	TRIB3	tribbles pseudokinase 3	1,583	Nucleus	kinase
173	HIST2H2BE	histone cluster 2 H2B family member e	1,585	Nucleus	other
174	N4BP2L2	NEDD4 binding protein 2 like 2	1,586	Nucleus	transcription regulator
175	MYO5A	myosin VA	1,587	Cytoplasm	enzyme
176	ZFP36	ZFP36 ring finger protein	1,587	Nucleus	transcription regulator
177	RNA28SN5	RNA, 28S ribosomal N5	1,59	Other	other
178	MCL1	MCL1, BCL2 family apoptosis regulator	1,59	Cytoplasm	transporter
179	CEBPB	CCAAT/enhancer binding protein beta	1,591	Nucleus	transcription regulator
180	TMEM238	transmembrane protein 238	1,593	Other	other
181	MED1	mediator complex subunit 1	1,595	Nucleus	transcription regulator
182	RPL26P6	ribosomal protein L26 pseudogene 6	1,597	Other	other
183	WDR41	WD repeat domain 41	1,598	Cytoplasm	other
184	ADARB1	adenosine deaminase, RNA specific B1	1,598	Nucleus	enzyme
185	SMARCD3	SWI/SNF related, matrix associated, actin dependent regulator of chromatin, subfamily d, member 3	1,599	Nucleus	transcription regulator
186	RNF149	ring finger protein 149	1,6	Cytoplasm	enzyme
187	CARS	cysteinyl-tRNA synthetase	1,603	Cytoplasm	enzyme
188	HMGN1	high mobility group nucleosome binding domain 1	1,603	Nucleus	transcription regulator
189	TNFRSF25	TNF receptor superfamily member 25	1,611	Plasma Membrane	transmembrane receptor
190	RPL14P1	ribosomal protein L14 pseudogene 1	1,618	Other	other
191	AMMECR1	Alport syndrome, mental retardation, midface hypoplasia and elliptocytosis chromosomal region gene 1	1,621	Nucleus	other
192	PPP2CB	protein phosphatase 2 catalytic subunit beta	1,622	Cytoplasm	phosphatase
193	HIST3H2A	histone cluster 3 H2A	1,627	Nucleus	other
194	UBE2G2	ubiquitin conjugating enzyme E2 G2	1,631	Cytoplasm	enzyme
195	NBDY	negative regulator of P-body association	1,635	Other	other

196	HOXC6	homeobox C6	1,639	Nucleus	transcription regulator
197	C12orf57	chromosome 12 open reading frame 57	1,639	Cytoplasm	other
198	SNRPN	small nuclear ribonucleoprotein polypeptide N	1,64	Nucleus	other
199	PCBD1	pterin-4 alpha-carbinolamine dehydratase 1	1,642	Nucleus	transcription regulator
200	CXCL5	C-X-C motif chemokine ligand 5	1,654	Extracellular Space	cytokine
201	EPB41L4A-AS1	EPB41L4A antisense RNA 1	1,656	Other	other
202	CXCL16	C-X-C motif chemokine ligand 16	1,661	Extracellular Space	cytokine
203	RIOX2	ribosomal oxygenase 2	1,664	Nucleus	enzyme
204	CYBRD1	cytochrome b reductase 1	1,665	Cytoplasm	enzyme
205	NUPR1	nuclear protein 1, transcriptional regulator	1,667	Nucleus	transcription regulator
206	ATF4	activating transcription factor 4	1,679	Nucleus	transcription regulator
207	FTH1P3	ferritin heavy chain 1 pseudogene 3	1,681	Other	other
208	SLC6A9	solute carrier family 6 member 9	1,681	Plasma Membrane	transporter
209	FTH1P20	ferritin heavy chain 1 pseudogene 20	1,682	Other	other
210	MSRA	methionine sulfoxide reductase A	1,693	Cytoplasm	enzyme
211	CNTNAP1	contactin associated protein 1	1,699	Plasma Membrane	other
212	EIF4EBP1	eukaryotic translation initiation factor 4E binding protein 1	1,701	Cytoplasm	translation regulator
213	RPS26P31	ribosomal protein S26 pseudogene 31	1,705	Other	other
214	BCHE	butyrylcholinesterase	1,706	Plasma Membrane	enzyme
215	LGALS3	galectin 3	1,71	Extracellular Space	other
216	HIST1H2BK	histone cluster 1 H2B family member k	1,723	Nucleus	other
217	CAT	catalase	1,727	Cytoplasm	enzyme
218	ABCA9	ATP binding cassette subfamily A member 9	1,729	Cytoplasm	transporter
219	DGCR6/LOC102724770	DiGeorge syndrome critical region gene 6	1,743	Nucleus	other
220	CLIC4	chloride intracellular channel 4	1,743	Plasma Membrane	ion channel
221	FAM213A	family with sequence similarity 213 member A	1,747	Extracellular Space	other
222	FERMT2	fermitin family member 2	1,754	Cytoplasm	other
223	ALG13	ALG13, UDP-N-acetylglucosaminyltransferase subunit	1,771	Cytoplasm	enzyme
224	GADD45A	growth arrest and DNA damage inducible alpha	1,777	Nucleus	other

225	GPT2	glutamic--pyruvic transaminase 2	1,778	Cytoplasm	enzyme
226	RHOQ	ras homolog family member Q	1,789	Plasma Membrane	enzyme
227	ABCA10	ATP binding cassette subfamily A member 10	1,79	Other	transporter
228	DDIT4	DNA damage inducible transcript 4	1,806	Cytoplasm	other
229	DNASE2	deoxyribonuclease 2, lysosomal	1,823	Cytoplasm	enzyme
230	ASS1	argininosuccinate synthase 1	1,837	Cytoplasm	enzyme
231	RHOQP3	ras homolog family member Q pseudogene 3	1,844	Other	other
232	SLC3A2	solute carrier family 3 member 2	1,848	Plasma Membrane	transporter
233	SLC7A5	solute carrier family 7 member 5	1,877	Plasma Membrane	transporter
234	PSPH	phosphoserine phosphatase	1,878	Cytoplasm	phosphatase
235	PSAT1P3	phosphoserine aminotransferase 1 pseudogene 3	1,893	Other	other
236	KIF1A	kinesin family member 1A	2,001	Cytoplasm	other
237	HIST1H2BD	histone cluster 1 H2B family member d	2,135	Nucleus	other
238	PSAT1	phosphoserine aminotransferase 1	2,279	Cytoplasm	enzyme
239	PCK2	phosphoenolpyruvate carboxykinase 2, mitochondrial	2,291	Cytoplasm	kinase
240	PHGDH	phosphoglycerate dehydrogenase	2,307	Cytoplasm	enzyme
241	INHBE	inhibin beta E subunit	2,34	Extracellular Space	growth factor
