# Supplementary material for: Optineurin downregulation induces endoplasmic reticulum stress, chaperone-mediated autophagy, and apoptosis in pancreatic cancer cells
Source: Cell Death Discov. 2019 Aug 9;5:128. doi: 10.1038/s41420-019-0206-2 (PMC6689035; doi:10.1038/s41420-019-0206-2)
Supplement: Supplementary file 6 — Supp. Table 2 [file 41420_2019_206_MOESM6_ESM.pdf]

Supplementary Table 2: 52 genes modulated in Miapaca and Suit2-007 PDAC cells

Gene no.	Gene symbol	Entrez Gene Name	Expression fold change Miapaca	Expression fold change Suit2-007	Cellular location of the respective protein	Function / nature of the protein
		**Down-regulated genes**				
1	HSPE1	heat shock protein family E (Hsp10) member 1	-3,096	-2,377	Cytoplasm	enzyme
2	OPTN	optineurin	-3,022	-2,627	Cytoplasm	other
3	PRMT6	protein arginine methyltransferase 6	-2,467	-1,763	Nucleus	enzyme
4	CNN2	calponin 2	-2,408	-1,79	Cytoplasm	other
5	CDK6	cyclin dependent kinase 6	-2,38	-1,756	Nucleus	kinase
6	F2R	coagulation factor II thrombin receptor	-2,312	-1,575	Plasma Membrane	G-protein coupled receptor
7	RMI2	RecQ mediated genome instability 2	-2,304	-1,533	Nucleus	other
8	SKP1	S-phase kinase associated protein 1	-2,253	-1,743	Nucleus	transcription regulator
9	TMEM109	transmembrane protein 109	-2,236	-1,965	Cytoplasm	other
10	BORA	bora, aurora kinase A activator	-2,199	-1,572	Cytoplasm	other
11	PDIA5	protein disulfide isomerase family A member 5	-2,155	-1,978	Cytoplasm	enzyme
12	FKBP3	FK506 binding protein 3	-2,112	-1,507	Nucleus	enzyme
13	EMG1	EMG1, N1-specific pseudouridine methyltransferase	-2,093	-1,563	Nucleus	enzyme
14	FRMD8	FERM domain containing 8	-2,02	-1,683	Other	other
15	CD81	CD81 molecule	-2,017	-1,653	Plasma Membrane	other
16	CDCA8	cell division cycle associated 8	-2,015	-1,835	Nucleus	other
17	CNIH1	cornichon family AMPA receptor auxiliary protein 1	-1,993	-1,865	Plasma Membrane	other
18	LRRC47	leucine rich repeat containing 47	-1,979	-1,632	Other	other
19	STMN1	stathmin 1	-1,952	-1,536	Cytoplasm	other
20	COMMD7	COMM domain containing 7	-1,934	-1,871	Cytoplasm	other

21	DCAF6	DDB1 and CUL4 associated factor 6	-1,93	-1,83	Nucleus	transcription regulator
22	TMEM199	transmembrane protein 199	-1,916	-1,531	Cytoplasm	other
23	HSP90AB1	heat shock protein 90 alpha family class B member 1	-1,858	-1,583	Cytoplasm	enzyme
24	PRTFDC1	phosphoribosyl transferase domain containing 1	-1,851	-1,57	Cytoplasm	enzyme
25	HMBS	hydroxymethylbilane synthase	-1,85	-1,703	Cytoplasm	enzyme
26	H3F3A/H3F3B	H3 histone family member 3A	-1,821	-1,588	Nucleus	other
27	CCNE1	cyclin E1	-1,788	-1,595	Nucleus	transcription regulator
28	TEAD2	TEA domain transcription factor 2	-1,745	-1,578	Nucleus	transcription regulator
29	HMCES	5-hydroxymethylcytosine (hmC) binding, ES cell-specific	-1,717	-1,505	Other	other
30	H3F3AP6	H3 histone, family 3A, pseudogene 6	-1,715	-1,538	Other	other
31	POFUT1	protein O-fucosyltransferase 1	-1,675	-1,72	Cytoplasm	enzyme
32	AZIN1	antizyme inhibitor 1	-1,657	-1,547	Cytoplasm	enzyme
33	TOMM20	translocase of outer mitochondrial membrane 20	-1,656	-1,51	Cytoplasm	transporter
34	COASY	Coenzyme A synthase	-1,647	-1,751	Cytoplasm	kinase
35	TMEM50B	transmembrane protein 50B	-1,607	-1,641	Plasma Membrane	other
36	ISCU	iron-sulfur cluster assembly enzyme	-1,548	-1,829	Cytoplasm	other
37	PPM1F	protein phosphatase, Mg2+/Mn2+ dependent 1F	-1,533	-1,728	Cytoplasm	phosphatase
38	HSPA1A/HSPA1B	heat shock protein family A (Hsp70) member 1A	-1,502	-1,55	Cytoplasm	enzyme
		**Up-regulated genes**				
39	ANO1	anoctamin 1	1,573	1,887	Plasma Membrane	ion channel
40	LAMP2	lysosomal associated membrane protein 2	1,598	1,612	Plasma Membrane	enzyme

41	AASDHPPT	aminoadipate-semialdehyde dehydrogenase-phosphopantetheinyl transferase	1,605	1,505	Cytoplasm	enzyme
42	HEATR1	HEAT repeat containing 1	1,635	1,553	Nucleus	other
43	ARHGEF2	Rho/Rac guanine nucleotide exchange factor 2	1,664	1,856	Cytoplasm	other
44	BCAT1	branched chain amino acid transaminase 1	1,689	1,534	Cytoplasm	enzyme
45	CAVIN2	caveolae associated protein 2	1,798	1,533	Plasma Membrane	other
46	FAM129A	family with sequence similarity 129 member A	1,81	1,892	Cytoplasm	other
47	CLDN1	claudin 1	1,948	1,853	Plasma Membrane	other
48	DESI2	desumoylating isopeptidase 2	2,063	2,519	Cytoplasm	other
49	ASNS	asparagine synthetase (glutamine-hydrolyzing)	2,082	1,785	Cytoplasm	enzyme
		**Differently modulated genes**				
50	IFITM3	interferon induced transmembrane protein 3	1,524	-1,713	Plasma Membrane	other
51	SLC15A3	solute carrier family 15 member 3	1,53	-1,913	Cytoplasm	transporter
52	TAF5	TATA-box binding protein associated factor 5	-1,606	1,52	Nucleus	transcription regulator
