# Supplementary material for: Optineurin downregulation induces endoplasmic reticulum stress, chaperone-mediated autophagy, and apoptosis in pancreatic cancer cells
Source: Cell Death Discov. 2019 Aug 9;5:128. doi: 10.1038/s41420-019-0206-2 (PMC6689035; doi:10.1038/s41420-019-0206-2)
Supplement: Supplementary file 7 — Supp. Table 3 [file 41420_2019_206_MOESM7_ESM.pdf]

Supplementary Table 3: Modulated genes in Suit2-007 cells in response to OPTN knockdown

Gene no.	Gene symbol	Entrez Gene Name	Expression fold change	Cellular location of the respective protein	Function / nature of the protein
		**Down-regulated genes**			
1	IFI27	interferon alpha inducible protein 27	-5,129	Cytoplasm	other
2	IFI6	interferon alpha inducible protein 6	-3,763	Cytoplasm	other
3	MX1	MX dynamin like GTPase 1	-2,588	Cytoplasm	enzyme
4	OAS2	2'-5'-oligoadenylate synthetase 2	-2,48	Cytoplasm	enzyme
5	IFITM1	interferon induced transmembrane protein 1	-2,318	Plasma Membrane	transmembrane receptor
6	SMIM32	small integral membrane protein 32	-2,302	Other	other
7	IRF9	interferon regulatory factor 9	-2,147	Nucleus	transcription regulator
8	BST2	bone marrow stromal cell antigen 2	-2,107	Plasma Membrane	other
9	H3F3AP4	H3 histone, family 3A, pseudogene 4	-1,965	Other	other
10	MSRB2	methionine sulfoxide reductase B2	-1,943	Nucleus	transcription regulator
11	ISG15	ISG15 ubiquitin-like modifier	-1,935	Extracellular Space	other
12	UBE2L6	ubiquitin conjugating enzyme E2 L6	-1,911	Cytoplasm	enzyme
13	VGLL4	vestigial like family member 4	-1,897	Nucleus	other
14	SLC2A2	solute carrier family 2 member 2	-1,894	Plasma Membrane	transporter
15	SLC22A3	solute carrier family 22 member 3	-1,843	Plasma Membrane	transporter
16	FCGBP	Fc fragment of IgG binding protein	-1,835	Extracellular Space	other
17	FXYD2	FXYD domain containing ion transport regulator 2	-1,827	Plasma Membrane	ion channel
18	RAB40B	RAB40B, member RAS oncogene family	-1,807	Plasma Membrane	enzyme
19	ARRB1	arrestin beta 1	-1,8	Cytoplasm	other
20	POLR3A	RNA polymerase III subunit A	-1,786	Nucleus	enzyme
21	VAV3	vav guanine nucleotide exchange factor 3	-1,783	Extracellular Space	cytokine
22	HLA-DMB	major histocompatibility complex, class II, DM beta	-1,77	Plasma Membrane	transmembrane receptor

23	IFIT1	interferon induced protein with tetratricopeptide repeats 1	-1,759	Cytoplasm	other
24	RGPD4 (includes others)	RANBP2-like and GRIP domain containing 5	-1,754	Nucleus	enzyme
25	ANKRD33	ankyrin repeat domain 33	-1,751	Nucleus	transcription regulator
26	OAS1	2'-5'-oligoadenylate synthetase 1	-1,74	Cytoplasm	enzyme
27	SLC16A12	solute carrier family 16 member 12	-1,721	Plasma Membrane	transporter
28	OCIAD1	OCIA domain containing 1	-1,719	Cytoplasm	other
29	S100A4	S100 calcium binding protein A4	-1,718	Cytoplasm	other
30	SPACA6	sperm acrosome associated 6	-1,712	Other	other
31	ZNF738	zinc finger protein 738	-1,712	Other	other
32	VSIR	V-set immunoregulatory receptor	-1,706	Plasma Membrane	other
33	TOM1	target of myb1 membrane trafficking protein	-1,696	Cytoplasm	transporter
34	N4BP2	NEDD4 binding protein 2	-1,691	Cytoplasm	enzyme
35	IP6K1	inositol hexakisphosphate kinase 1	-1,688	Cytoplasm	kinase
36	PNPT1	polyribonucleotide nucleotidyltransferase 1	-1,675	Cytoplasm	enzyme
37	DNAJC15	DnaJ heat shock protein family (Hsp40) member C15	-1,661	Cytoplasm	other
38	LOC729603	calcineurin like EF-hand protein 1 pseudogene	-1,657	Other	other
39	PGAM1	phosphoglycerate mutase 1	-1,639	Cytoplasm	phosphatase
40	ZNF486	zinc finger protein 486	-1,632	Nucleus	other
41	BLZF1	basic leucine zipper nuclear factor 1	-1,629	Cytoplasm	transcription regulator
42	DUXAP10	double homeobox A pseudogene 10	-1,626	Other	other
43	ZNF682	zinc finger protein 682	-1,621	Nucleus	other
44	MIR22HG	MIR22 host gene	-1,616	Other	other
45	SULT1A1	sulfotransferase family 1A member 1	-1,614	Cytoplasm	enzyme
46	ZBED3	zinc finger BED-type containing 3	-1,607	Cytoplasm	other
47	GAS5	growth arrest specific 5 (non-protein coding)	-1,602	Other	other
48	NFKBIZ	NFKB inhibitor zeta	-1,601	Nucleus	transcription regulator
49	PDXK	pyridoxal kinase	-1,599	Cytoplasm	kinase

50	DMC1	DNA meiotic recombinase 1	-1,598	Nucleus	enzyme
51	CSF2RA	colony stimulating factor 2 receptor alpha subunit	-1,596	Plasma Membrane	transmembrane receptor
52	LCOR	ligand dependent nuclear receptor corepressor	-1,595	Nucleus	transcription regulator
53	AKR1D1	aldo-keto reductase family 1 member D1	-1,592	Cytoplasm	enzyme
54	IRF7	interferon regulatory factor 7	-1,584	Nucleus	transcription regulator
55	ABRAXAS1	abraxas 1, BRCA1 A complex subunit	-1,582	Nucleus	other
56	HOXA10	homeobox A10	-1,578	Nucleus	transcription regulator
57	LACTB	lactamase beta	-1,575	Cytoplasm	peptidase
58	LARGE1	LARGE xylosyl- and glucuronyltransferase 1	-1,571	Cytoplasm	enzyme
59	SLC10A3	solute carrier family 10 member 3	-1,567	Plasma Membrane	transporter
60	RXRB	retinoid X receptor beta	-1,566	Nucleus	ligand-dependent nuclear receptor
61	OPA3	OPA3, outer mitochondrial membrane lipid metabolism regulator	-1,559	Cytoplasm	other
62	C21orf58	chromosome 21 open reading frame 58	-1,558	Other	other
63	RHBDF1	rhomboid 5 homolog 1	-1,555	Cytoplasm	other
64	HLA-DMA	major histocompatibility complex, class II, DM alpha	-1,555	Plasma Membrane	transmembrane receptor
65	TMEM150A	transmembrane protein 150A	-1,547	Plasma Membrane	other
66	LOC100134868	uncharacterized LOC100134868	-1,546	Other	other
67	TCEA2	transcription elongation factor A2	-1,544	Nucleus	transcription regulator
68	LGALS7/LGALS7B	galectin 7	-1,539	Extracellular Space	other
69	MCM8	minichromosome maintenance 8 homologous recombination repair factor	-1,536	Nucleus	enzyme
70	LRCH4	leucine rich repeats and calponin homology domain containing 4	-1,535	Cytoplasm	transcription regulator
71	NARF	nuclear prelamin A recognition factor	-1,531	Nucleus	enzyme

72	LINC01018	long intergenic non-protein coding RNA 1018	-1,53	Other	other
73	TNFSF10	TNF superfamily member 10	-1,528	Extracellular Space	cytokine
74	RARRES3	retinoic acid receptor responder 3	-1,527	Cytoplasm	enzyme
75	SETD1A	SET domain containing 1A	-1,526	Nucleus	ion channel
76	SMIM1	small integral membrane protein 1 (Vel blood group)	-1,525	Cytoplasm	other
77	RAB42P1	RAB42, member RAS oncogene family, pseudogene 1	-1,522	Other	other
78	ENKD1	enkurin domain containing 1	-1,522	Cytoplasm	other
79	SLC48A1	solute carrier family 48 member 1	-1,521	Cytoplasm	transporter
80	CCDC24	coiled-coil domain containing 24	-1,521	Other	other
81	REXO5	RNA exonuclease 5	-1,518	Nucleus	enzyme
82	NENF	neudesin neurotrophic factor	-1,517	Extracellular Space	growth factor
83	PSG4	pregnancy specific beta-1-glycoprotein 4	-1,516	Extracellular Space	other
84	HAUS2	HAUS augmin like complex subunit 2	-1,512	Cytoplasm	other
85	CREB1	cAMP responsive element binding protein 1	-1,51	Nucleus	transcription regulator
86	DEXI	Dexi homolog	-1,509	Other	other
87	DHRS11	dehydrogenase/reductase 11	-1,508	Other	other
88	DNAJC28	DnaJ heat shock protein family (Hsp40) member C28	-1,507	Other	other
89	SLC2A3P1	solute carrier family 2 member 3 pseudogene 1	-1,501	Other	other
90	IFITM2	interferon induced transmembrane protein 2	-1,501	Cytoplasm	other
		**Up-regulated genes**			
91	ANKRD10	ankyrin repeat domain 10	1,501	Nucleus	transcription regulator
92	SH3KBP1	SH3 domain containing kinase binding protein 1	1,501	Cytoplasm	other
93	GALNT1	polypeptide N-acetylgalactosaminyltransferase 1	1,504	Cytoplasm	enzyme
94	DLGAP1-AS5	DLGAP1 antisense RNA 5	1,504	Other	other
95	NRP1	neuropilin 1	1,505	Plasma Membrane	transmembrane receptor
96	TNFRSF21	TNF receptor superfamily member 21	1,505	Plasma Membrane	transmembrane receptor

97	FLI1	Fli-1 proto-oncogene, ETS transcription factor	1,507	Nucleus	transcription regulator
98	KLHL7	kelch like family member 7	1,508	Nucleus	other
99	FOXA2	forkhead box A2	1,508	Nucleus	transcription regulator
100	DDX21	DExD-box helicase 21	1,508	Nucleus	enzyme
101	LRRC58	leucine rich repeat containing 58	1,509	Other	other
102	ARHGAP22	Rho GTPase activating protein 22	1,51	Cytoplasm	other
103	CPSF2	cleavage and polyadenylation specific factor 2	1,51	Nucleus	other
104	MATR3	matrin 3	1,511	Nucleus	other
105	LINC00239	long intergenic non-protein coding RNA 239	1,511	Other	other
106	CREB5	cAMP responsive element binding protein 5	1,512	Nucleus	transcription regulator
107	SPP1	secreted phosphoprotein 1	1,512	Extracellular Space	cytokine
108	NUDT11	nudix hydrolase 11	1,513	Cytoplasm	phosphatase
109	KIF2A	kinesin family member 2A	1,514	Cytoplasm	other
110	RBM24	RNA binding motif protein 24	1,514	Other	other
111	NDUFAF5	NADH:ubiquinone oxidoreductase complex assembly factor 5	1,514	Cytoplasm	other
112	SUPT3H	SPT3 homolog, SAGA and STAGA complex component	1,515	Nucleus	transcription regulator
113	HMGN1P30	high mobility group nucleosome binding domain 1 pseudogene 30	1,517	Other	other
114	SARS	seryl-tRNA synthetase	1,517	Cytoplasm	enzyme
115	BRI3BP	BRI3 binding protein	1,519	Extracellular Space	other
116	ALDH1L2	aldehyde dehydrogenase 1 family member L2	1,523	Cytoplasm	enzyme
117	EPCAM	epithelial cell adhesion molecule	1,523	Plasma Membrane	other
118	TCEAL1	transcription elongation factor A like 1	1,524	Nucleus	transcription regulator
119	PPIAP80	peptidylprolyl isomerase A pseudogene 80	1,524	Other	other
120	FAM135A	family with sequence similarity 135 member A	1,524	Other	enzyme
121	PIM1	Pim-1 proto-oncogene, serine/threonine kinase	1,524	Cytoplasm	kinase
122	BUB3	BUB3, mitotic checkpoint protein	1,525	Nucleus	other

123	RAPH1	Ras association (RalGDS/AF-6) and pleckstrin homology domains 1	1,525	Plasma Membrane	other
124	WDR36	WD repeat domain 36	1,525	Extracellular Space	other
125	TARS	threonyl-tRNA synthetase	1,526	Nucleus	enzyme
126	CCNC	cyclin C	1,526	Nucleus	kinase
127	PPIAP35	peptidylprolyl isomerase A pseudogene 35	1,526	Other	other
128	NFXL1	nuclear transcription factor, X-box binding like 1	1,527	Nucleus	transcription regulator
129	ANLN	anillin actin binding protein	1,527	Cytoplasm	other
130	PDHX	pyruvate dehydrogenase complex component X	1,528	Cytoplasm	enzyme
131	ANXA2P1	annexin A2 pseudogene 1	1,531	Other	other
132	TMEM33	transmembrane protein 33	1,531	Cytoplasm	other
133	HNRNPUL1	heterogeneous nuclear ribonucleoprotein U like 1	1,532	Nucleus	other
134	PPT1	palmitoyl-protein thioesterase 1	1,533	Cytoplasm	enzyme
135	SQOR	sulfide quinone oxidoreductase	1,534	Cytoplasm	enzyme
136	KIAA2012	KIAA2012	1,537	Other	other
137	EGFR	epidermal growth factor receptor	1,539	Plasma Membrane	kinase
138	SEMA3C	semaphorin 3C	1,54	Extracellular Space	other
139	KLHL18	kelch like family member 18	1,54	Other	other
140	RIN2	Ras and Rab interactor 2	1,54	Cytoplasm	other
141	HDAC2	histone deacetylase 2	1,541	Nucleus	transcription regulator
142	ZNF680	zinc finger protein 680	1,541	Nucleus	other
143	RAB11FIP2	RAB11 family interacting protein 2	1,543	Cytoplasm	other
144	PYGL	glycogen phosphorylase L	1,544	Cytoplasm	enzyme
145	IGFBP4	insulin like growth factor binding protein 4	1,545	Extracellular Space	other
146	MIR100HG	mir-100-let-7a-2-mir-125b-1 cluster host gene	1,547	Other	other
147	CTNNAL1	catenin alpha like 1	1,548	Plasma Membrane	other
148	KLF13	Kruppel like factor 13	1,548	Nucleus	transcription regulator
149	LURAP1L	leucine rich adaptor protein 1 like	1,548	Other	other
150	RPL22	ribosomal protein L22	1,551	Nucleus	other
151	RAP1GDS1	Rap1 GTPase-GDP dissociation stimulator 1	1,551	Cytoplasm	other

152	ETS1	ETS proto-oncogene 1, transcription factor	1,555	Nucleus	transcription regulator
153	GDF15	growth differentiation factor 15	1,556	Extracellular Space	growth factor
154	CEBPG	CCAAT/enhancer binding protein gamma	1,556	Nucleus	transcription regulator
155	UBASH3B	ubiquitin associated and SH3 domain containing B	1,557	Other	enzyme
156	ERO1A	endoplasmic reticulum oxidoreductase 1 alpha	1,559	Cytoplasm	enzyme
157	KDELC2	KDEL motif containing 2	1,561	Other	other
158	FAR2	fatty acyl-CoA reductase 2	1,562	Cytoplasm	enzyme
159	FERMT1	fermitin family member 1	1,563	Plasma Membrane	other
160	FRMD6	FERM domain containing 6	1,565	Cytoplasm	other
161	LAMA4	laminin subunit alpha 4	1,567	Extracellular Space	enzyme
162	SOCS2	suppressor of cytokine signaling 2	1,568	Cytoplasm	other
163	SEC23B	Sec23 homolog B, coat complex II component	1,57	Extracellular Space	transporter
164	PRRC2C	proline rich coiled-coil 2C	1,571	Cytoplasm	other
165	TFB2M	transcription factor B2, mitochondrial	1,572	Cytoplasm	enzyme
166	TCEA1	transcription elongation factor A1	1,572	Nucleus	transcription regulator
167	ESAM	endothelial cell adhesion molecule	1,574	Plasma Membrane	other
168	FAM86B1	family with sequence similarity 86 member B1	1,575	Other	other
169	TPST2	tyrosylprotein sulfotransferase 2	1,575	Cytoplasm	enzyme
170	KDSR	3-ketodihydrosphingosine reductase	1,577	Plasma Membrane	enzyme
171	GNAI1	G protein subunit alpha i1	1,578	Plasma Membrane	enzyme
172	AKAP12	A-kinase anchoring protein 12	1,578	Cytoplasm	transporter
173	ELL2	elongation factor for RNA polymerase II 2	1,58	Nucleus	transcription regulator
174	HNRNPF	heterogeneous nuclear ribonucleoprotein F	1,582	Nucleus	other
175	CDV3	CDV3 homolog	1,584	Cytoplasm	other
176	RPS27AP11	ribosomal protein S27a pseudogene 11	1,585	Other	other
177	USP15	ubiquitin specific peptidase 15	1,589	Cytoplasm	peptidase
178	AFAP1L1	actin filament associated protein 1 like 1	1,591	Other	other
179	STK39	serine/threonine kinase 39	1,598	Nucleus	kinase

180	CALD1	caldesmon 1	1,599	Cytoplasm	other
181	MSH2	mutS homolog 2	1,603	Nucleus	enzyme
182	RTN4	reticulon 4	1,603	Cytoplasm	other
183	NPM1P28	nucleophosmin 1 pseudogene 28	1,606	Other	other
184	MYO19	myosin XIX	1,609	Cytoplasm	enzyme
185	KANK1	KN motif and ankyrin repeat domains 1	1,611	Nucleus	transcription regulator
186	ANKRD20A4 (includes others)	ankyrin repeat domain 20 family member A4	1,612	Plasma Membrane	other
187	PTGFRN	prostaglandin F2 receptor inhibitor	1,613	Plasma Membrane	other
188	FLNB	filamin B	1,614	Cytoplasm	other
189	CBFB	core-binding factor beta subunit	1,616	Nucleus	transcription regulator
190	IMPA1	inositol monophosphatase 1	1,625	Cytoplasm	phosphatase
191	MAMLD1	mastermind like domain containing 1	1,627	Nucleus	other
192	WLS	wntless Wnt ligand secretion mediator	1,627	Cytoplasm	other
193	ARMCX1	armadillo repeat containing, X-linked 1	1,633	Other	other
194	BZW1P2	basic leucine zipper and W2 domains 1 pseudogene 2	1,636	Other	other
195	LAMB3	laminin subunit beta 3	1,64	Extracellular Space	transporter
196	LOX	lysyl oxidase	1,64	Extracellular Space	enzyme
197	INTS13	integrator complex subunit 13	1,644	Cytoplasm	other
198	FABP5P2	fatty acid binding protein 5 pseudogene 2	1,647	Cytoplasm	other
199	XPO1	exportin 1	1,652	Nucleus	transporter
200	STEAP1B	STEAP family member 1B	1,655	Other	other
201	GARS	glycyl-tRNA synthetase	1,656	Cytoplasm	enzyme
202	FEZ2	fasciculation and elongation protein zeta 2	1,661	Cytoplasm	other
203	RNU6-15P	RNA, U6 small nuclear 15, pseudogene	1,664	Other	other
204	ARFGEF1	ADP ribosylation factor guanine nucleotide exchange factor 1	1,668	Cytoplasm	other
205	CMPK1	cytidine/uridine monophosphate kinase 1	1,673	Nucleus	kinase
206	INF2	inverted formin, FH2 and WH2 domain containing	1,677	Cytoplasm	other

207	ITGA2	integrin subunit alpha 2	1,679	Plasma Membrane	transmembrane receptor
208	RPL34	ribosomal protein L34	1,679	Cytoplasm	other
209	NACAP1	nascent polypeptide associated complex alpha subunit pseudogene 1	1,685	Other	other
210	KPNA3	karyopherin subunit alpha 3	1,697	Nucleus	transporter
211	MBTPS2	membrane bound transcription factor peptidase, site 2	1,697	Cytoplasm	peptidase
212	SLC30A9	solute carrier family 30 member 9	1,699	Nucleus	transporter
213	PEG10	paternally expressed 10	1,704	Nucleus	other
214	NT5DC1	5'-nucleotidase domain containing 1	1,704	Other	other
215	TMEM156	transmembrane protein 156	1,71	Other	other
216	IFFO1	intermediate filament family orphan 1	1,711	Other	other
217	UQCRH	ubiquinol-cytochrome c reductase hinge protein	1,716	Cytoplasm	enzyme
218	WWP1P1	WW domain containing E3 ubiquitin protein ligase 1 pseudogene 1	1,719	Other	other
219	VIM	vimentin	1,722	Cytoplasm	other
220	KHDRBS3	KH RNA binding domain containing, signal transduction associated 3	1,732	Nucleus	other
221	DSC2	desmocollin 2	1,738	Plasma Membrane	other
222	POU2F1	POU class 2 homeobox 1	1,747	Nucleus	transcription regulator
223	KLF4	Kruppel like factor 4	1,748	Nucleus	transcription regulator
224	VSNL1	visinin like 1	1,752	Cytoplasm	other
225	XRN1	5'-3' exoribonuclease 1	1,762	Cytoplasm	enzyme
226	FAM49B	family with sequence similarity 49 member B	1,765	Extracellular Space	other
227	SLCO1B3	solute carrier organic anion transporter family member 1B3	1,773	Plasma Membrane	transporter
228	LAMB1	laminin subunit beta 1	1,777	Extracellular Space	other
229	HPGD	15-hydroxyprostaglandin dehydrogenase	1,779	Cytoplasm	enzyme
230	SNORD3D	small nucleolar RNA, C/D box 3D	1,786	Other	other
231	SRPX	sushi repeat containing protein, X-linked	1,79	Cytoplasm	other

232	C1QTNF1	C1q and TNF related 1	1,794	Extracellular Space	other
233	LAT2	linker for activation of T cells family member 2	1,799	Plasma Membrane	other
234	TJP1	tight junction protein 1	1,805	Plasma Membrane	other
235	PLIN2	perilipin 2	1,806	Plasma Membrane	other
236	FABP5	fatty acid binding protein 5	1,807	Cytoplasm	transporter
237	PLEK2	pleckstrin 2	1,821	Plasma Membrane	other
238	ADAM19	ADAM metallopeptidase domain 19	1,852	Plasma Membrane	peptidase
239	DCLK1	doublecortin like kinase 1	1,892	Plasma Membrane	kinase
240	CARD11	caspase recruitment domain family member 11	1,911	Cytoplasm	kinase
241	HAS3	hyaluronan synthase 3	1,925	Plasma Membrane	enzyme
242	4-Mar	membrane associated ring-CH-type finger 4	1,944	Cytoplasm	enzyme
243	IGFBP3	insulin like growth factor binding protein 3	1,958	Extracellular Space	other
244	LAMC2	laminin subunit gamma 2	1,974	Extracellular Space	other
245	NPM1P42	nucleophosmin 1 pseudogene 42	1,993	Other	other
246	NT5E	5'-nucleotidase ecto	1,994	Plasma Membrane	phosphatase
247	TGM2	transglutaminase 2	2,017	Cytoplasm	enzyme
248	DUSP5	dual specificity phosphatase 5	2,02	Nucleus	phosphatase
249	MTND5P10	mitochondrially encoded NADH:ubiquinone oxidoreductase core subunit 5 pseudogene 10	2,097	Other	other
250	MCFD2	multiple coagulation factor deficiency 2	2,121	Cytoplasm	other
251	STC1	stanniocalcin 1	2,182	Extracellular Space	kinase
252	SPAG9	sperm associated antigen 9	2,56	Cytoplasm	other
