# Supplementary material for: Optineurin downregulation induces endoplasmic reticulum stress, chaperone-mediated autophagy, and apoptosis in pancreatic cancer cells
Source: Cell Death Discov. 2019 Aug 9;5:128. doi: 10.1038/s41420-019-0206-2 (PMC6689035; doi:10.1038/s41420-019-0206-2)
Supplement: Supplementary file 8 — Supp. Table 4 [file 41420_2019_206_MOESM8_ESM.pdf]

Supplementary Table 4: Overview of primers and probes used for qRT-PCR

**Gene name**	**Primers left**	**Primers right**	**Probe**[a)]
**OPTN**	agcaaaccattgccaagc	tttcagcatgaaaatcagaacag	85
**MAP1LC3B**	cgcaccttcgaacaaagag	ctcacccttgtatcgttctattatca	89
**CDK4**	gtgcagtcggtggtacctg	ttcgcttgtgtgggttaaaa	25
**CDK6**	gaactaggcaaagacctacttctga	ggtgggaatccaggttttct	85
**CCND1**	gtgccggccttcctagtt	ggatttagggggtgaggtg	21
**CCND3**	gggatcactggcactgaag	cctgaggctctccctgagt	68
**GAPDH**	agccacatcgctcagacac	gcccaatacgaccaaatcc	60

a) The numbers correspond to the numbers of the universal probe library (Roche, Mannheim, Germany)
