# Supplementary figures and images for: Optineurin downregulation induces endoplasmic reticulum stress, chaperone-mediated autophagy, and apoptosis in pancreatic cancer cells
Source: Cell Death Discov. 2019 Aug 9;5:128. doi: 10.1038/s41420-019-0206-2 (PMC6689035; doi:10.1038/s41420-019-0206-2)

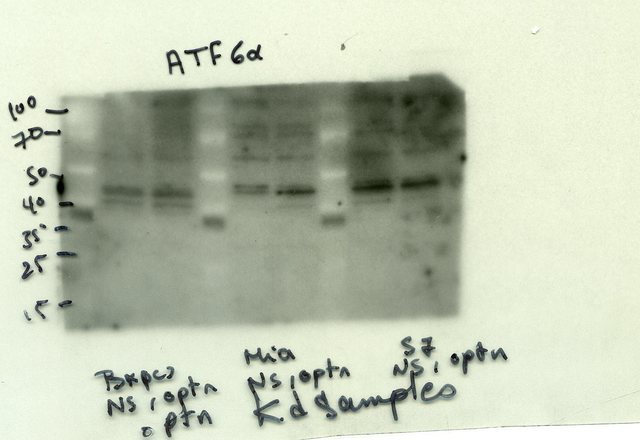

Supplement: Supplementary file 9 — WB1 [file 41420_2019_206_MOESM9_ESM.jpg]

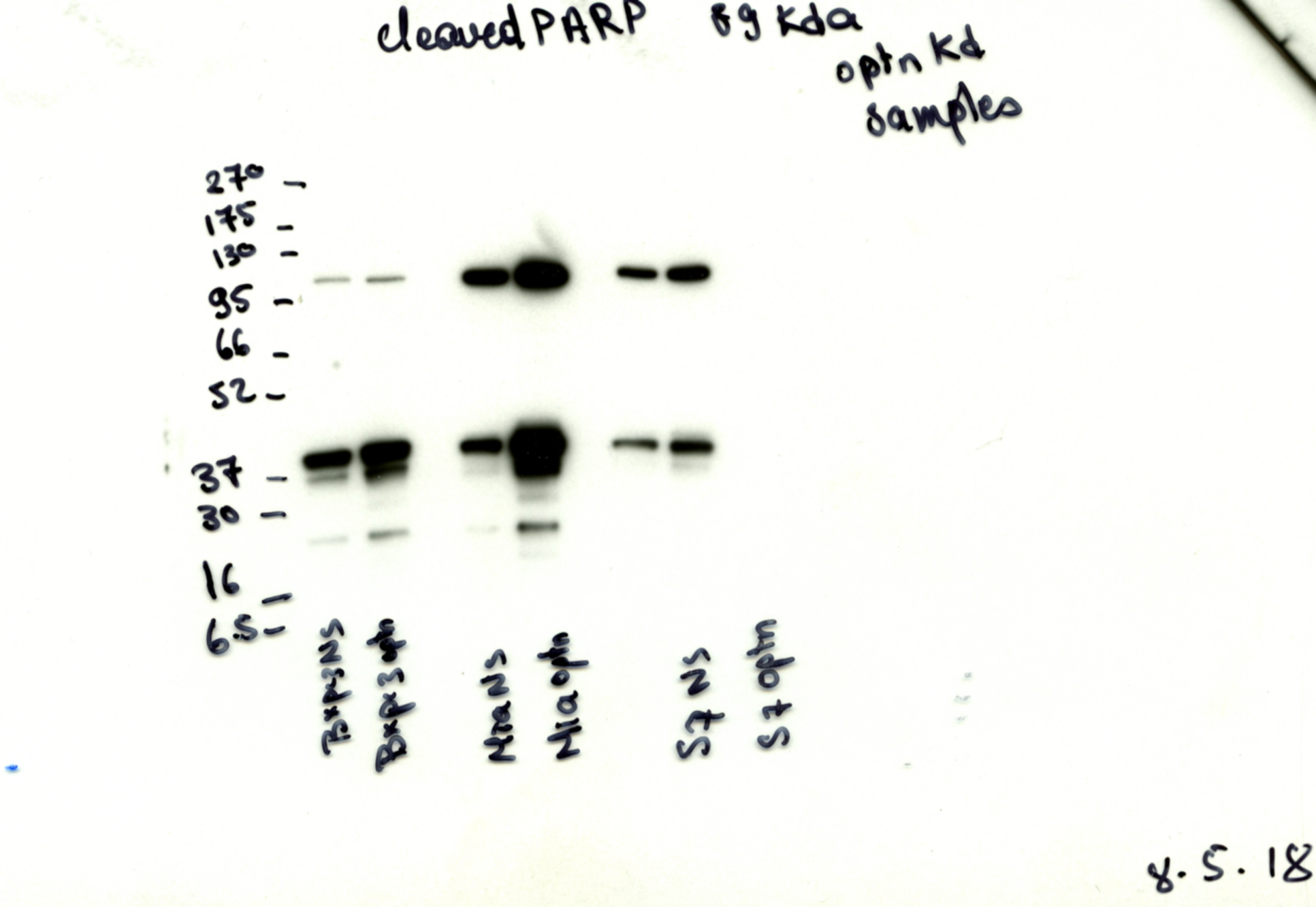

Supplement: Supplementary file 10 — WB56 [file 41420_2019_206_MOESM10_ESM.tif]

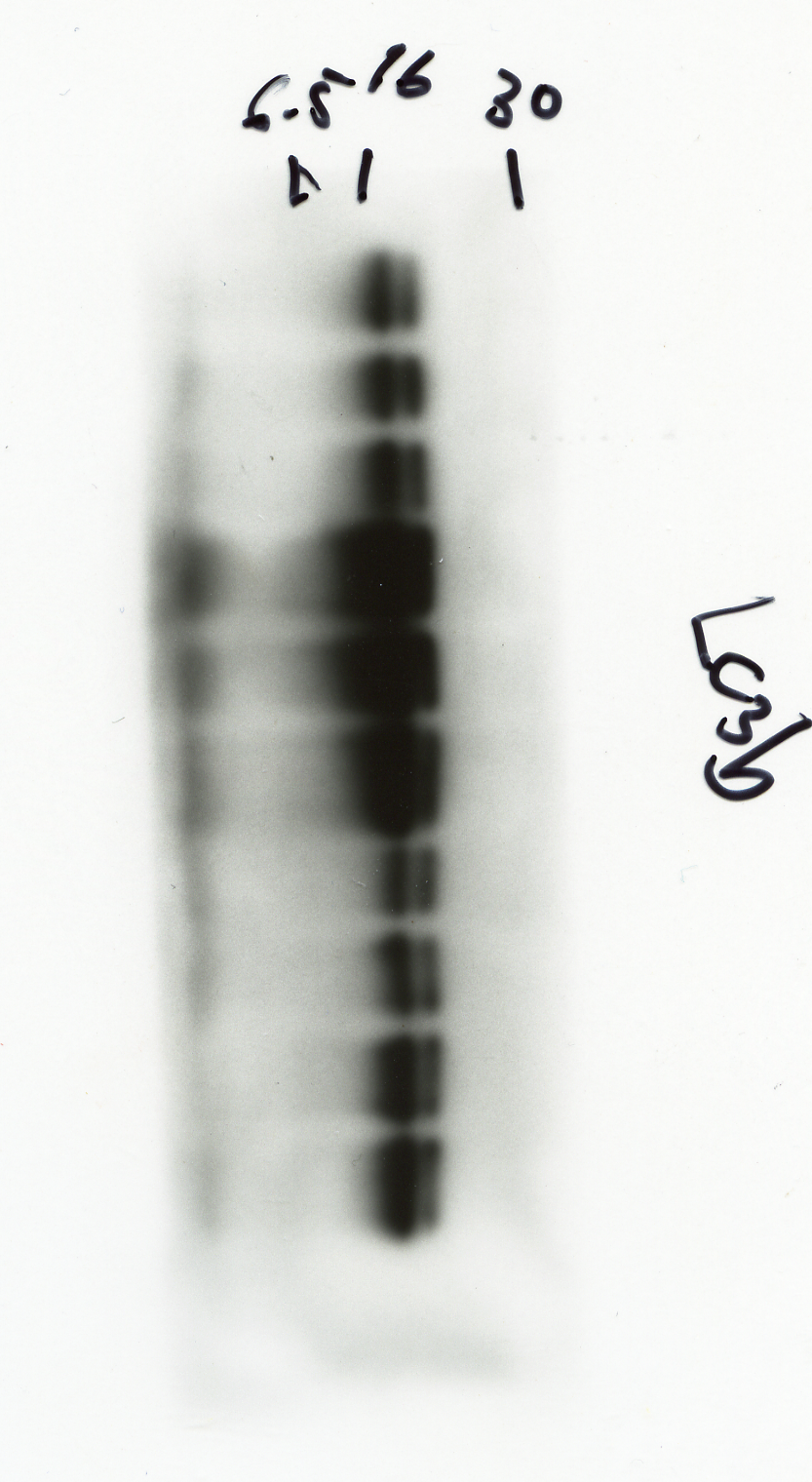

Supplement: Supplementary file 11 — WB57 [file 41420_2019_206_MOESM11_ESM.tif]

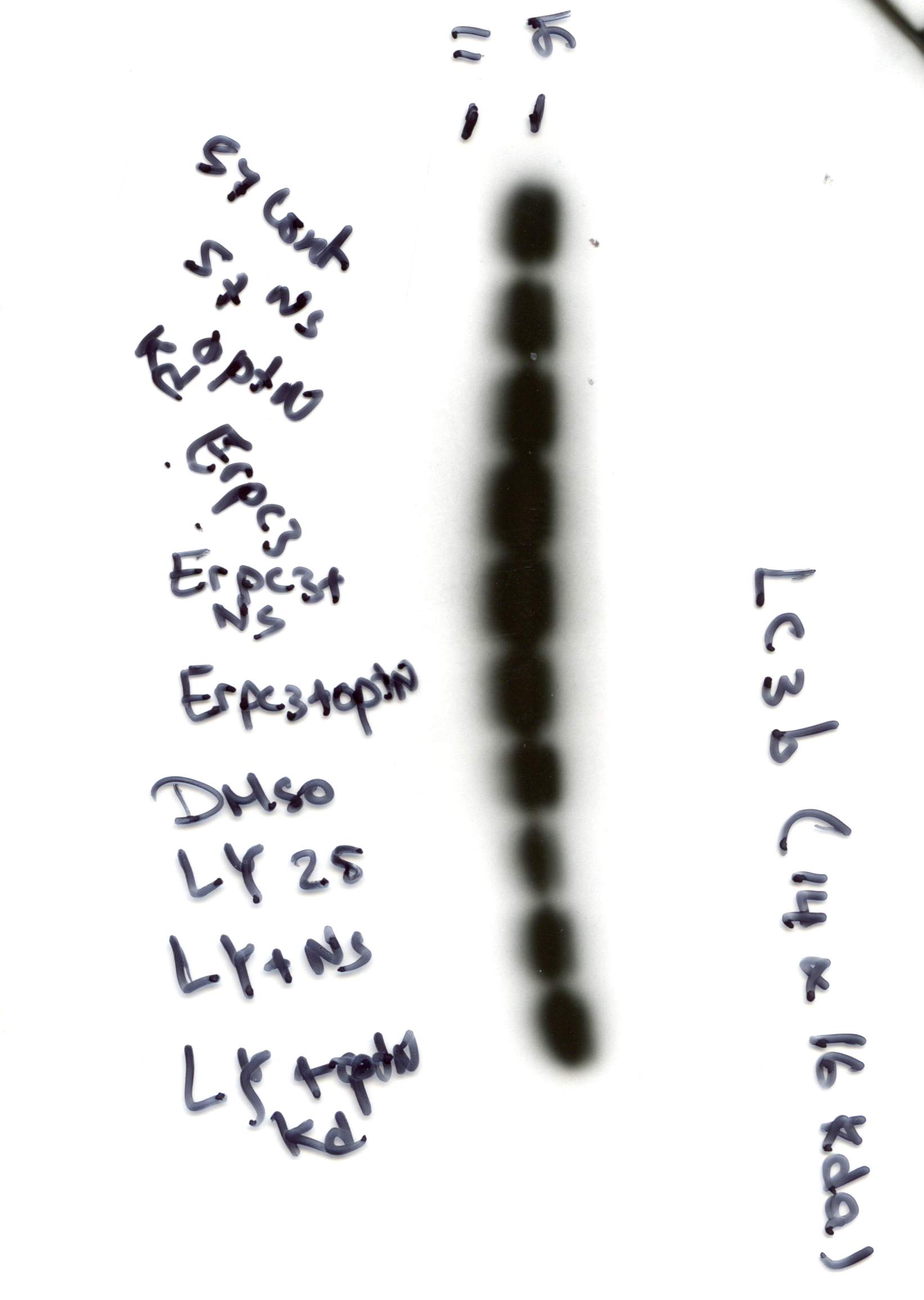

Supplement: Supplementary file 12 — WB58 [file 41420_2019_206_MOESM12_ESM.tif]

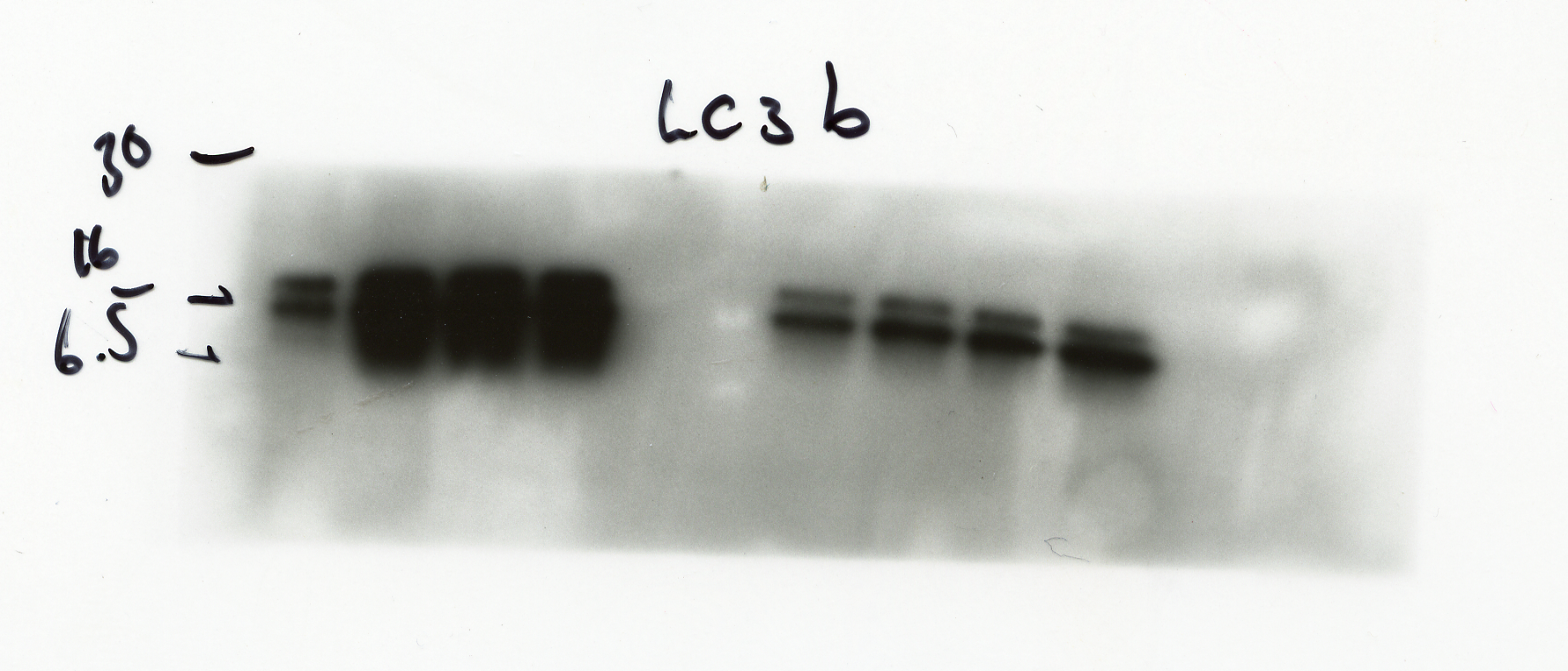

Supplement: Supplementary file 13 — WB59 [file 41420_2019_206_MOESM13_ESM.tif]

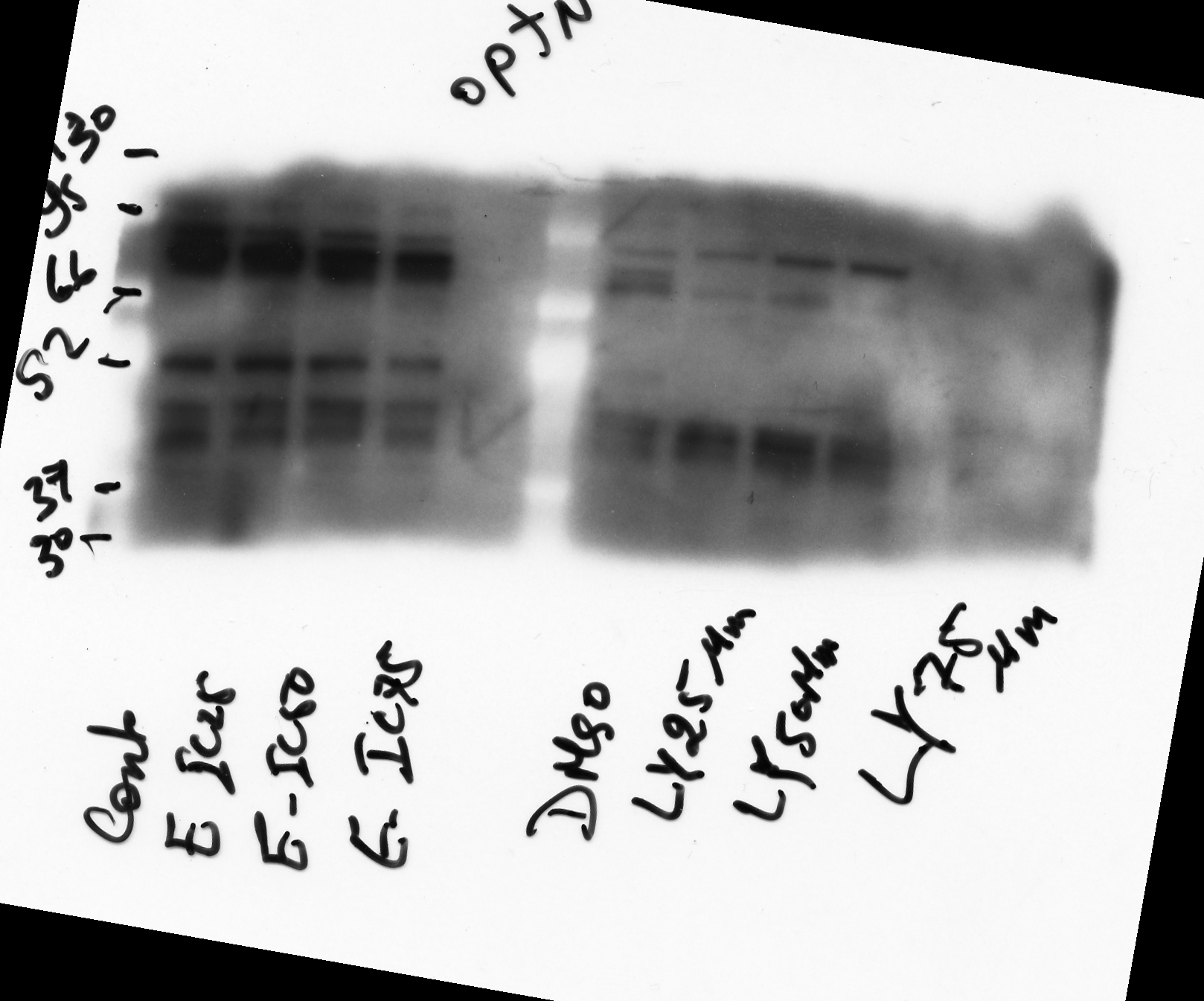

Supplement: Supplementary file 14 — WB60 [file 41420_2019_206_MOESM14_ESM.tif]

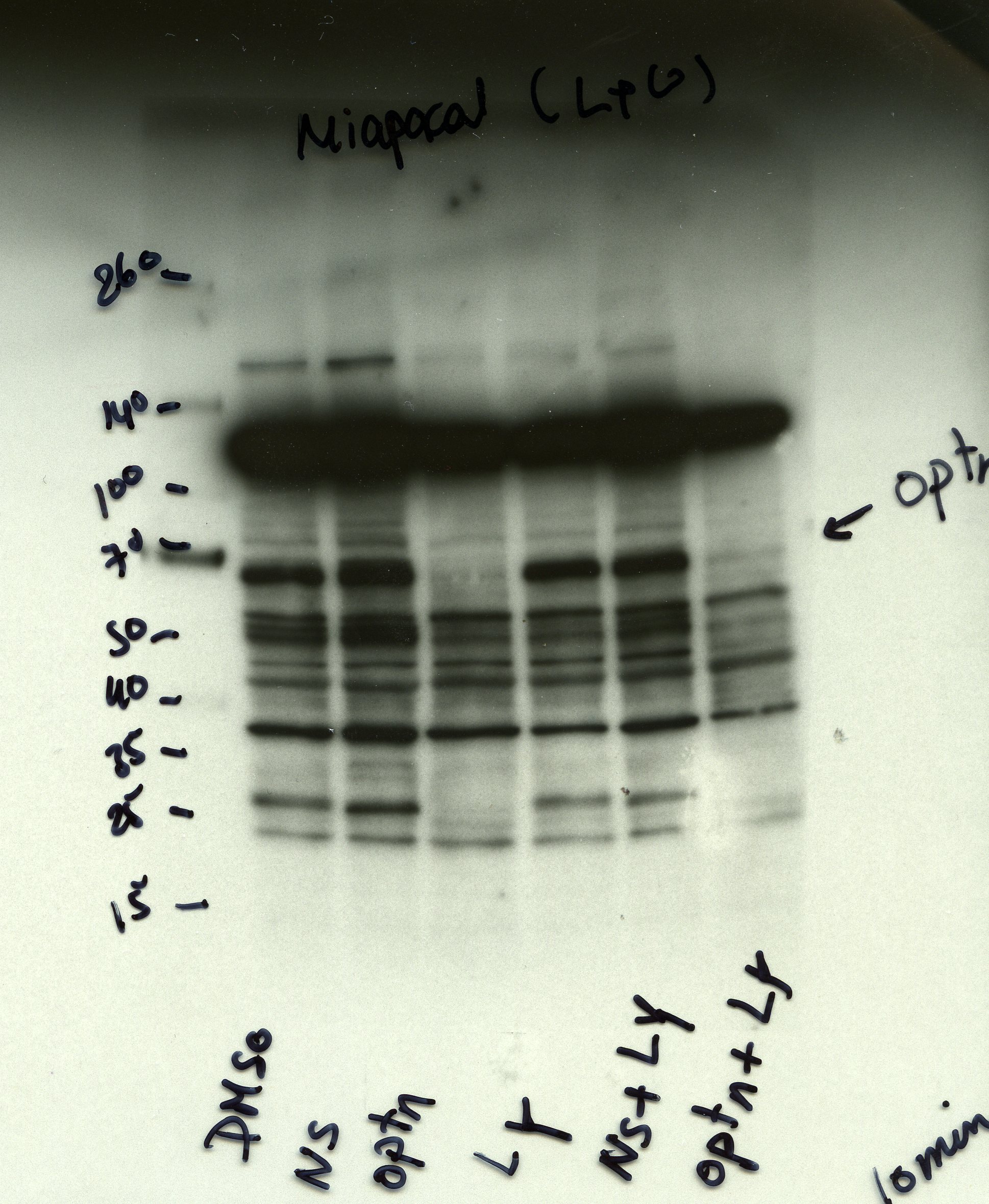

Supplement: Supplementary file 15 — WB61 [file 41420_2019_206_MOESM15_ESM.jpg]

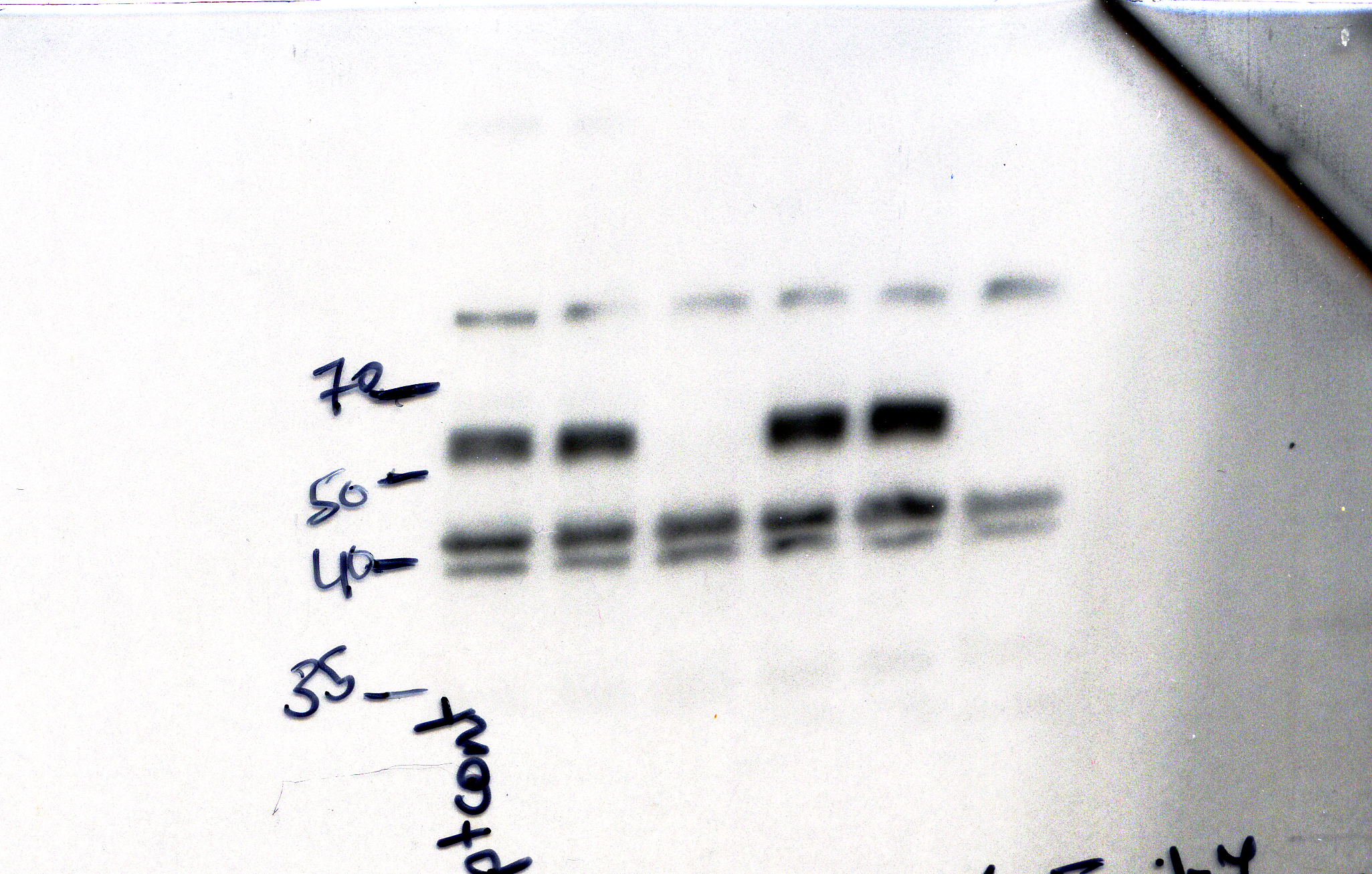

Supplement: Supplementary file 17 — WB63 [file 41420_2019_206_MOESM17_ESM.tif]

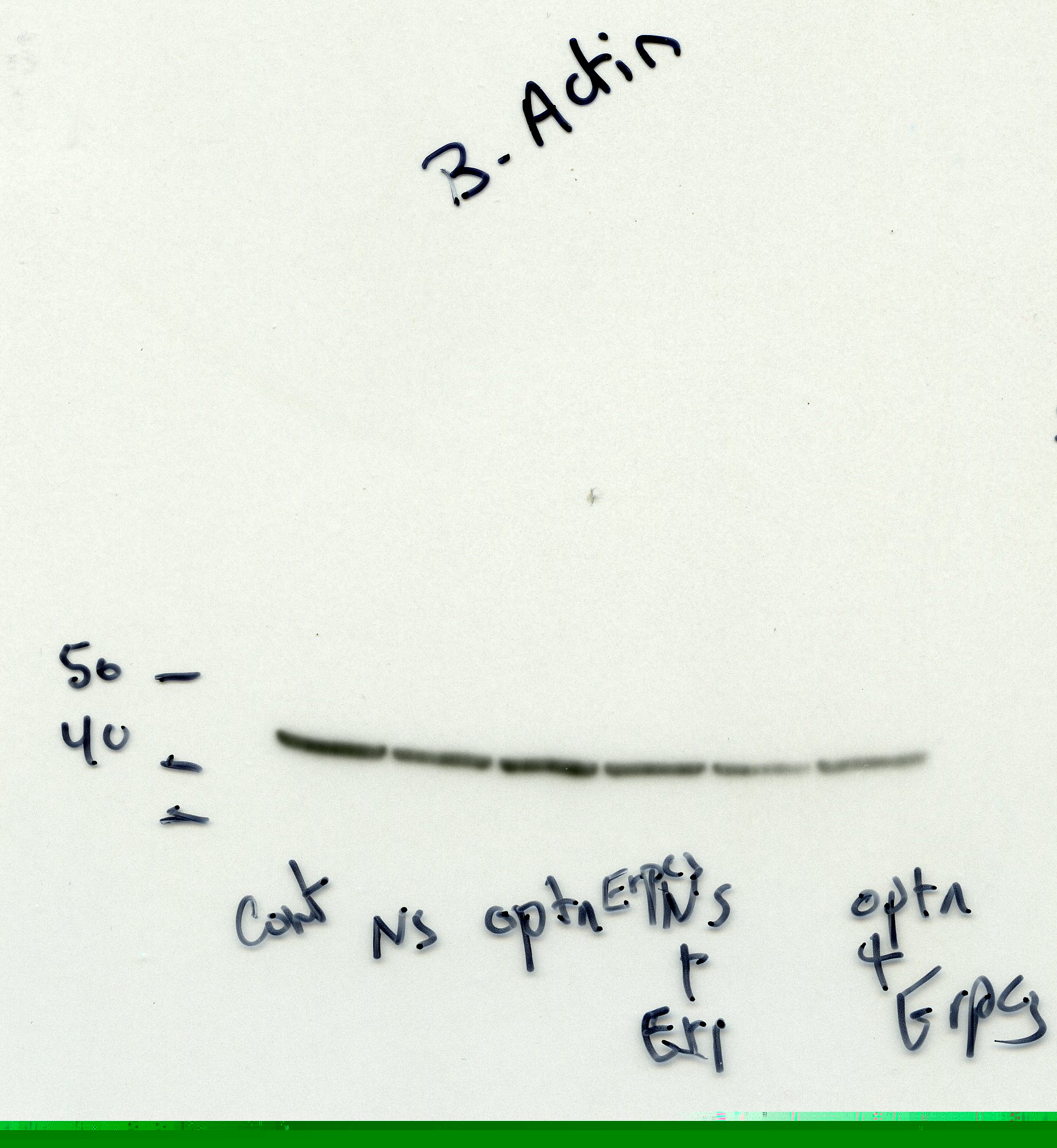

Supplement: Supplementary file 18 — WB2 [file 41420_2019_206_MOESM18_ESM.jpg]

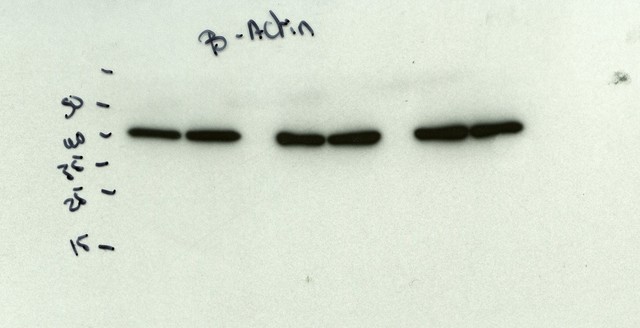

Supplement: Supplementary file 19 — WB3 [file 41420_2019_206_MOESM19_ESM.jpg]

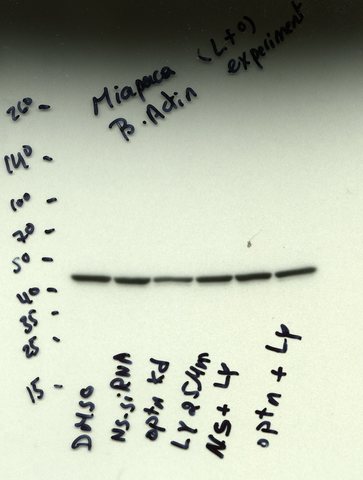

Supplement: Supplementary file 20 — WB4 [file 41420_2019_206_MOESM20_ESM.jpg]

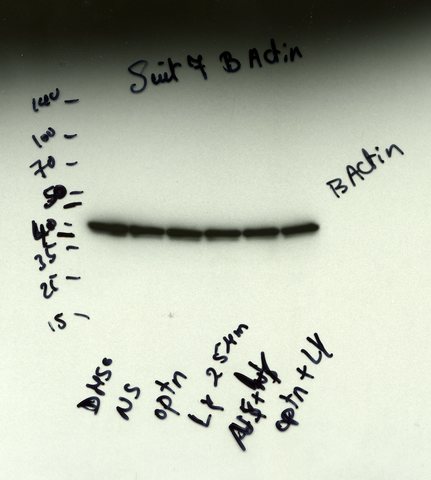

Supplement: Supplementary file 21 — WB5 [file 41420_2019_206_MOESM21_ESM.jpg]

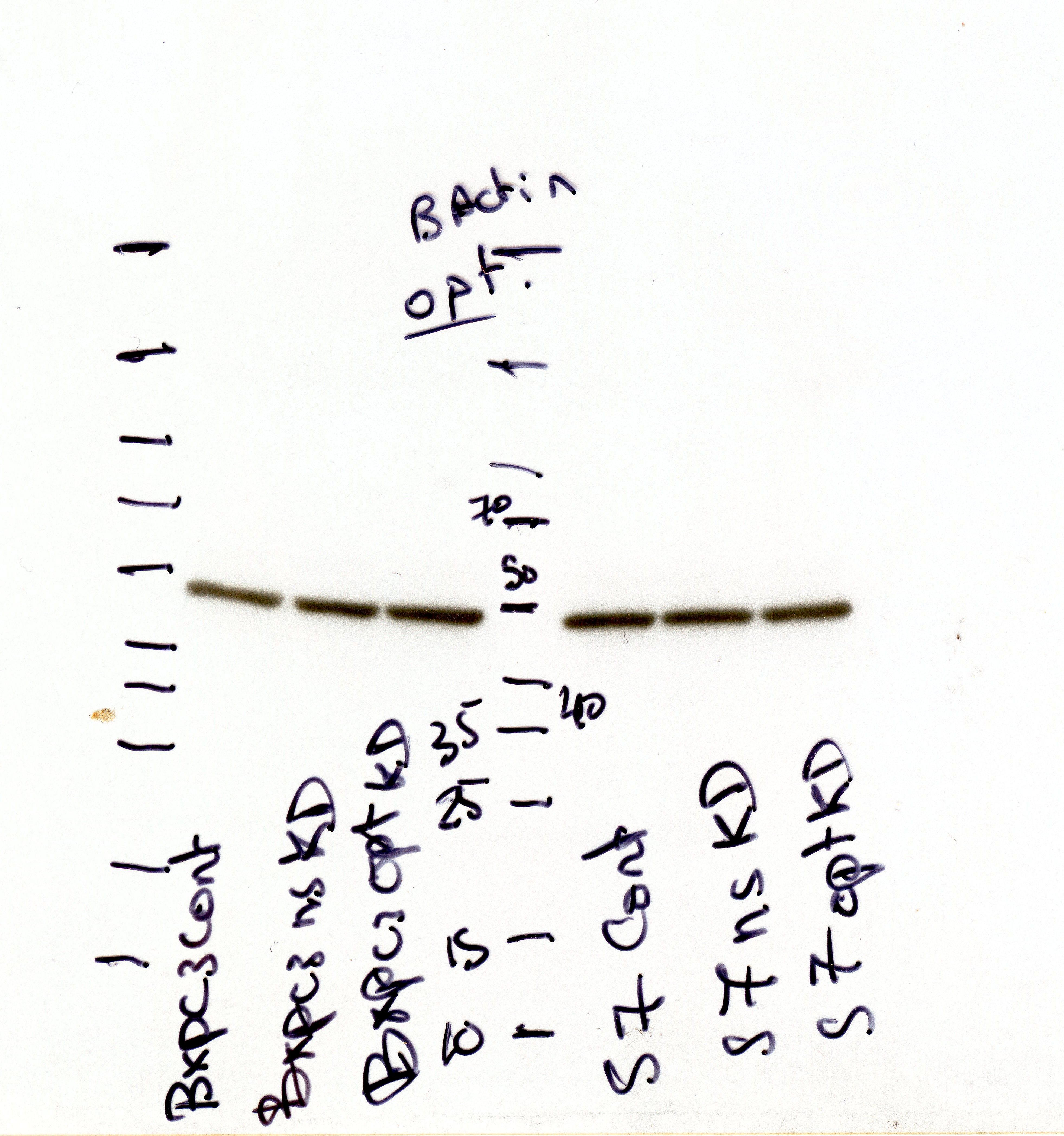

Supplement: Supplementary file 22 — WB6 [file 41420_2019_206_MOESM22_ESM.jpg]

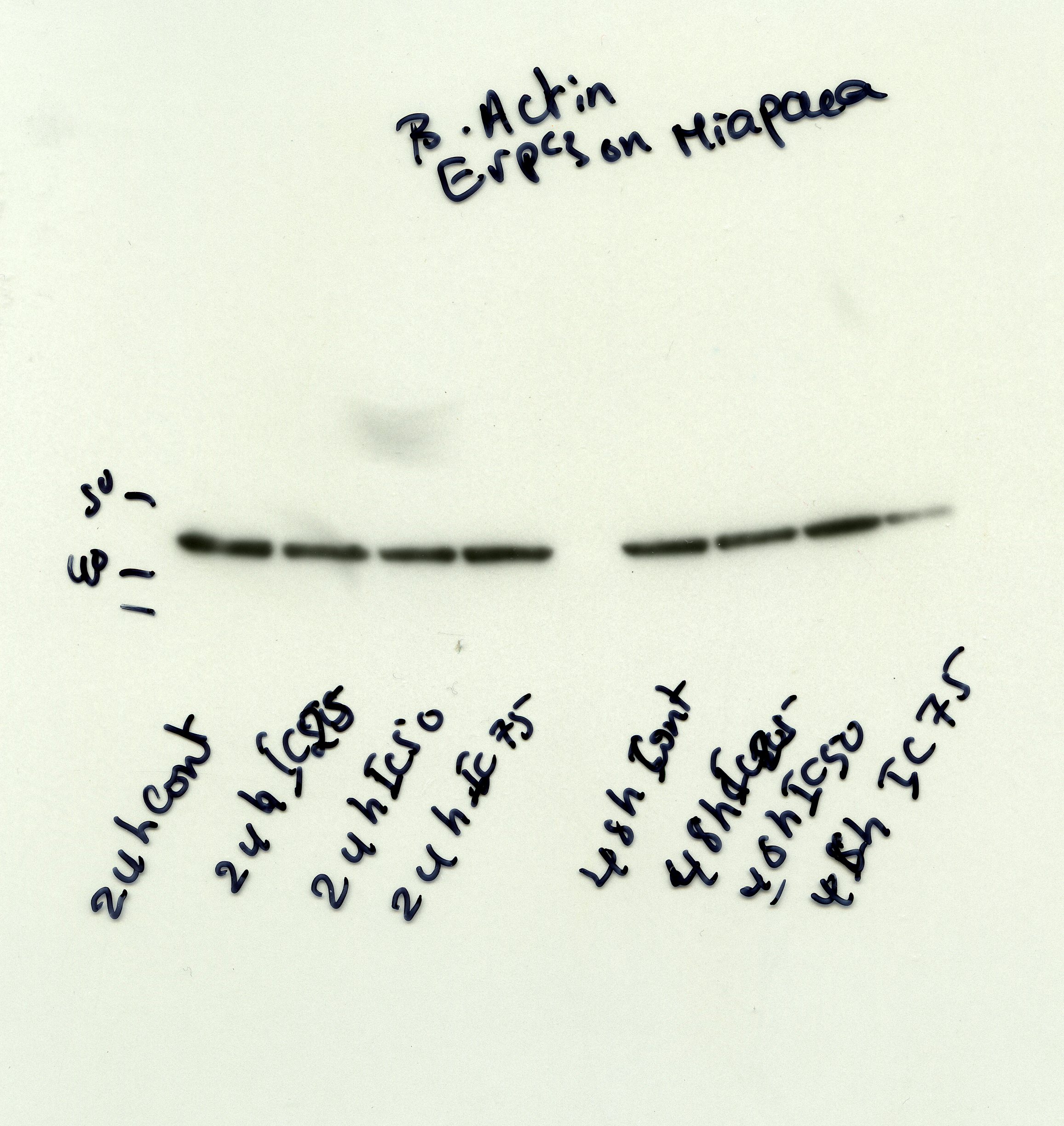

Supplement: Supplementary file 23 — WB7 [file 41420_2019_206_MOESM23_ESM.jpg]

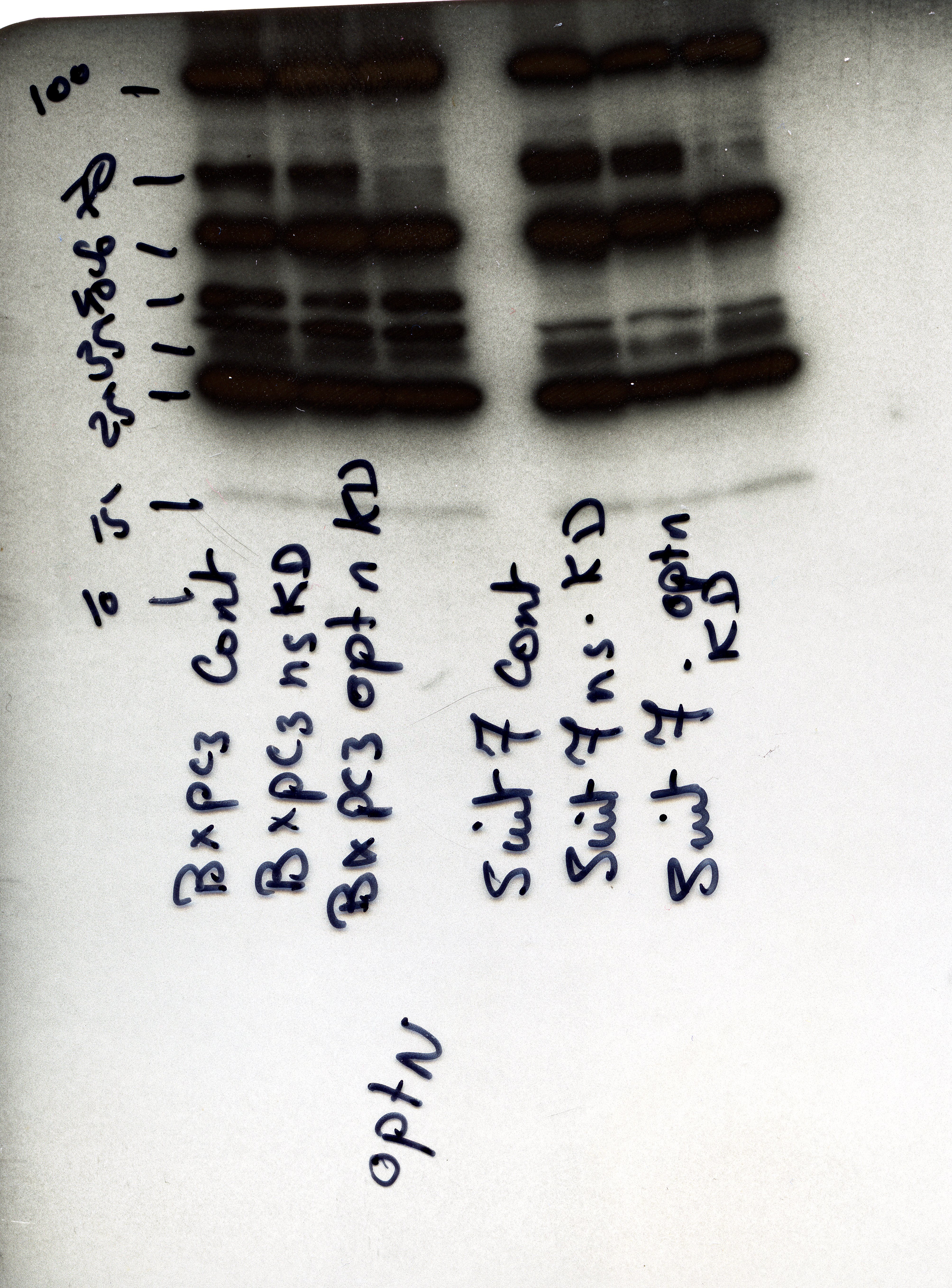

Supplement: Supplementary file 24 — WB8 [file 41420_2019_206_MOESM24_ESM.jpg]

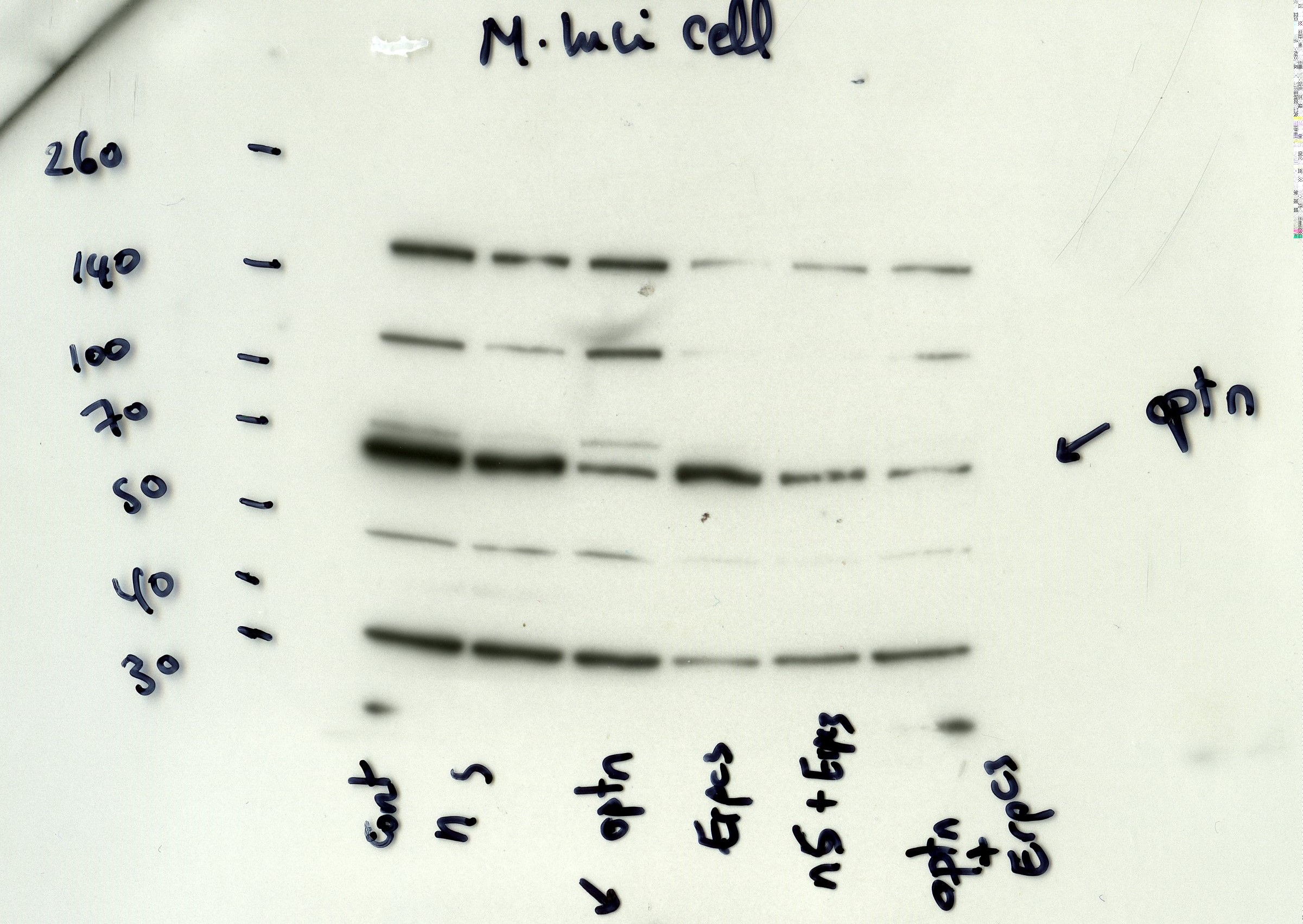

Supplement: Supplementary file 25 — WB9 [file 41420_2019_206_MOESM25_ESM.jpg]

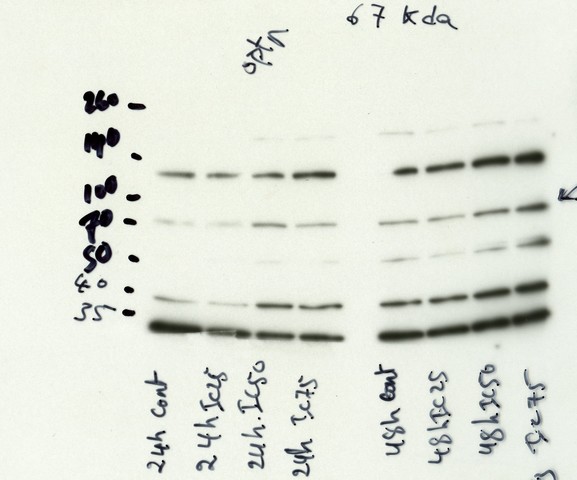

Supplement: Supplementary file 26 — WB10 [file 41420_2019_206_MOESM26_ESM.jpg]

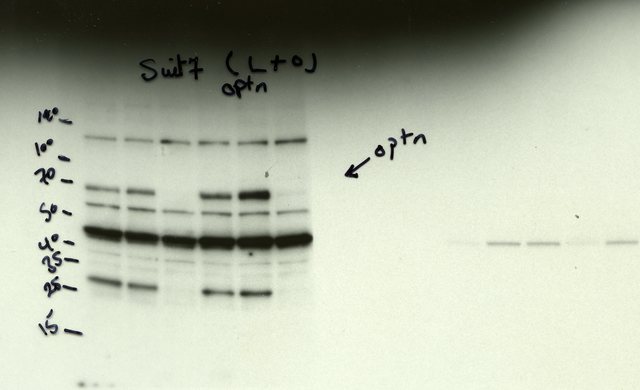

Supplement: Supplementary file 27 — WB11 [file 41420_2019_206_MOESM27_ESM.jpg]

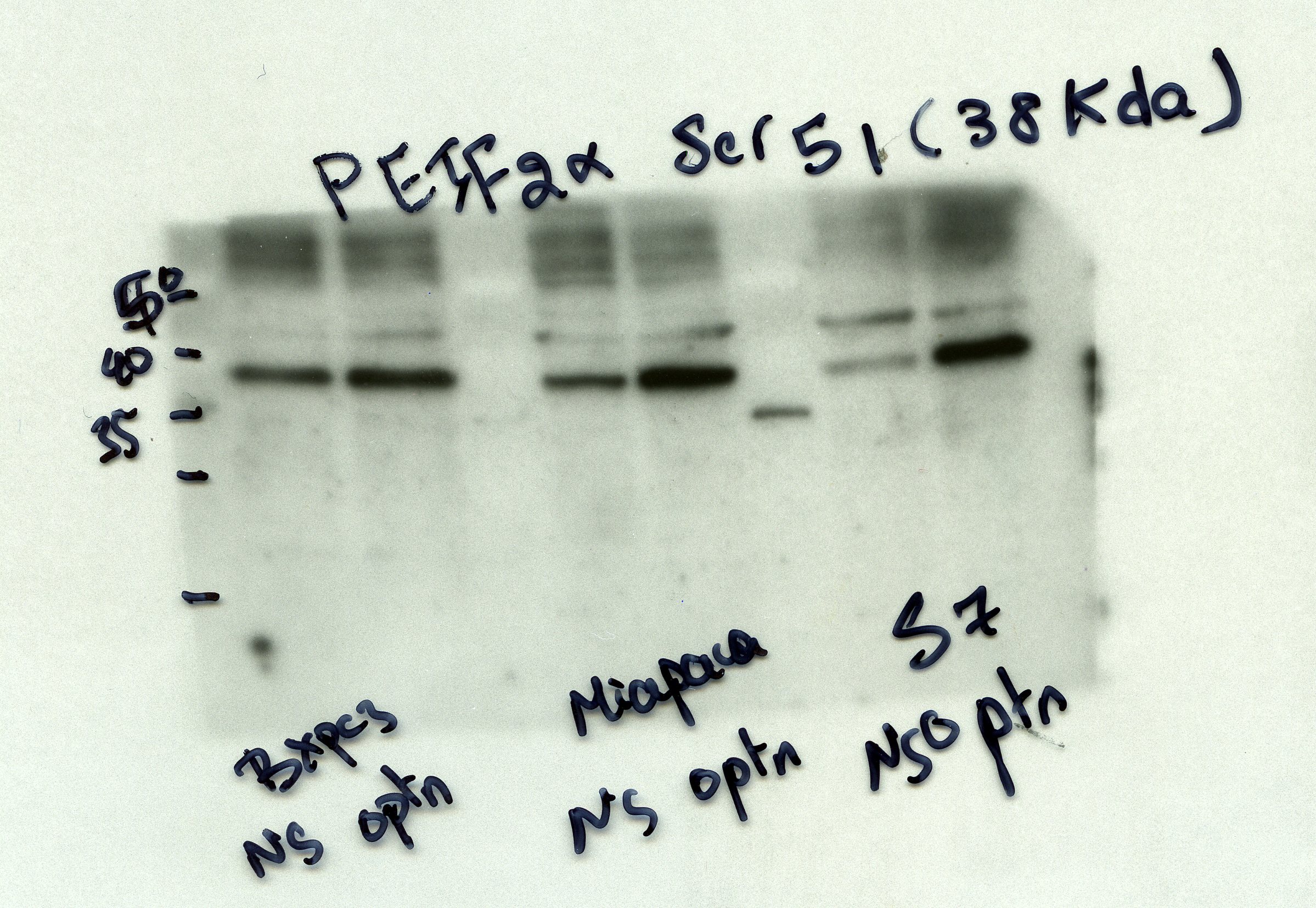

Supplement: Supplementary file 28 — WB13 [file 41420_2019_206_MOESM28_ESM.jpg]

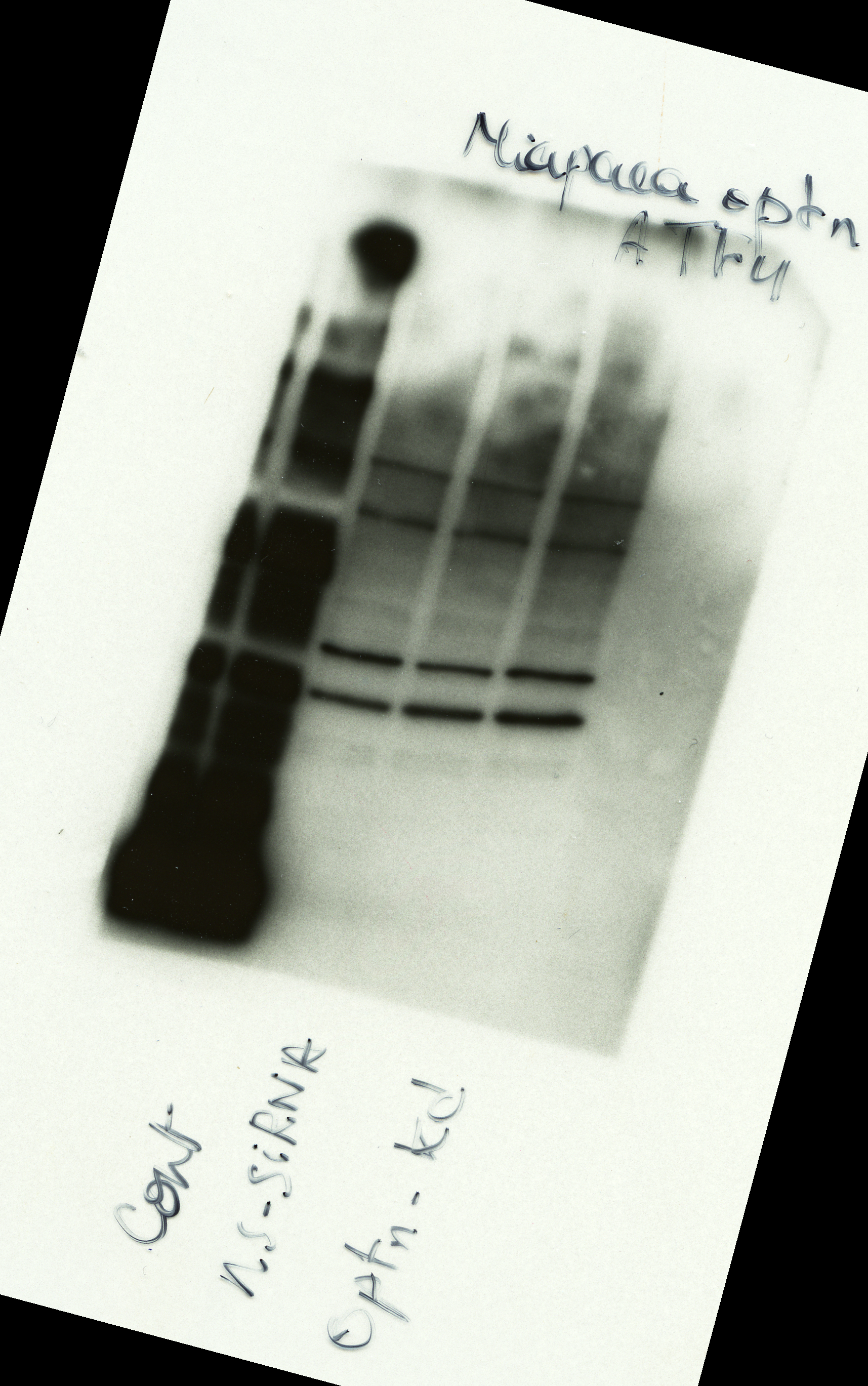

Supplement: Supplementary file 29 — WB14 [file 41420_2019_206_MOESM29_ESM.tif]

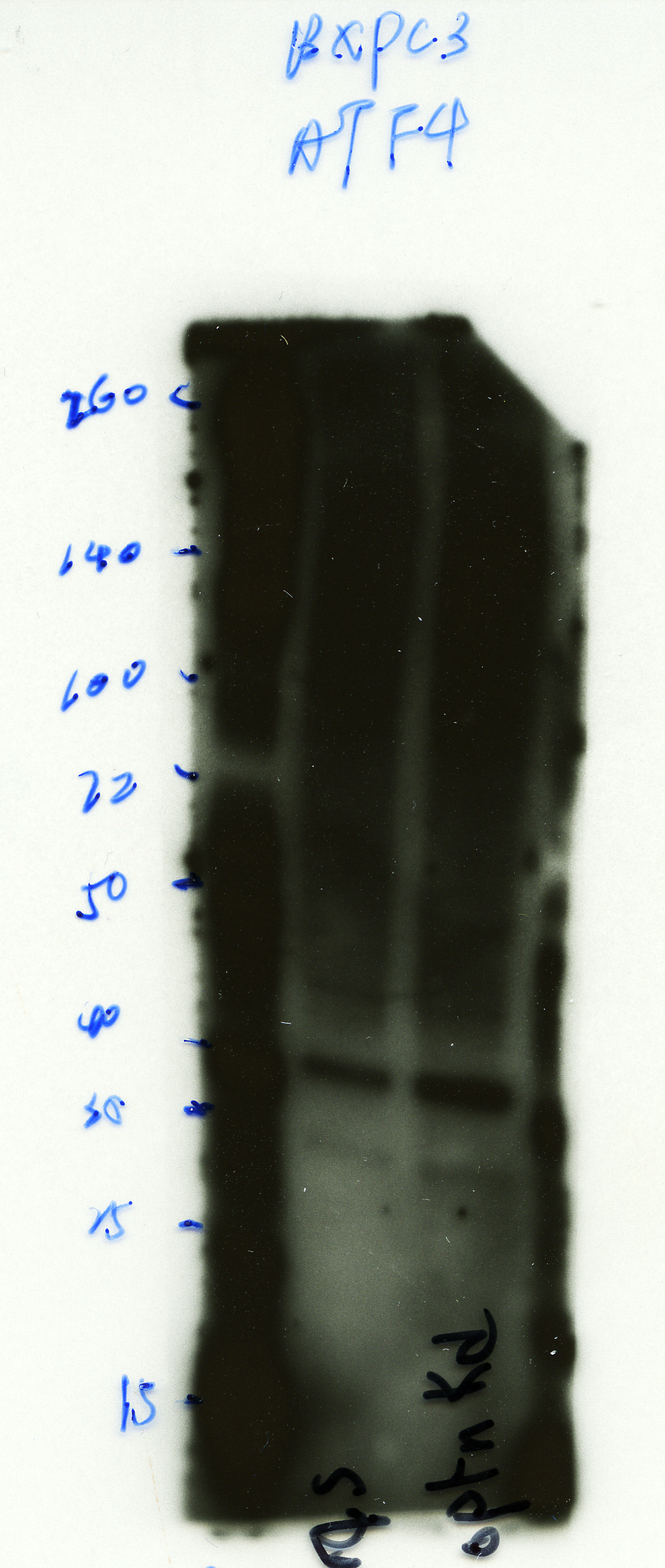

Supplement: Supplementary file 30 — WB15 [file 41420_2019_206_MOESM30_ESM.tif]

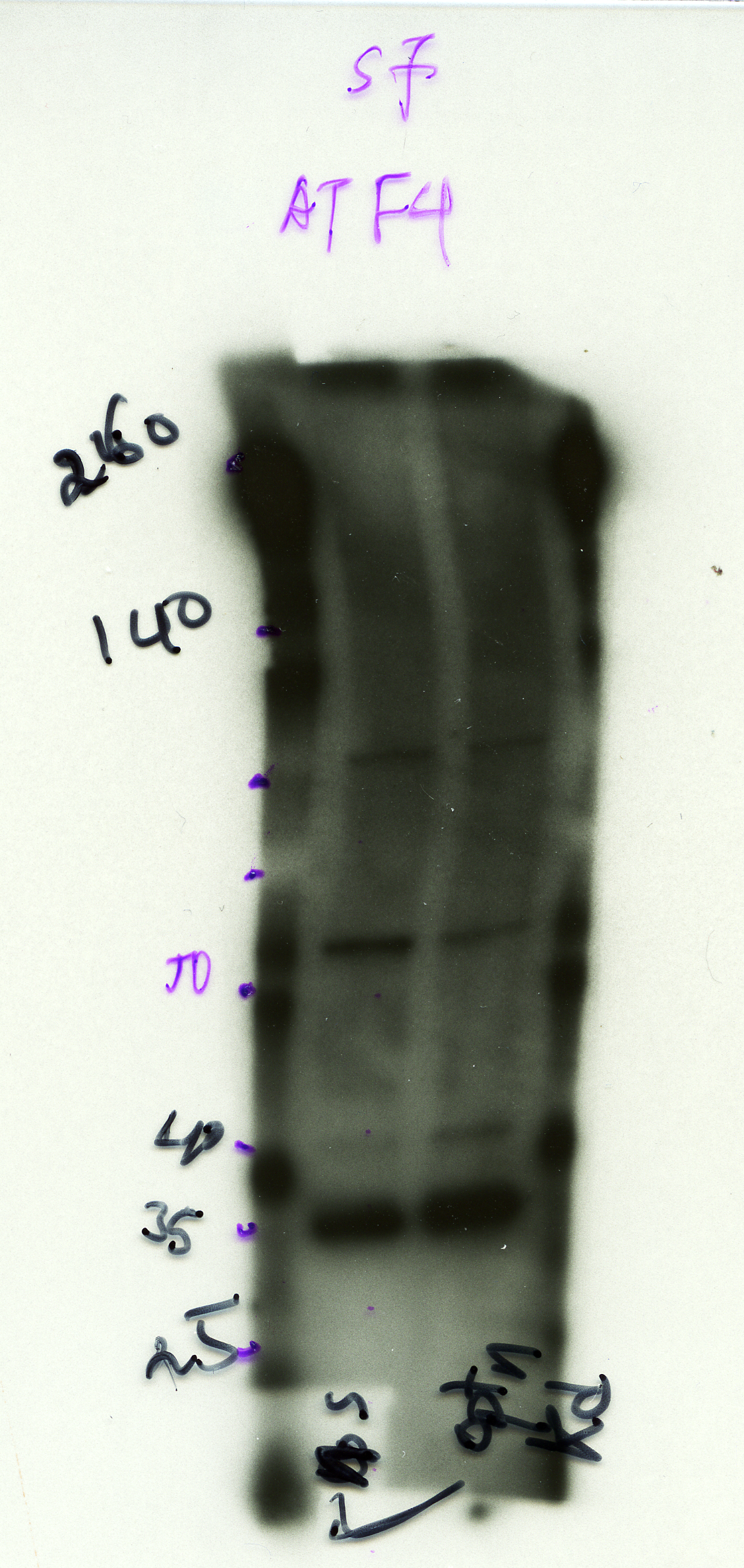

Supplement: Supplementary file 31 — WB16 [file 41420_2019_206_MOESM31_ESM.tif]

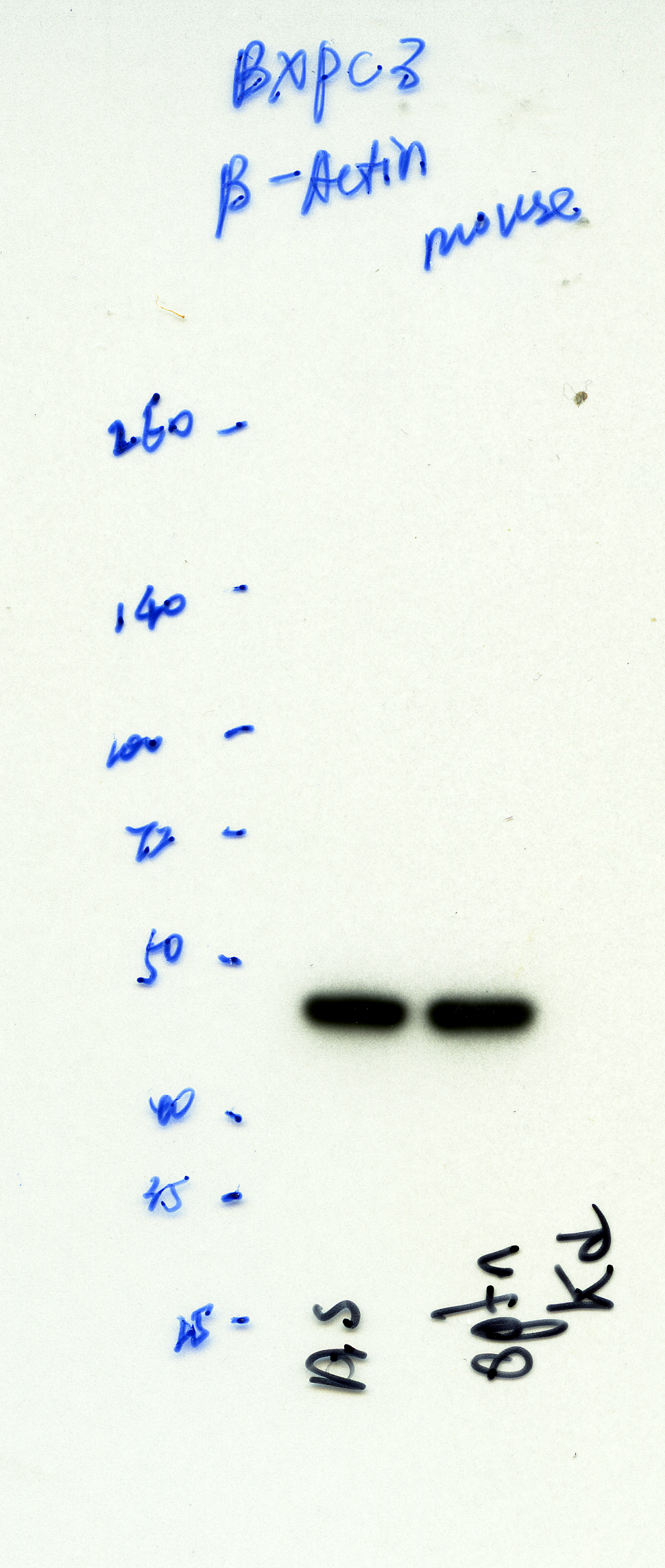

Supplement: Supplementary file 32 — WB18 [file 41420_2019_206_MOESM32_ESM.tif]

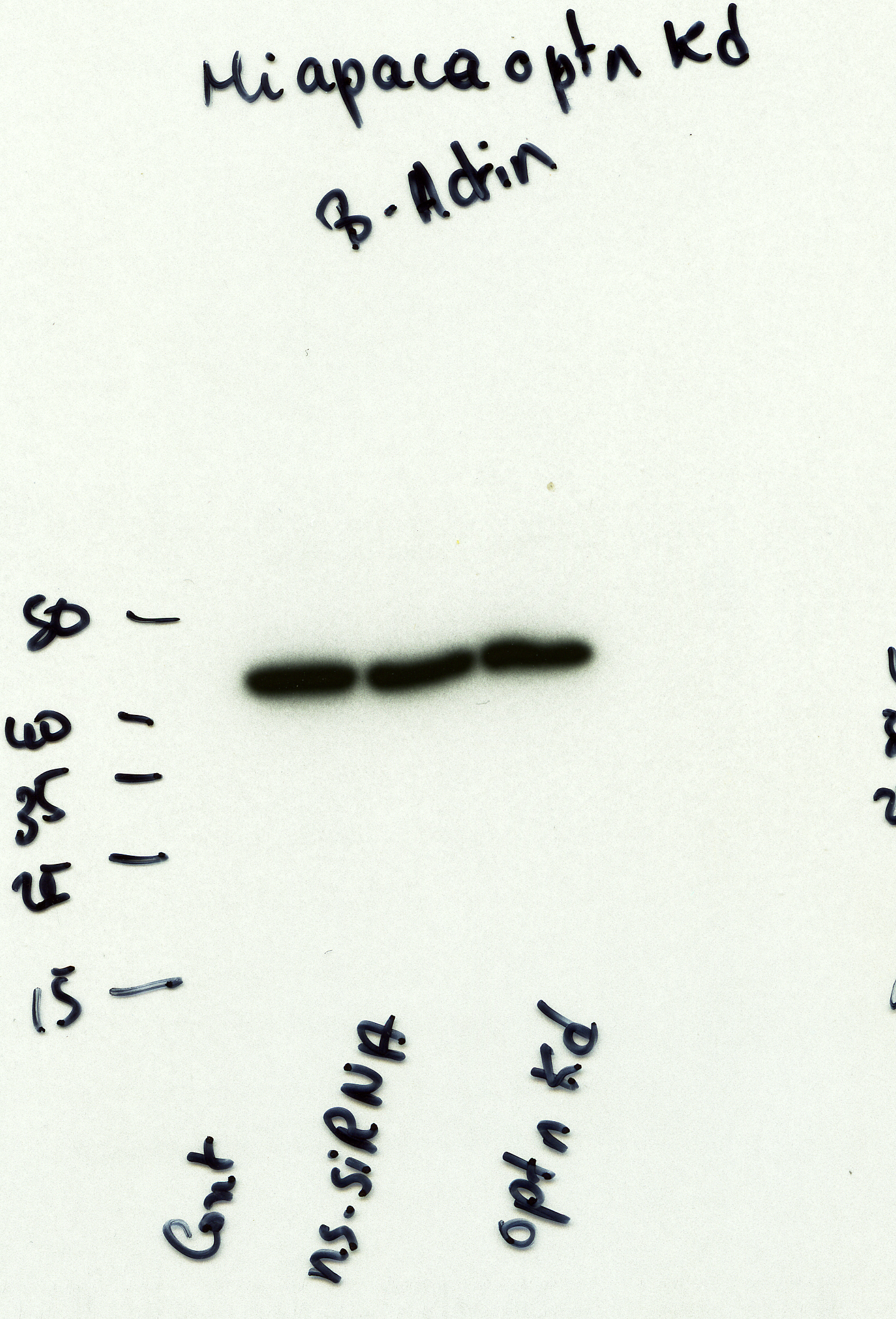

Supplement: Supplementary file 33 — WB19 [file 41420_2019_206_MOESM33_ESM.tif]

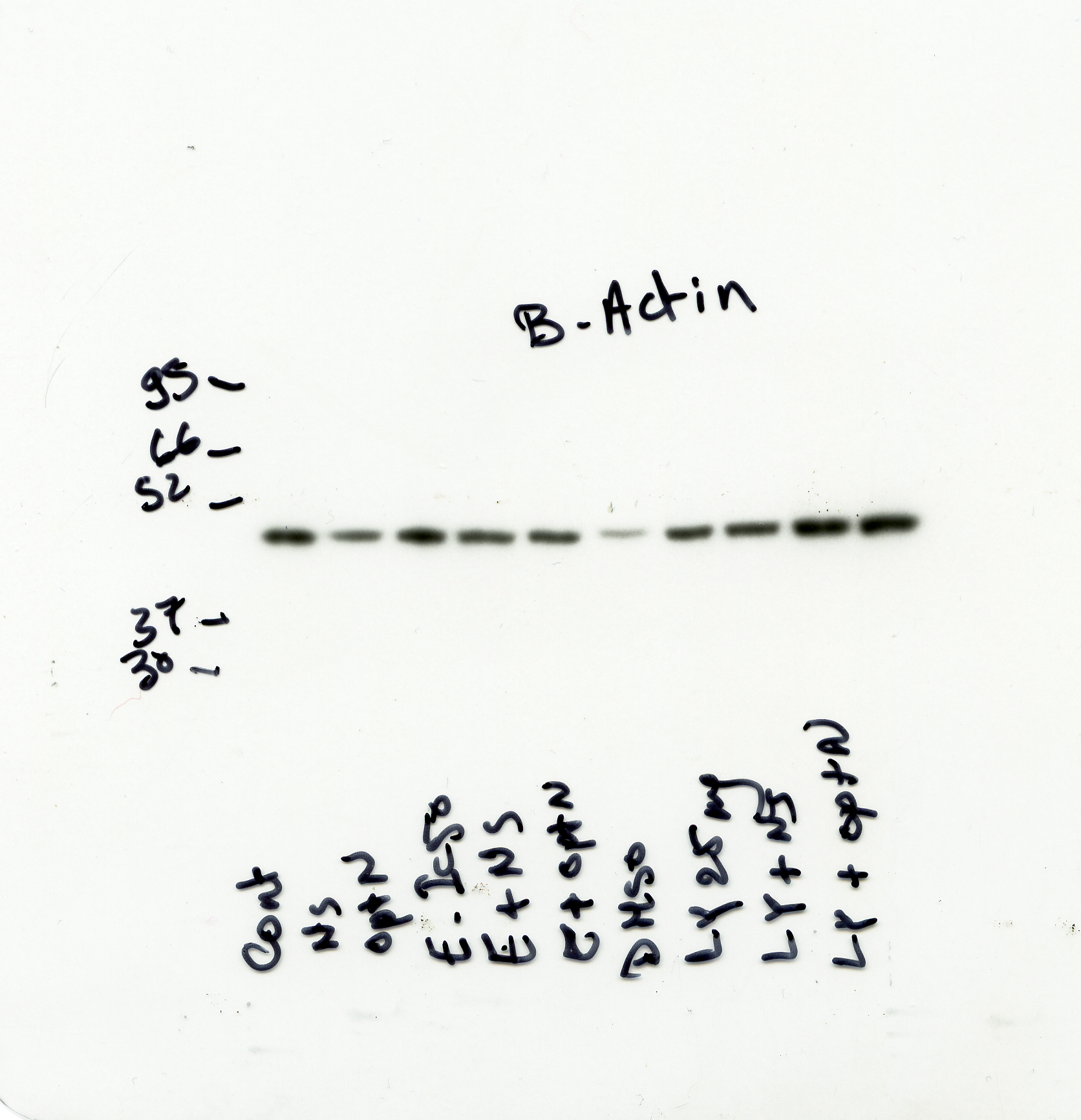

Supplement: Supplementary file 34 — WB20 [file 41420_2019_206_MOESM34_ESM.tif]

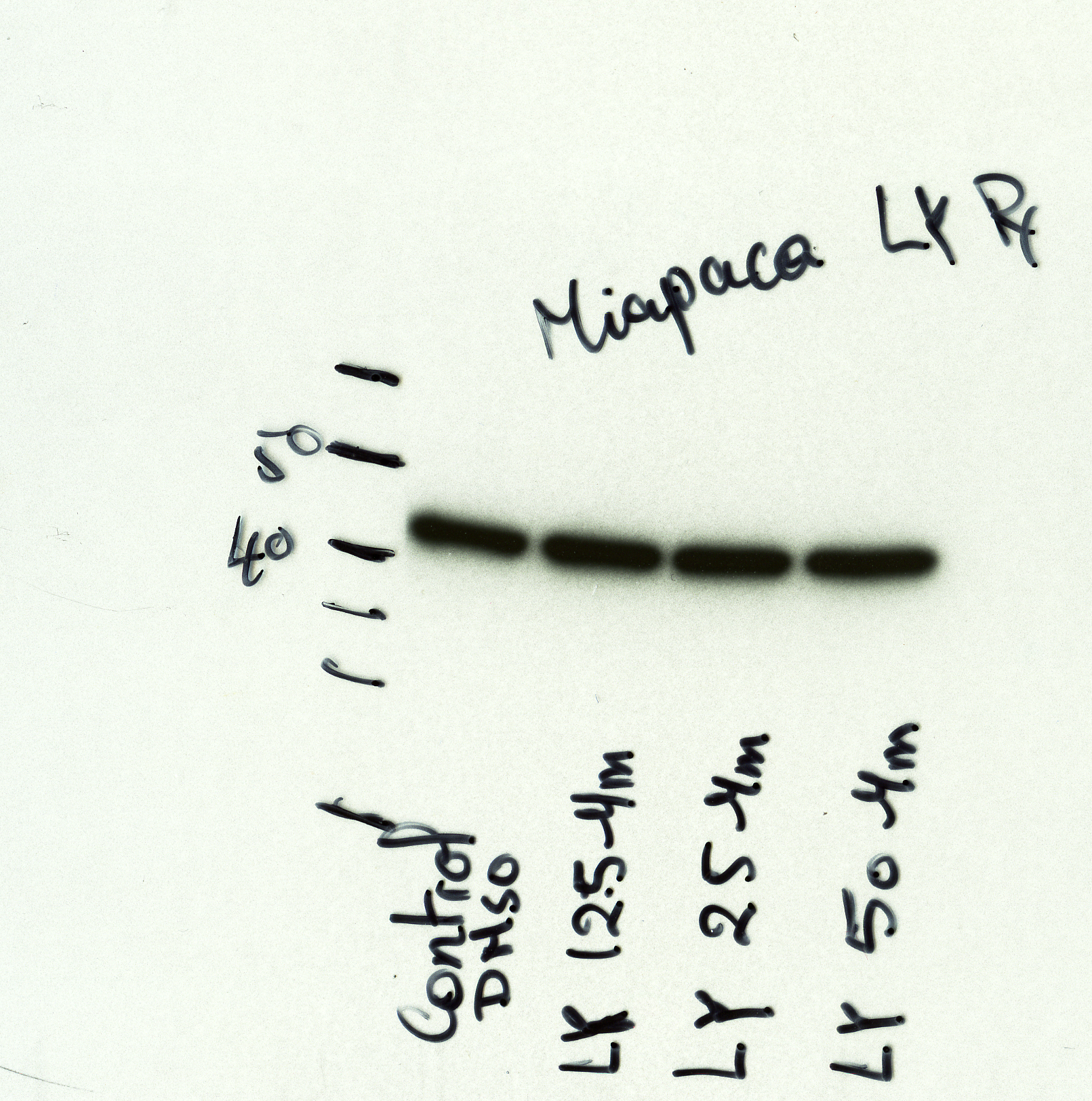

Supplement: Supplementary file 35 — WB21 [file 41420_2019_206_MOESM35_ESM.tif]

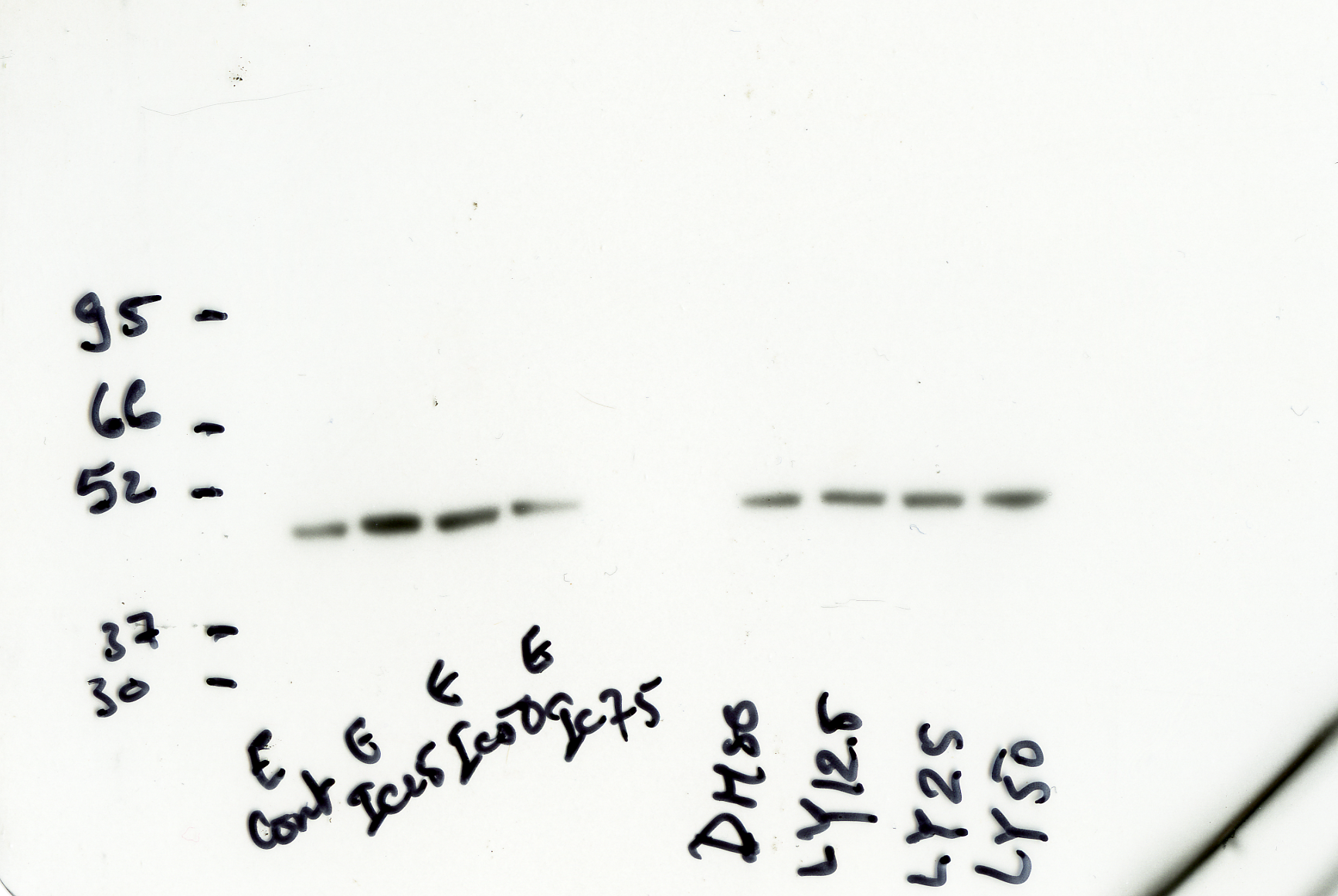

Supplement: Supplementary file 36 — WB22 [file 41420_2019_206_MOESM36_ESM.tif]

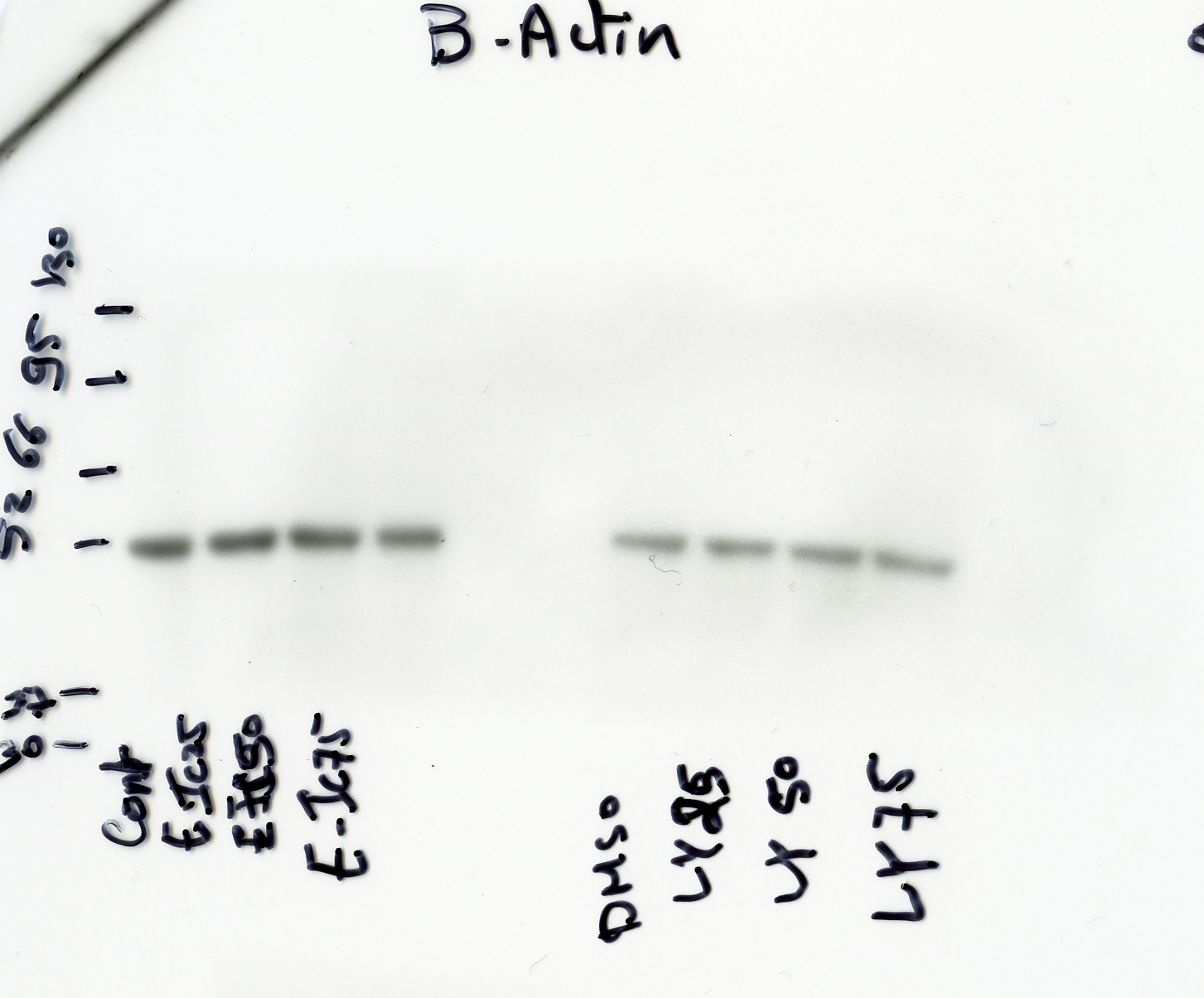

Supplement: Supplementary file 37 — WB23 [file 41420_2019_206_MOESM37_ESM.tif]

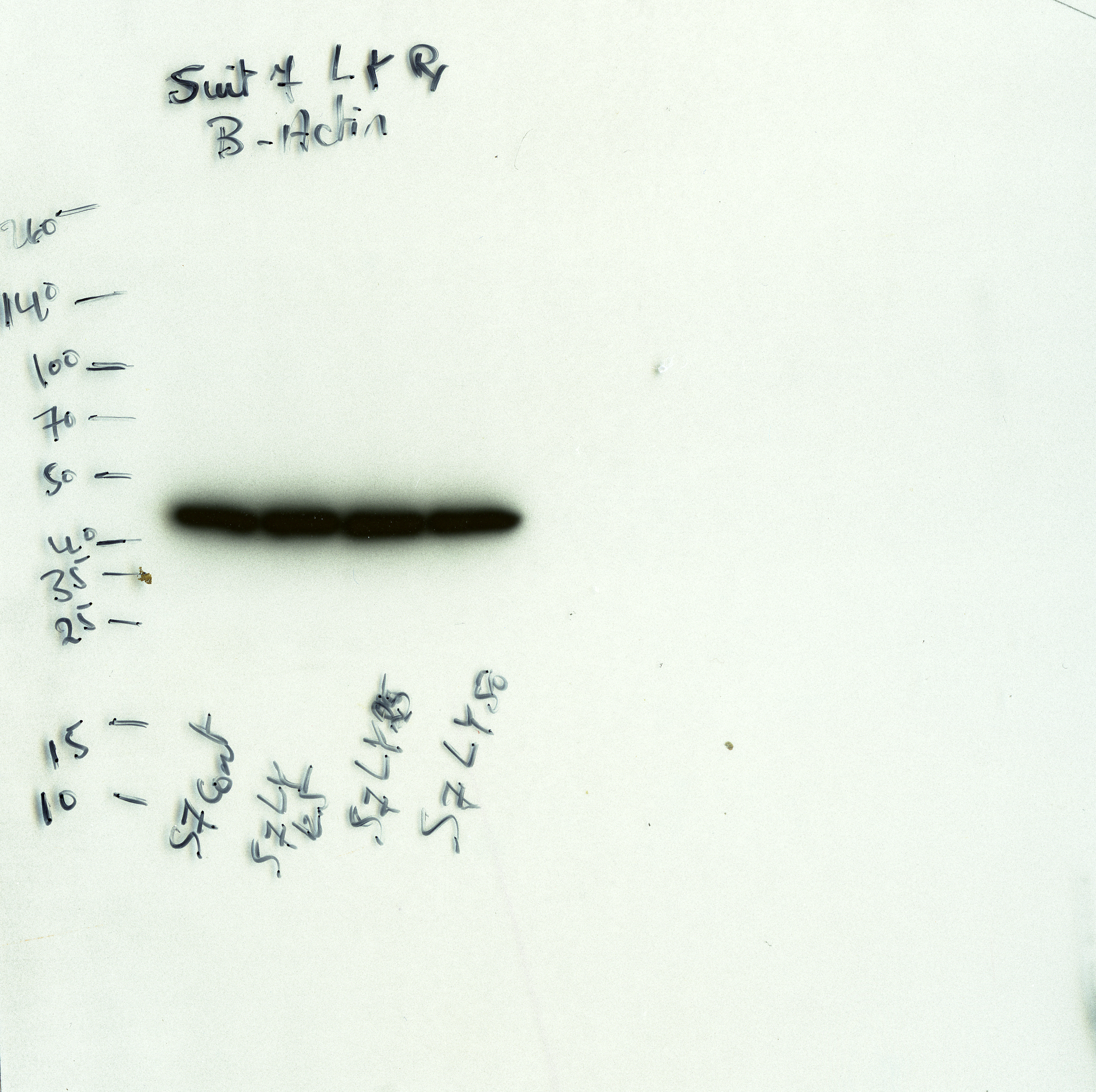

Supplement: Supplementary file 38 — WB24 [file 41420_2019_206_MOESM38_ESM.tif]

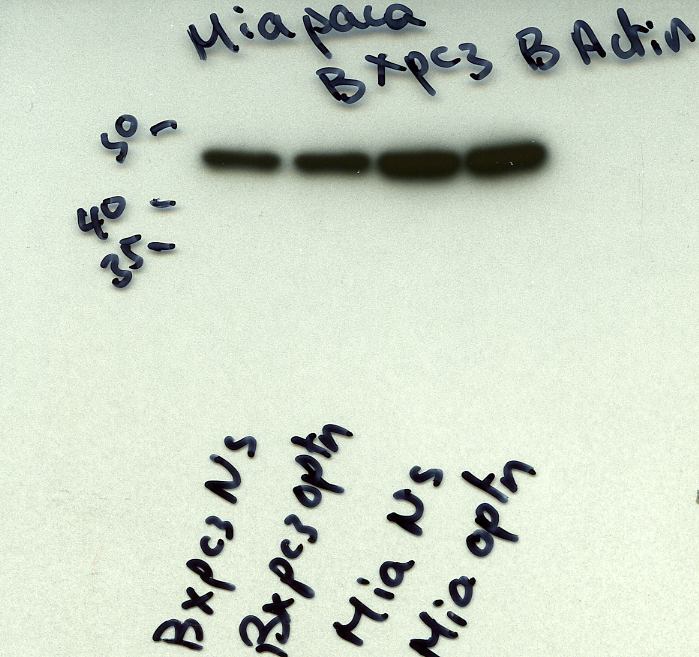

Supplement: Supplementary file 39 — WB25 [file 41420_2019_206_MOESM39_ESM.tif]

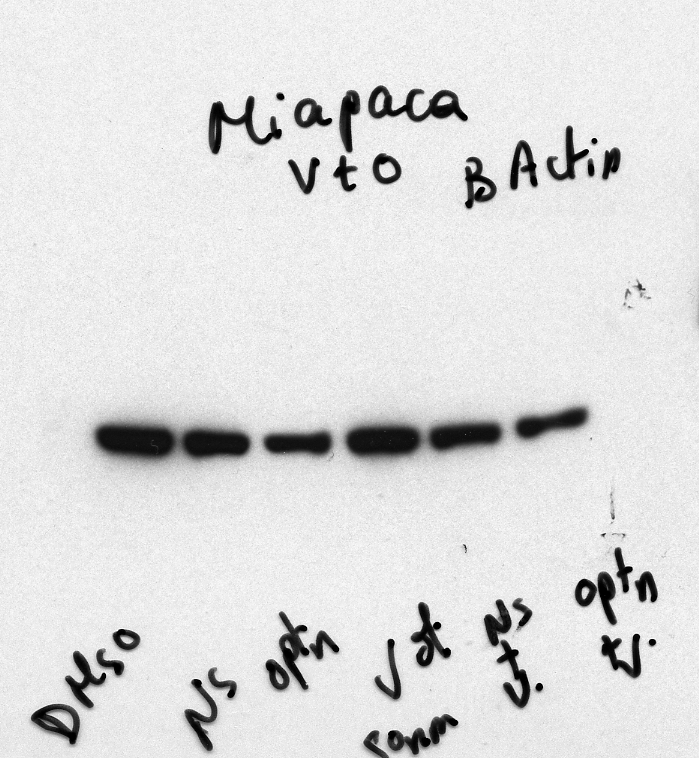

Supplement: Supplementary file 40 — WB26 [file 41420_2019_206_MOESM40_ESM.tif]

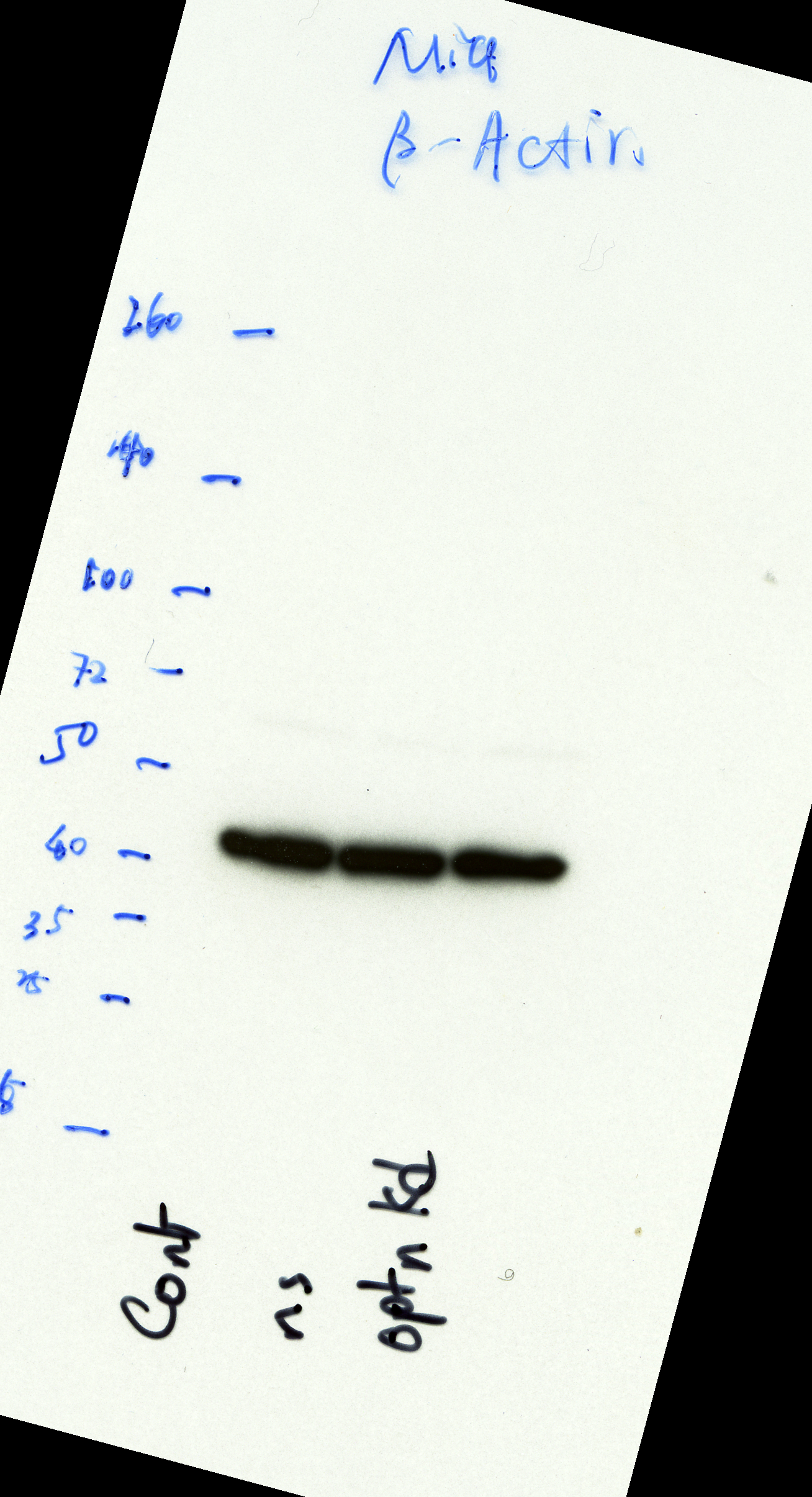

Supplement: Supplementary file 41 — WB27 [file 41420_2019_206_MOESM41_ESM.tif]

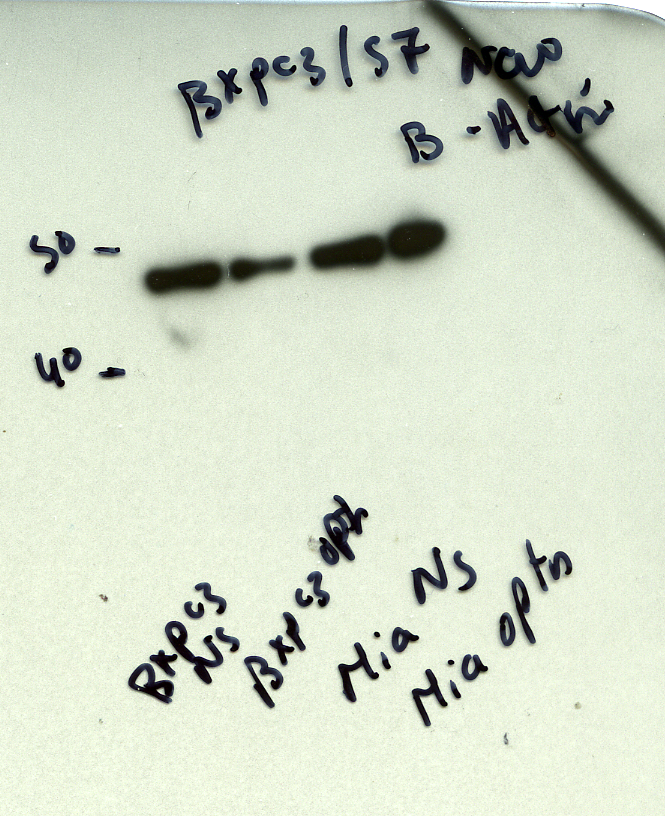

Supplement: Supplementary file 42 — WB28 [file 41420_2019_206_MOESM42_ESM.tif]

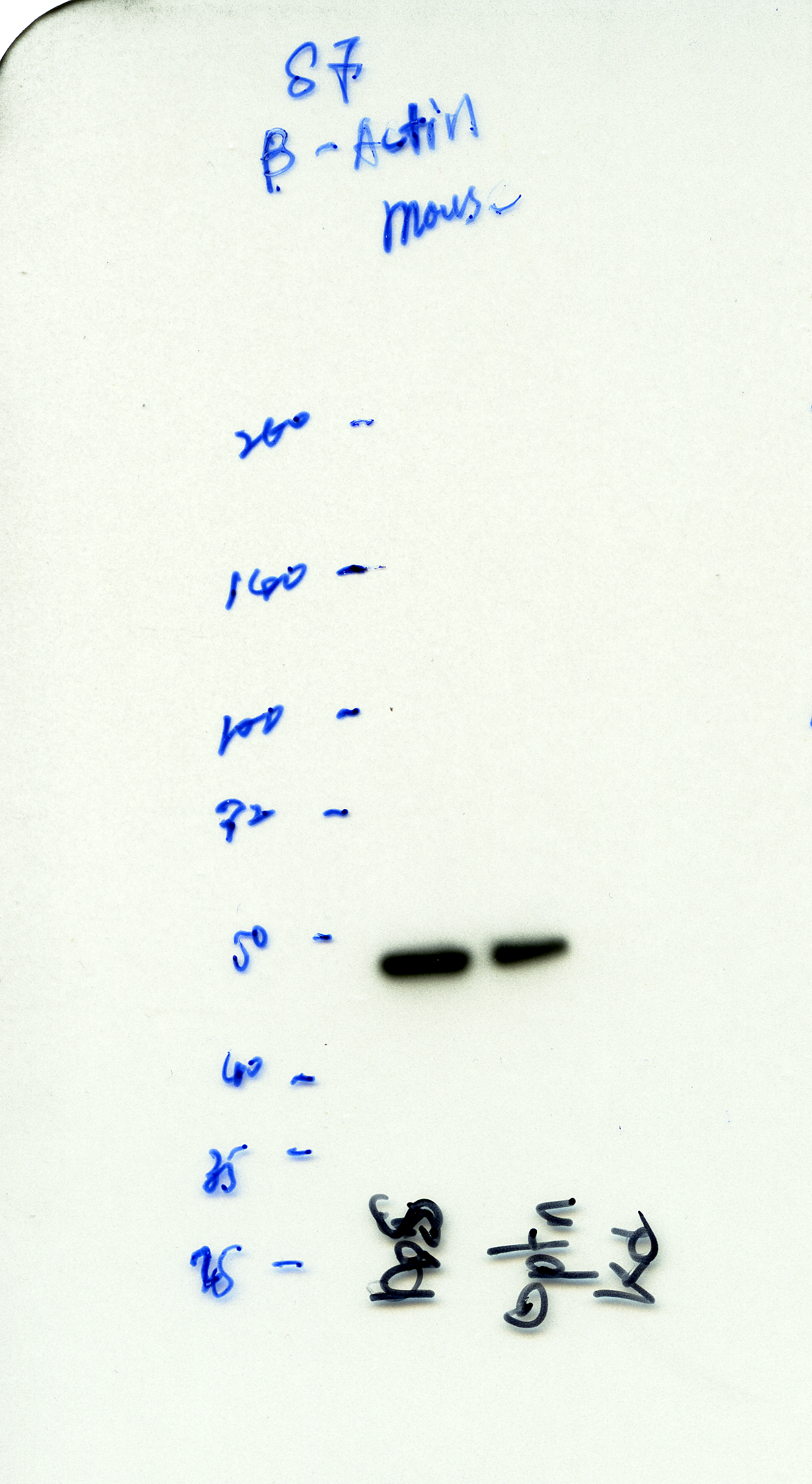

Supplement: Supplementary file 43 — WB29 [file 41420_2019_206_MOESM43_ESM.tif]

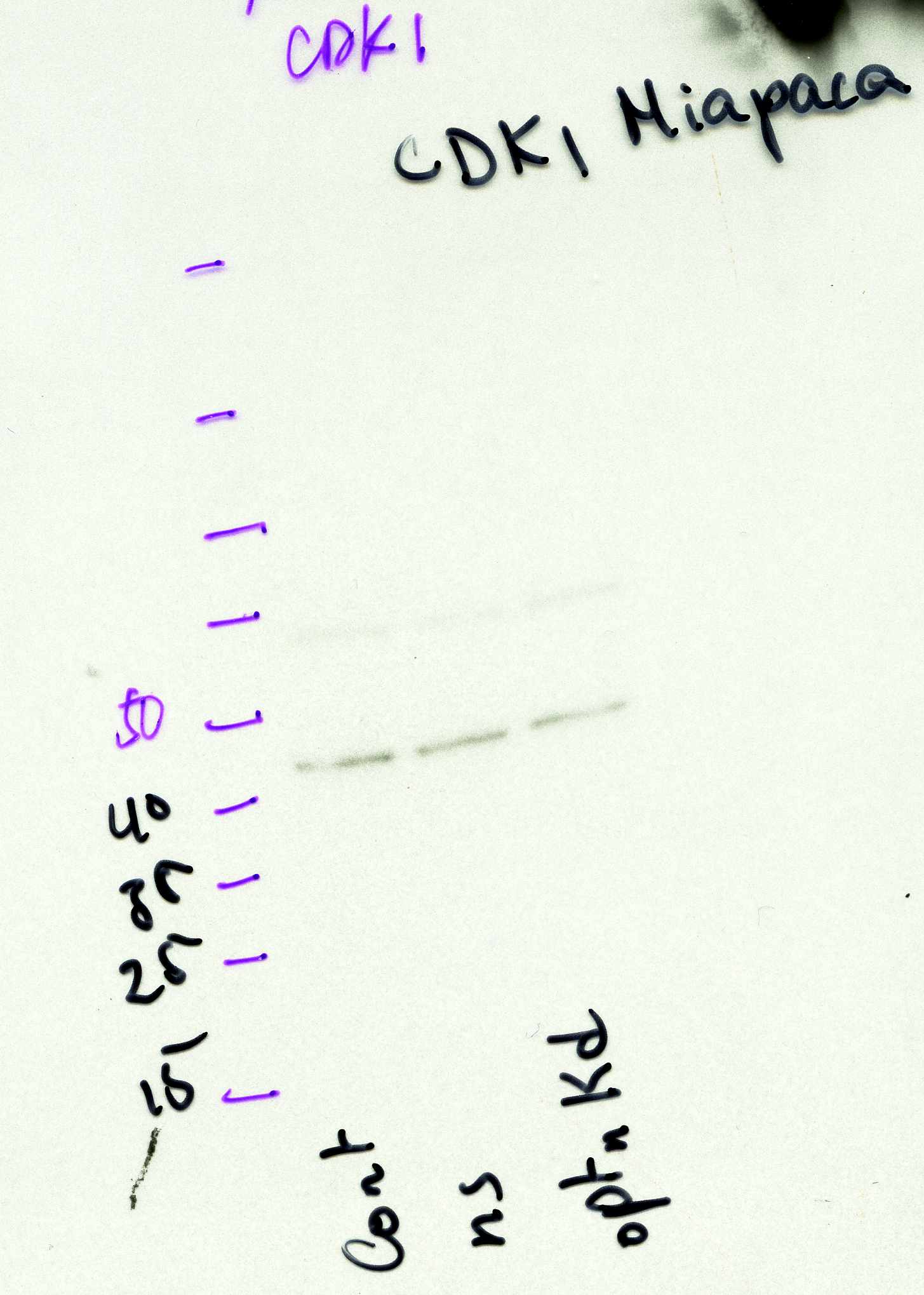

Supplement: Supplementary file 44 — WB30 [file 41420_2019_206_MOESM44_ESM.tif]

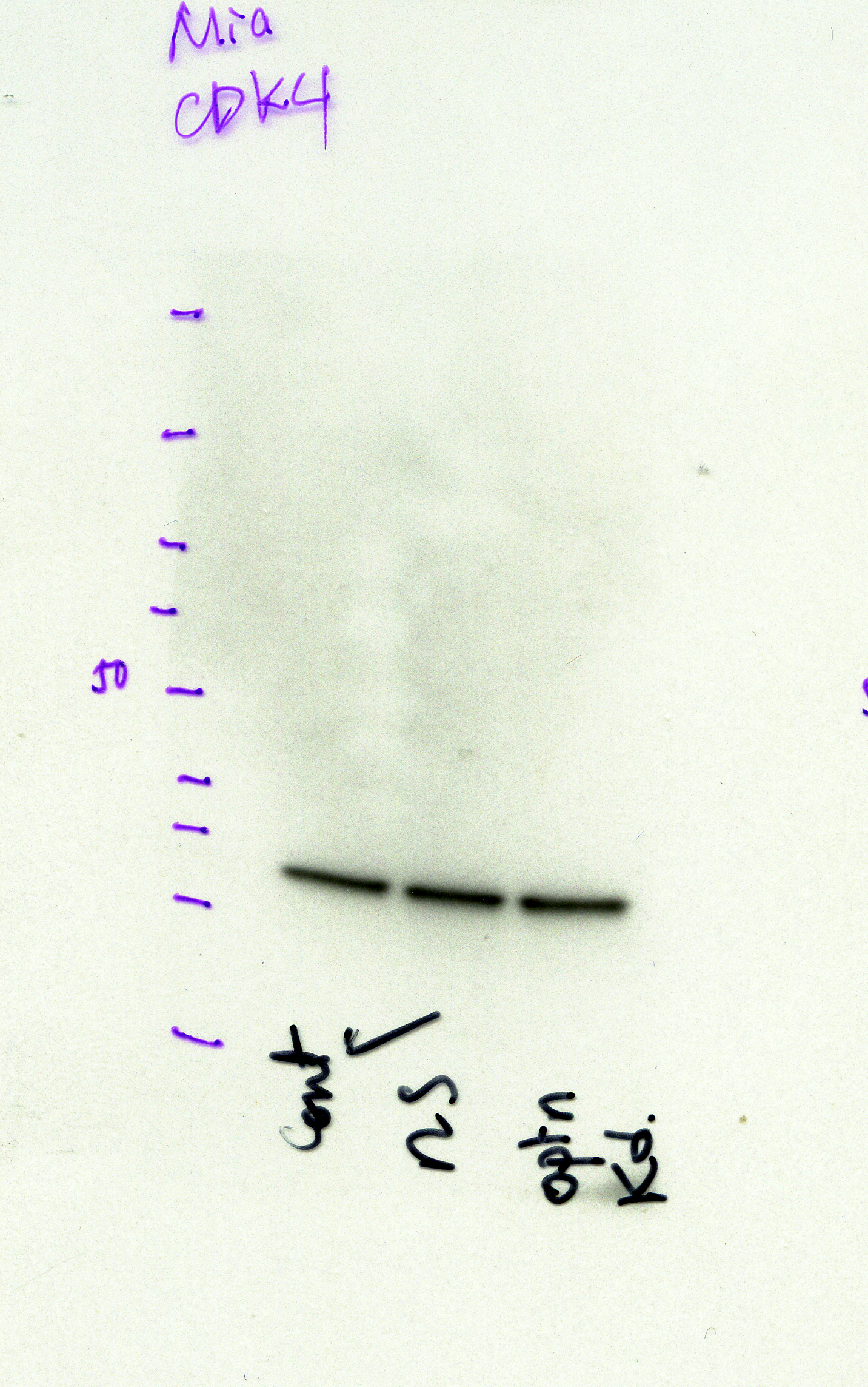

Supplement: Supplementary file 45 — WB31 [file 41420_2019_206_MOESM45_ESM.tif]

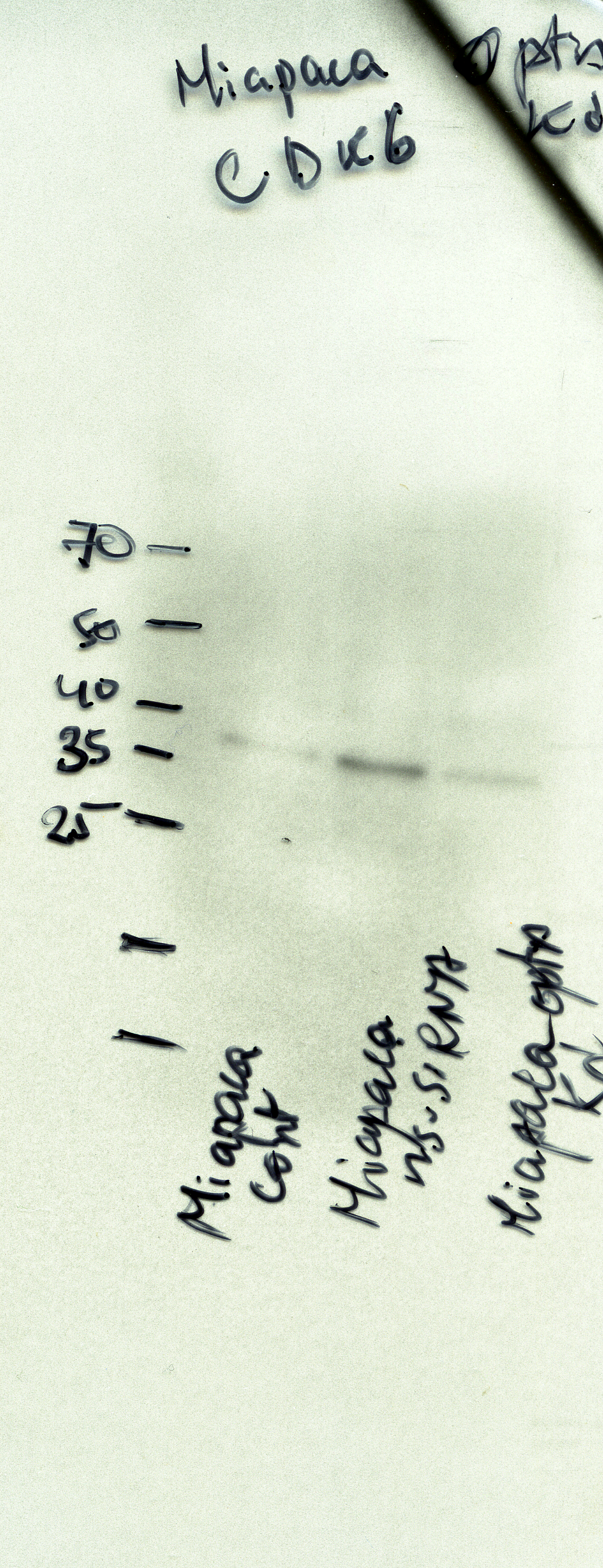

Supplement: Supplementary file 46 — WB32 [file 41420_2019_206_MOESM46_ESM.tif]

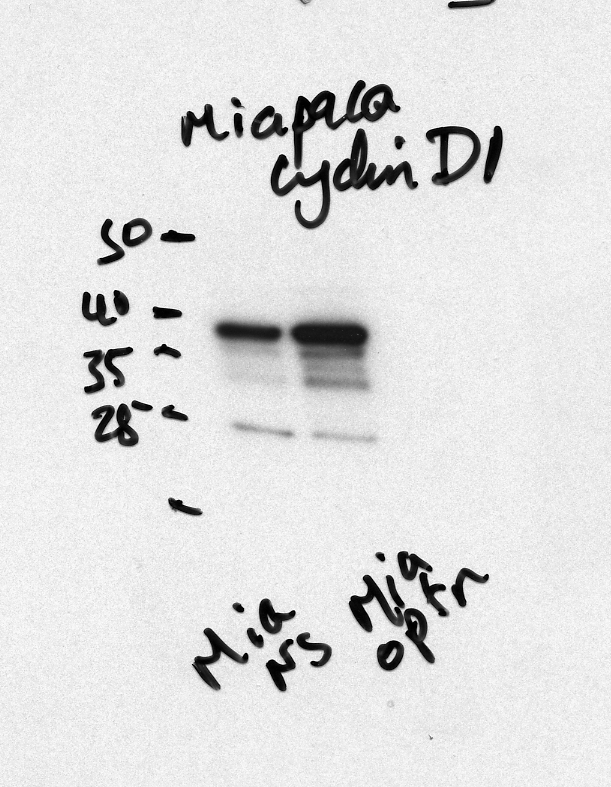

Supplement: Supplementary file 47 — WB35 [file 41420_2019_206_MOESM47_ESM.tif]

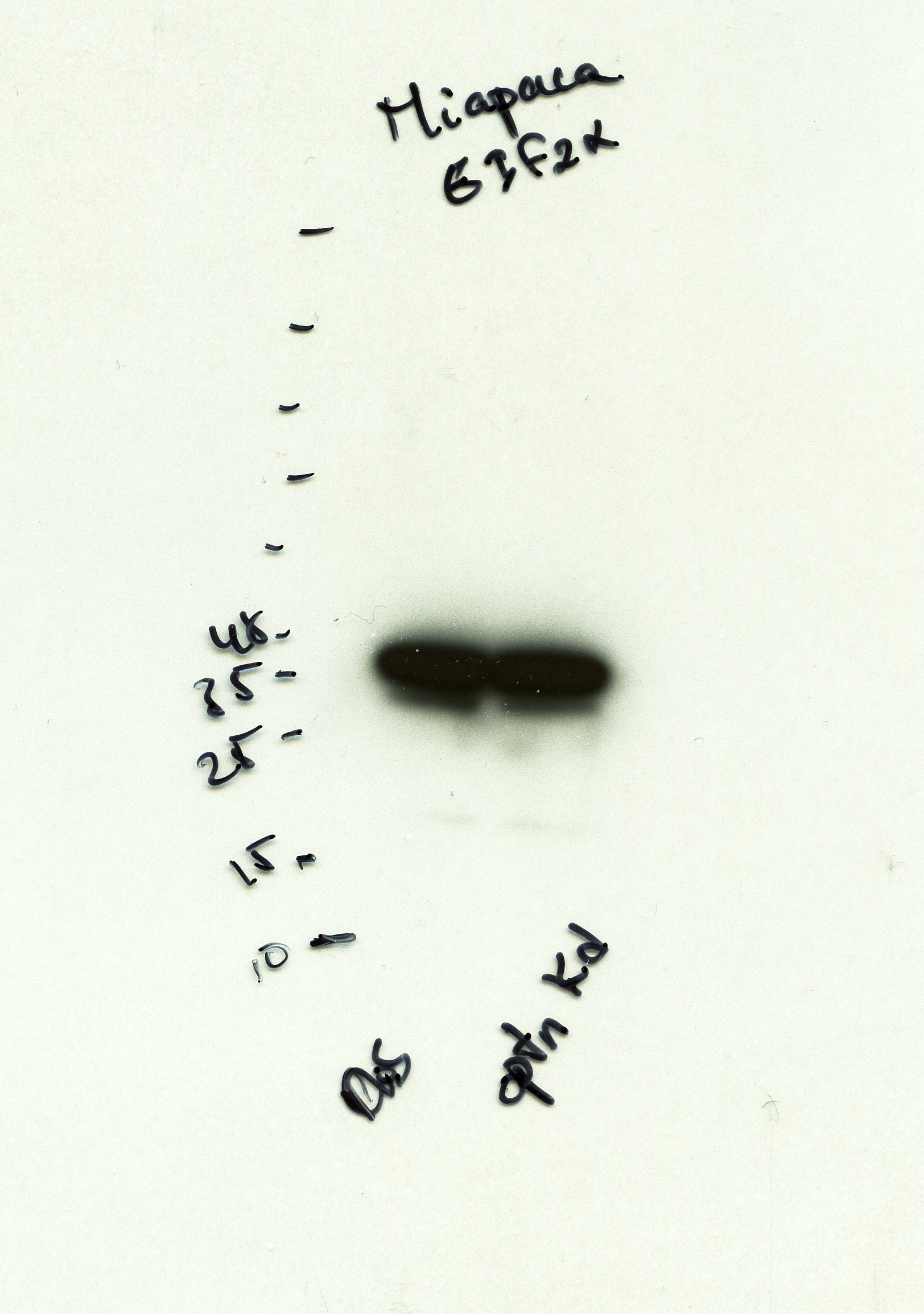

Supplement: Supplementary file 48 — WB36 [file 41420_2019_206_MOESM48_ESM.tif]

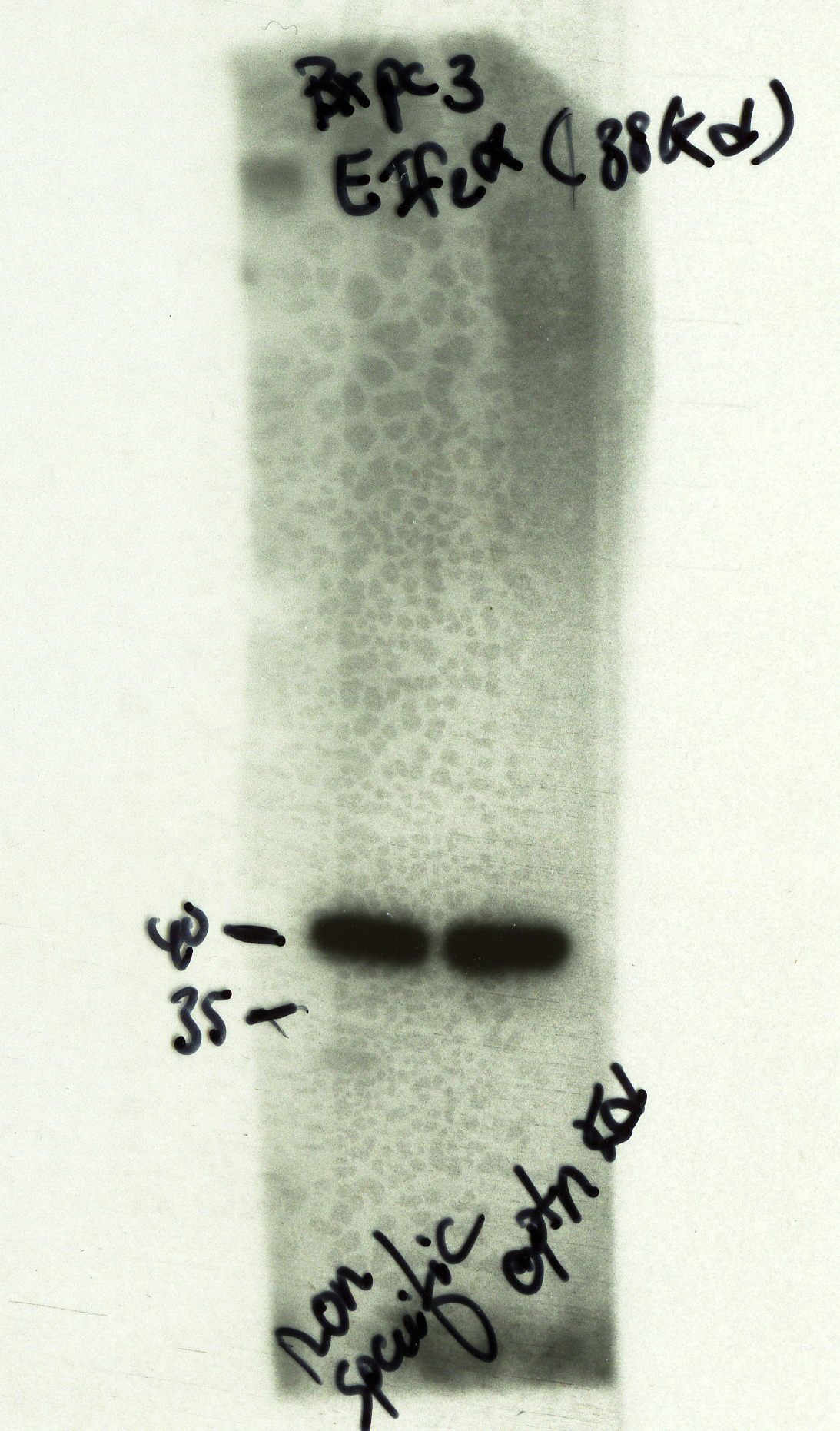

Supplement: Supplementary file 49 — WB37 [file 41420_2019_206_MOESM49_ESM.tif]

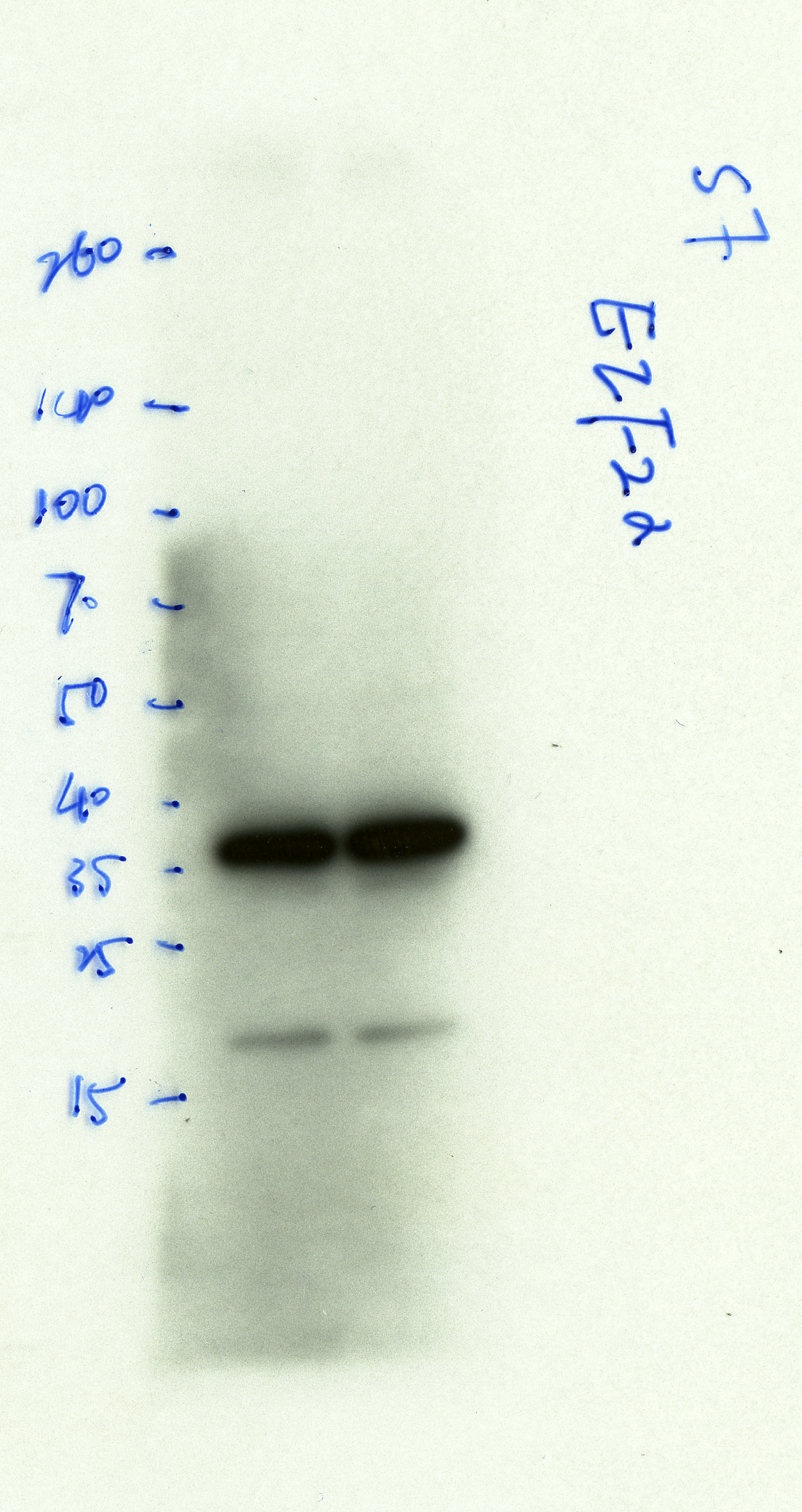

Supplement: Supplementary file 50 — WB38 [file 41420_2019_206_MOESM50_ESM.tif]

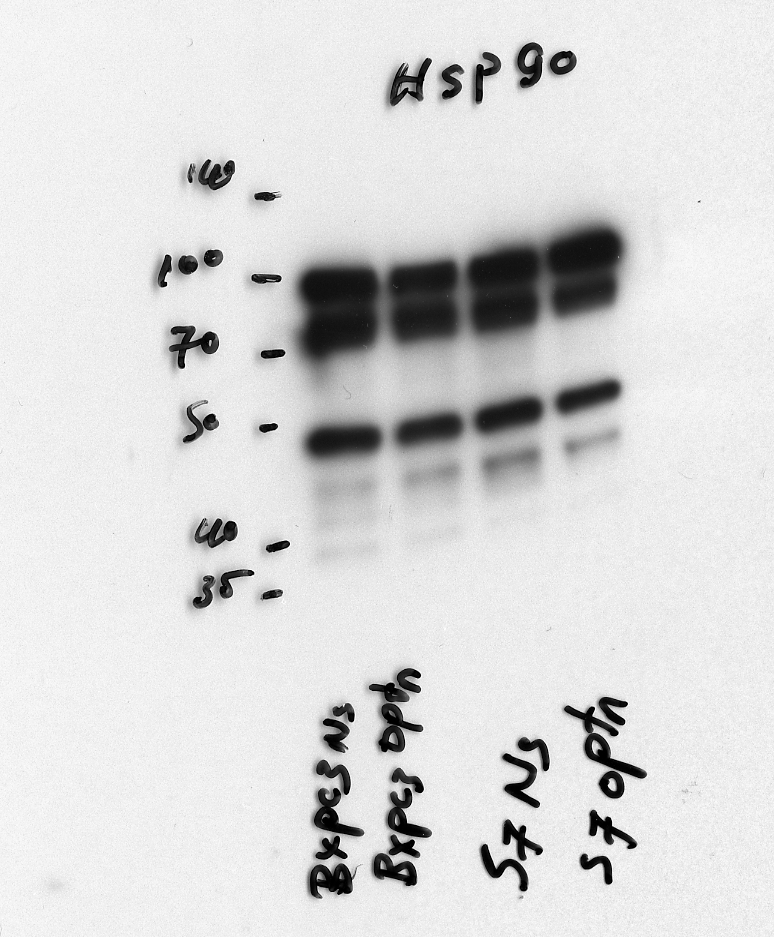

Supplement: Supplementary file 51 — WB39 [file 41420_2019_206_MOESM51_ESM.tif]

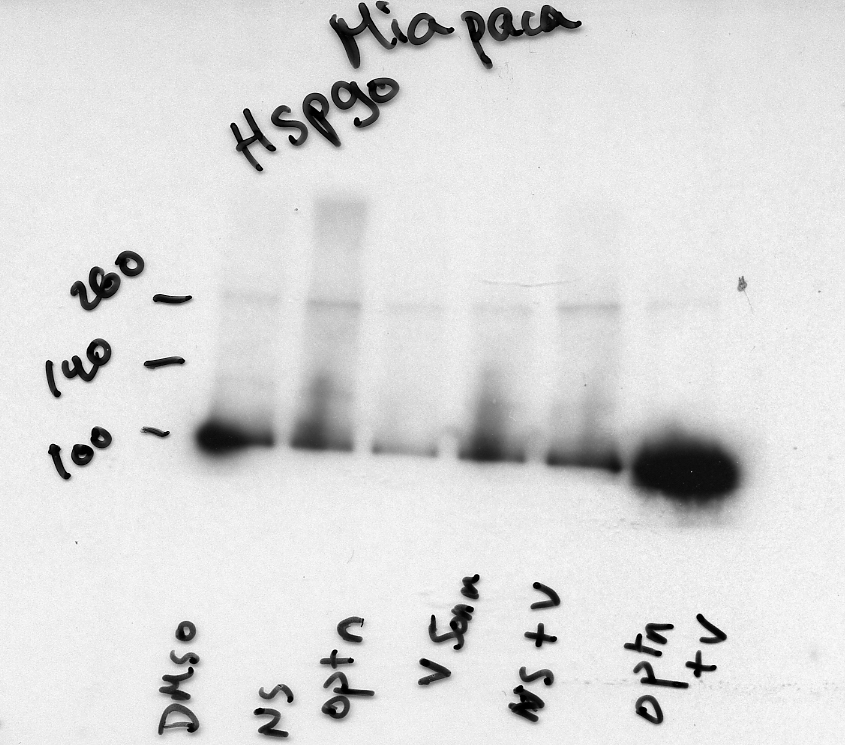

Supplement: Supplementary file 52 — WB40 [file 41420_2019_206_MOESM52_ESM.tif]

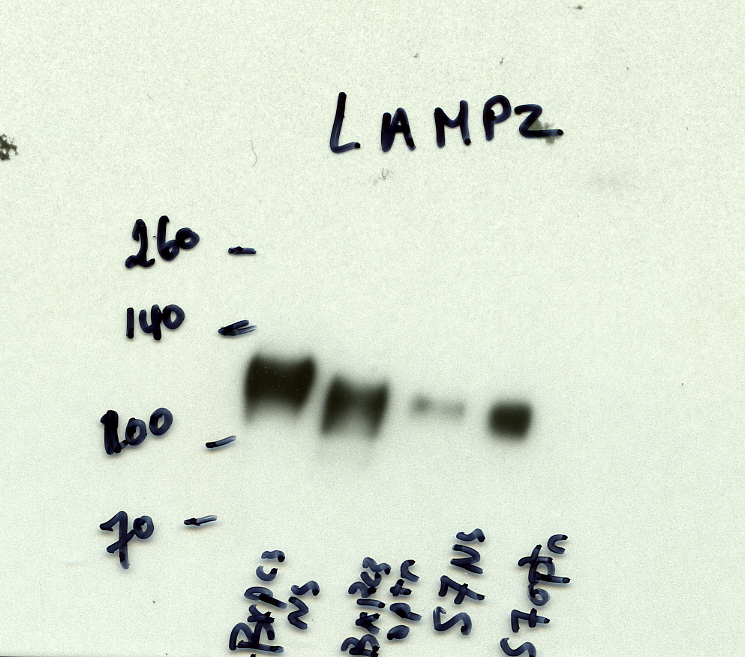

Supplement: Supplementary file 53 — WB41 [file 41420_2019_206_MOESM53_ESM.tif]

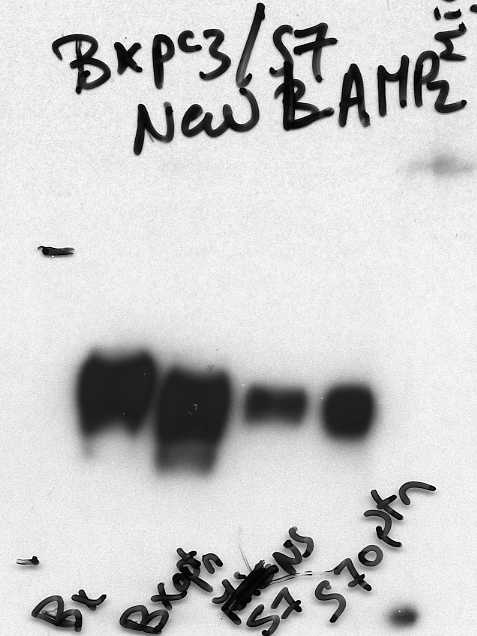

Supplement: Supplementary file 54 — WB42 [file 41420_2019_206_MOESM54_ESM.tif]

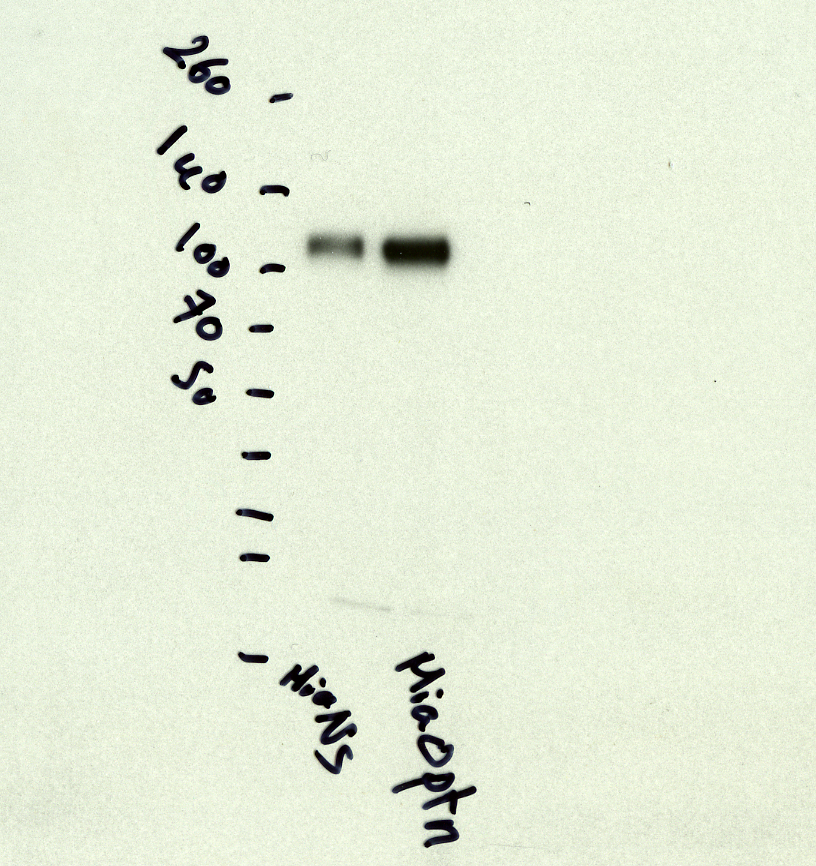

Supplement: Supplementary file 55 — WB43 [file 41420_2019_206_MOESM55_ESM.tif]

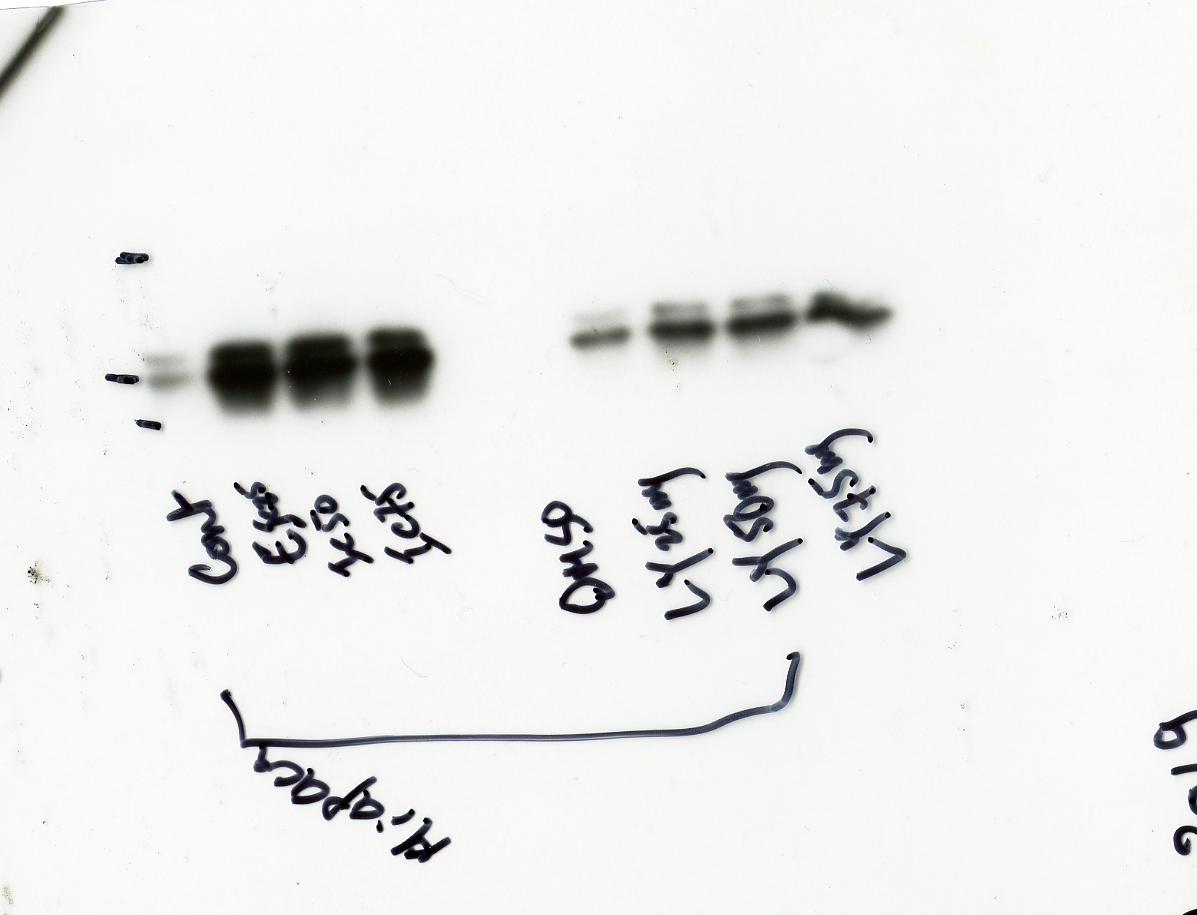

Supplement: Supplementary file 57 — WB45 [file 41420_2019_206_MOESM57_ESM.tif]

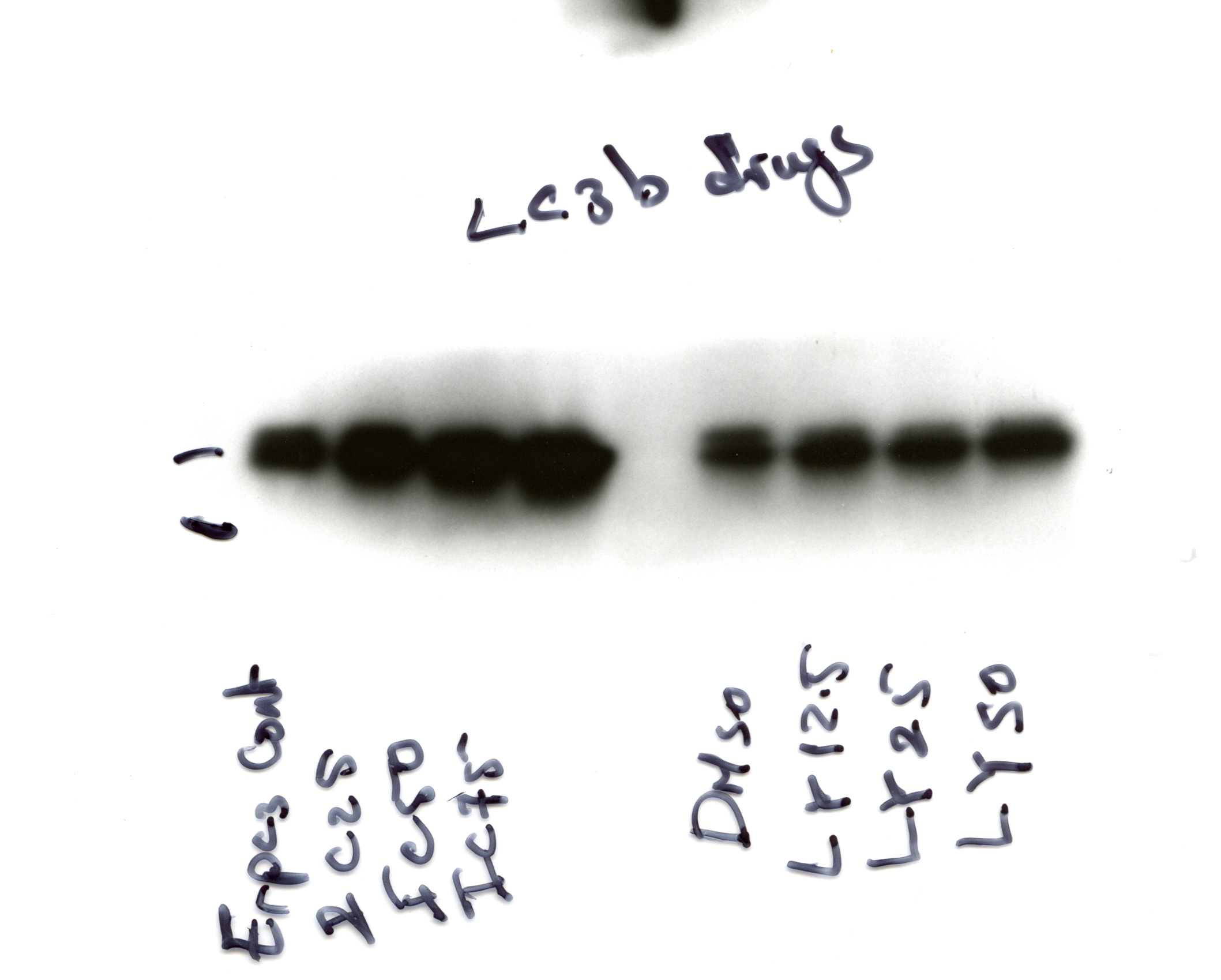

Supplement: Supplementary file 58 — WB46 [file 41420_2019_206_MOESM58_ESM.tif]

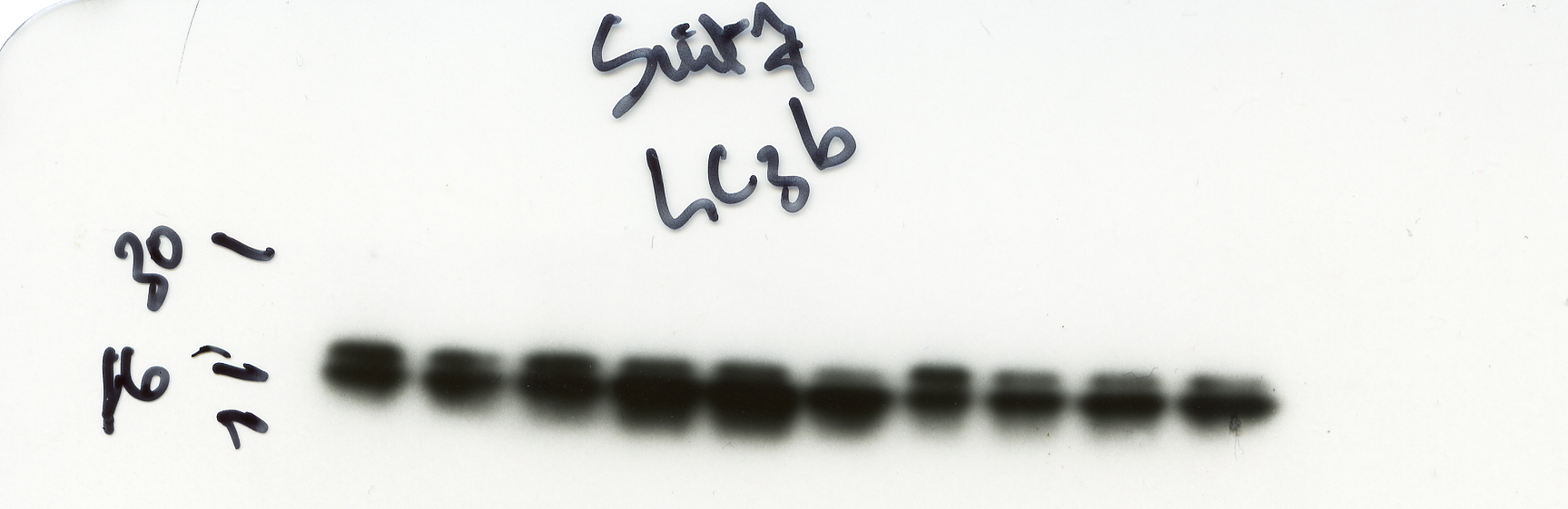

Supplement: Supplementary file 59 — WB48 [file 41420_2019_206_MOESM59_ESM.tif]

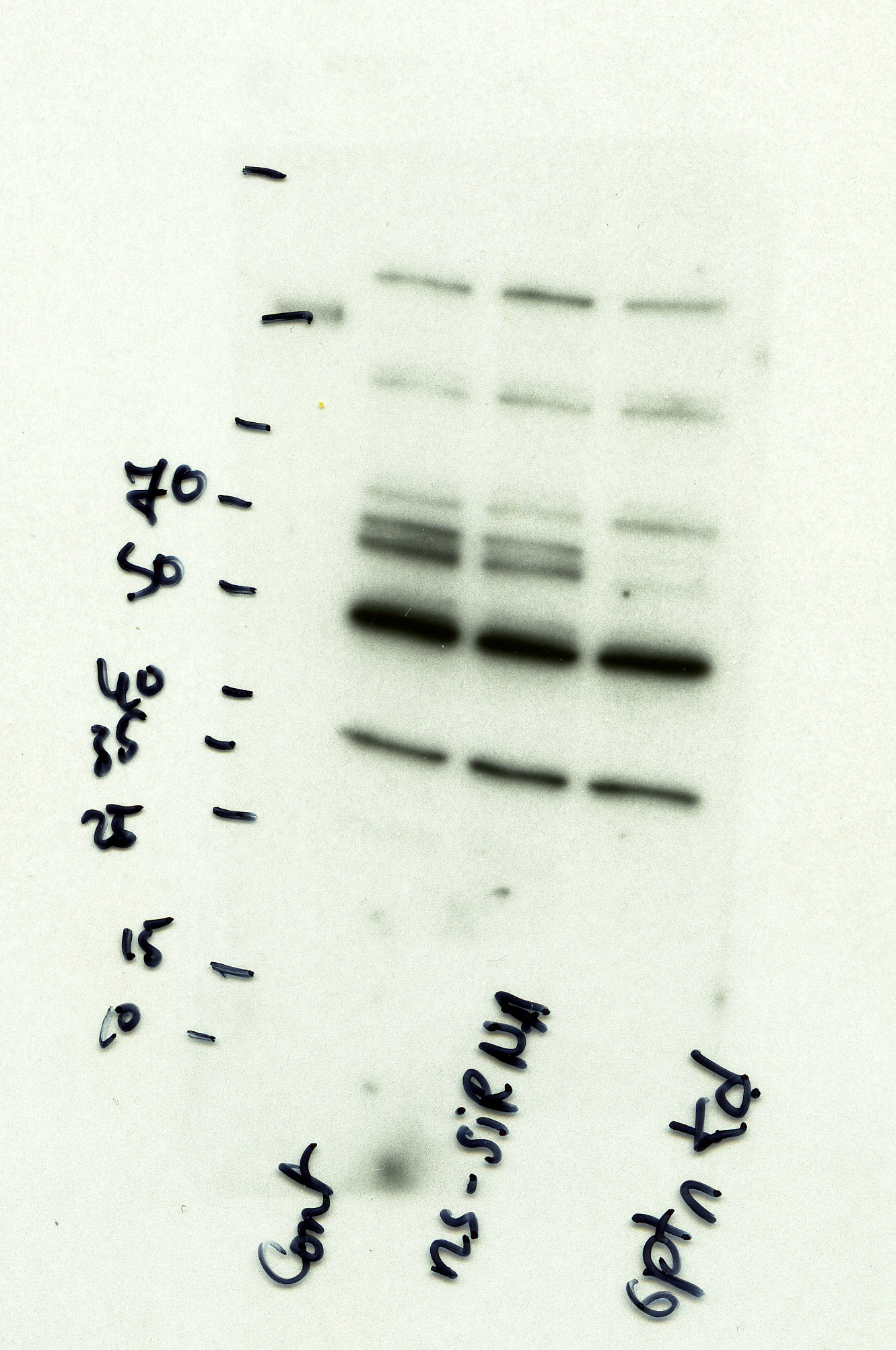

Supplement: Supplementary file 60 — WB49 [file 41420_2019_206_MOESM60_ESM.tif]

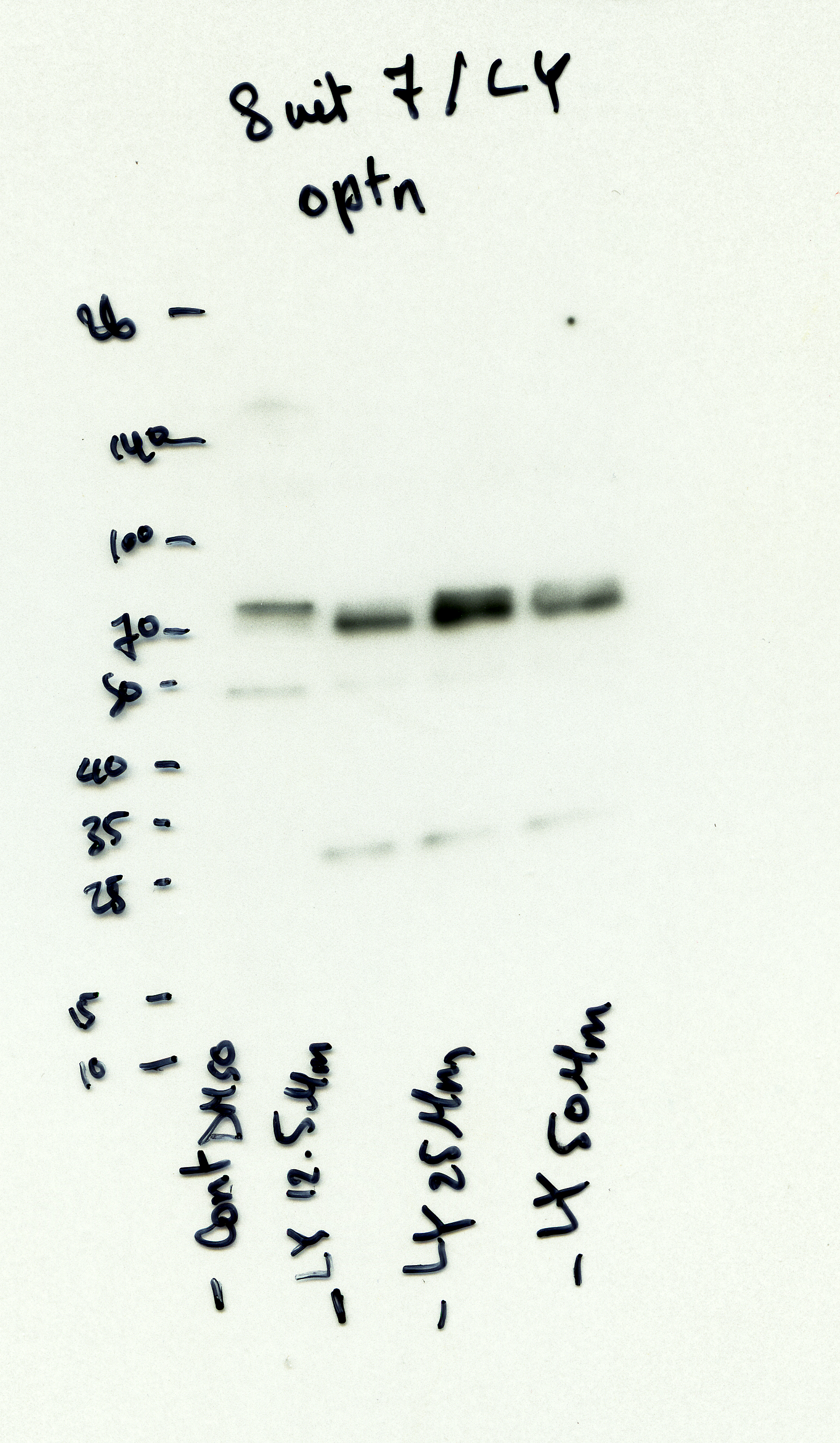

Supplement: Supplementary file 61 — WB51 [file 41420_2019_206_MOESM61_ESM.tif]

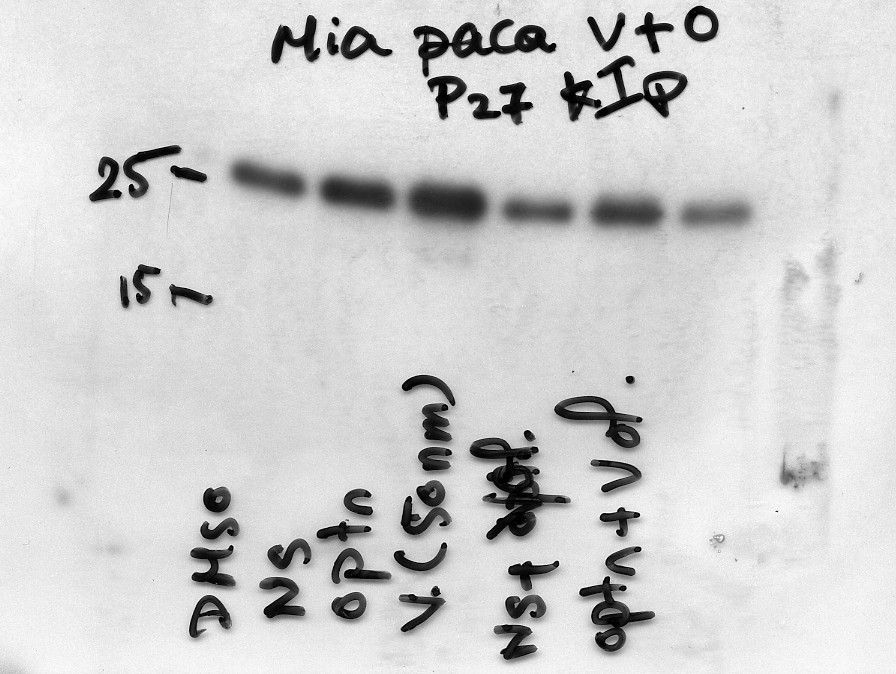

Supplement: Supplementary file 62 — WB52 [file 41420_2019_206_MOESM62_ESM.tif]

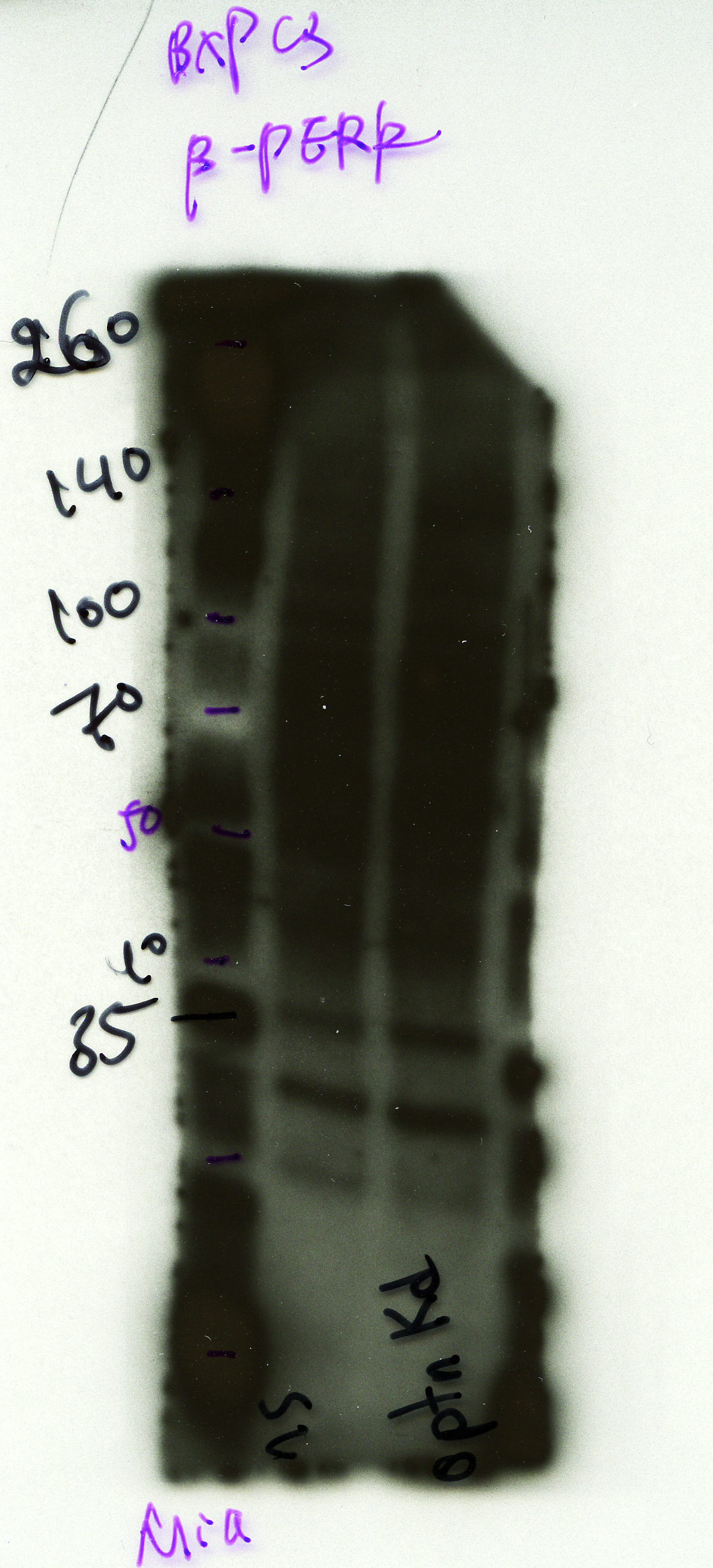

Supplement: Supplementary file 63 — WB53 [file 41420_2019_206_MOESM63_ESM.tif]

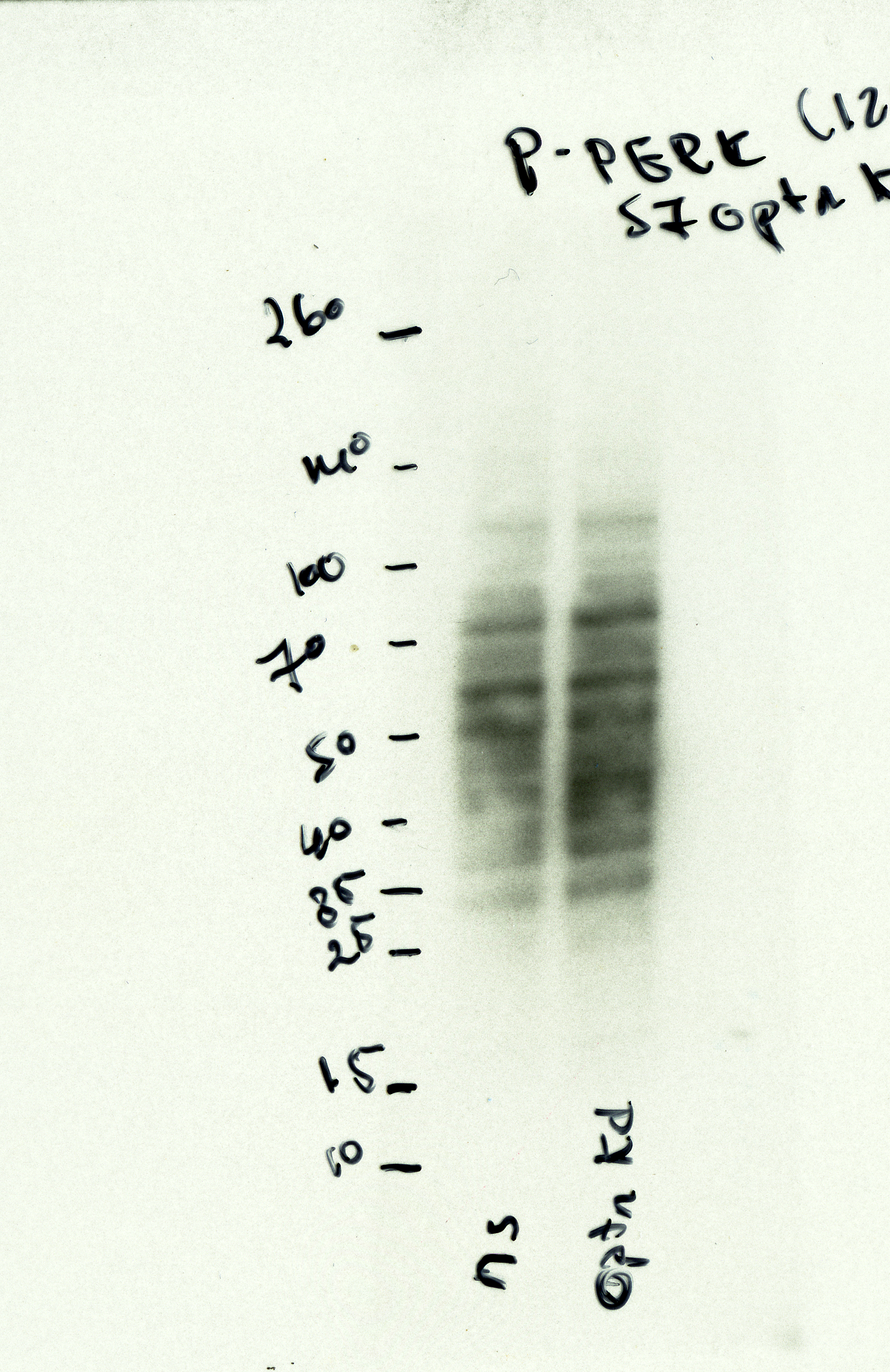

Supplement: Supplementary file 64 — WB54 [file 41420_2019_206_MOESM64_ESM.tif]

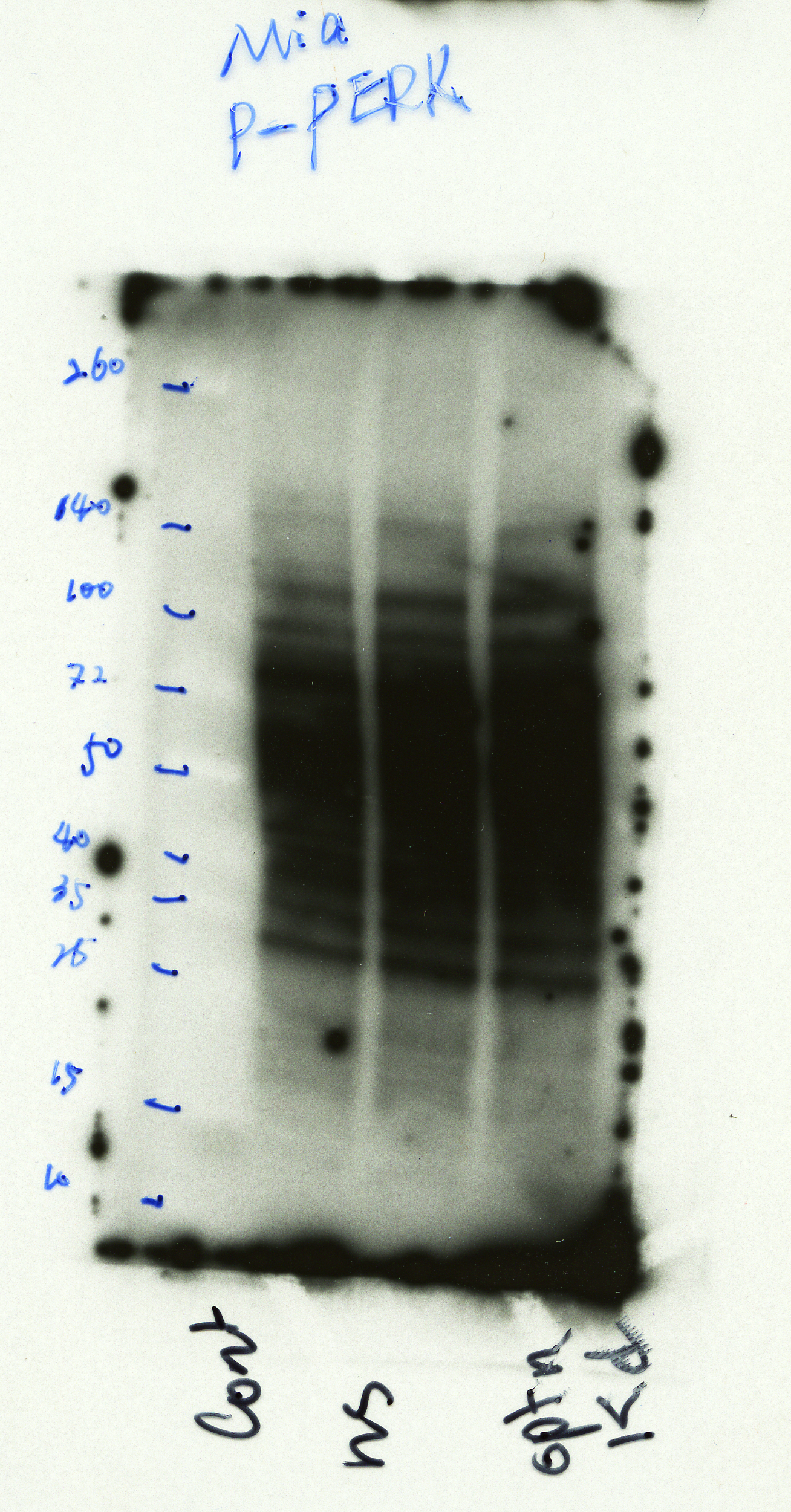

Supplement: Supplementary file 65 — WB55 [file 41420_2019_206_MOESM65_ESM.tif]
